# Living mapping review of global research funding for pandemic-prone infectious diseases– Pandemic PACT Grant Tracker

**DOI:** 10.12688/wellcomeopenres.24958.2

**Published:** 2026-07-06

**Authors:** Rodrigo Furst, Emilia Antonio, Shanthi Levanita, Zaharat Kadri-Alabi, Johannes Paolo Cerrado, Nusrat Jabin, Susan Khader Ibrahim, Thomas Mendy, Adrian Bucher, Alice Norton

**Affiliations:** 1 Policy and Practice Research Group, Pandemic Sciences Institute, University of Oxford, Oxford, Select, OX3 7BN, UK; 2 GloPID-R Research and Policy Team, Policy and Practice Research Group, Pandemic Sciences Institute, University of Oxford, Oxford, Select, OX3 7BN, UK; 3 UK Collaborative on Development Research, London, UK

**Keywords:** Living systematic review, infectious diseases, research funding, coordination, pandemic preparedness, global health policy

## Abstract

**Background:**

The COVID-19 pandemic spurred an unprecedented global research response, highlighting the need for better coordination amid limited resources and the ongoing threat of emerging diseases. The Pandemic PACT Programme has developed a live database tracking funded research on pandemic-prone diseases and broader preparedness efforts to facilitate improved pandemic preparedness. This paper presents the baseline analysis of this dataset, examining gaps in global research funding by disease, geography and research topic, and identifying which funders are supporting pandemic-prone infectious disease research.

**Methods:**

As of March 1, 2025, the database includes 20,362 grants from 326 funding organisations spread across 58 different countries, representing at least $14.4 billion in financial commitments. We conducted a mapping review to provide an open, accessible analysis of the funded research portfolio on infectious diseases with pandemic potential. The review analyses global research funding trends from 2020 to 2024 across priority diseases and research areas, identifying key investment priorities and gaps.

**Results:**

Analysis of the Pandemic PACT dataset reveals uneven global research investment across infectious diseases with pandemic potential. While funding has generally aligned with priority research areas, major research gaps persist, particularly in diagnostics, health systems research, and ethics. Although vaccine and therapeutic research dominate the portfolio, much of it remains focused on pre-clinical and early-phase trials. There is, however, increasing attention to vector and environmental research in comparison to prior analyses on COVID-19 research. Investments remain concentrated in high-income countries, with limited financial support for context-specific research led by institutions in low- and middle-income countries. The inclusion of vulnerable populations in funded grants is also limited.

**Conclusions:**

Our findings reveal critical gaps and disparities in global pandemic preparedness research funding. The living design of this review will enable ongoing monitoring of these gaps, providing real-time evidence to support funder coordination and policy decisions.

## Plain Language Summary

The COVID-19 pandemic showed how quickly infectious diseases can spread and highlighted the need for better global research coordination. The Pandemic PACT Programme has created a live database that tracks research funding on diseases that could cause future pandemics, helping to improve preparedness.

As of March 2025, the database includes over 20,000 grants from more than 300 funders across 58 countries, representing at least $14.4 billion in financial support. We reviewed this data to understand global research funding trends from 2020 to 2024, focusing on priority diseases and research areas.

Our analysis shows that funding is unevenly distributed. Vaccine and therapeutic research receive most of the financial support, but much of it focuses on early-stage clinical trials. Areas such as diagnostics, health systems research, and ethics receive comparatively less attention. Funding is largely concentrated in high-income countries, with limited support for research led by institutions in low- and middle-income countries. Vulnerable populations are also rarely included in funded studies.

These findings highlight gaps in global research investment and can help guide better coordination and decision-making to strengthen pandemic preparedness worldwide.

## Introduction

The COVID-19 pandemic triggered an unprecedented flow of funding for research, resulting in a proliferation of publications and evidence generation. However, it also posed significant challenges for coordination of funding, research implementation, and evidence synthesis. Ultimately, despite the wealth of relevant and impactful knowledge generated by this research response, duplication and redundancy were unavoidable. ^1^

In 2020, the World Health Organisation (WHO), in collaboration with Global Research Collaboration for Infectious Disease Preparedness (GloPID-R), developed the Coordinated Global Research Roadmap: 2019 Novel Coronavirus. ^2^ This roadmap outlined key knowledge gaps and research priorities identified by over 400 global experts ^3^ and was influential in the allocation of resources in the pandemic response. ^4^ The research community responded extensively at unprecedented scale, initiating numerous clinical trials for therapeutics and vaccines. However, as a result of poor design and coordination, many were conducted inefficiently limiting their actionable impact. ^5, 6^ Moreover, these research programmes and trials were predominantly launched in high-income countries (HICs), leaving low-resource settings with limited research activity despite their growing vulnerability and the need for context specific knowledge. This also highlighted the uneven distribution of global funding for research activity. ^3, 5^

In response to the critical need to coordinate and synergise funders’ research activities, GloPID-R and UK Collaborative on Development Research (UKCDR) launched the COVID-19 Research Coordination and Learning Initiative (COVID CIRCLE). This joint effort, which was founded on an agreed set of principles, tracked and mapped global research funding ^7^ to the COVID-19 research priorities outlined in the WHO Roadmap, UN Research Roadmap for the COVID-19 Recovery (UNRR), and other priority setting activities. ^8– 10^ The COVID-19 Research Project Tracker, a live database of funded research with its associated living mapping review tracked over 21,000 COVID-19 grants with at least $8.5 billion invested by over 360 global funders. ^3^ Through a living mapping review of the data captured and various funder and researcher coordination activities, COVID CIRCLE provided an overview of the COVID-19 research landscape, enhancing the identification of gaps and opportunities, and informed ongoing investments and coordination during the pandemic, which aimed to support decision makers in ensuring resources were directed towards the most pressing remaining global research needs. Its impact was highlighted in the *R&D Preparedness Ecosystem: Preparedness for Health Emergencies Report* to the Global Preparedness Monitoring Board ^11^ and in the *WHO Overall Achievements Report on COVID-19* released in 2021. ^12^

The COVID-19 pandemic highlighted pre-existing deficiencies within the global landscape of health research and development, which extend beyond the pandemic period. These limitations are indicative of broader challenges within the R&D ecosystem and have far-reaching consequences for the impact of health research, encompassing research conduct and dissemination of research findings, R&D governance, and research funding models. ^13^ Critically, COVID-19 also exposed the fragility of pandemic financing mechanisms and the structural limitations of existing preparedness funding architectures, which remain poorly suited to sustaining investment across the full range of pandemic-prone diseases between emergencies. ^14^ Effective health systems require not only increased capacity during crises but sustained, well-coordinated research investment across the full spectrum of pandemic-prone diseases in non-emergency periods. ^15^ Furthermore, the persistent challenge of monitoring the health R&D investments presents an obstacle to ensuring the effective use of limited resources in a timely manner. ^16^ This is particularly relevant in the fast-moving context of infectious disease outbreaks, where real-time visibility into research funding at the point of initiation is crucial Evidence-informed funding allocation and coordination can be enabled by providing insights into the nature of the research, its alignment to prioritised areas, its geographical distribution, and the stakeholders involved. ^16, 17^ Real-time tracking can also ensure agility in responding to emerging needs and localise effort where appropriate. With the aim of building on and improving the COVID CIRCLE initiative, we established the Pandemic Preparedness: Analytical Capacity and Funding Tracking Programme (Pandemic PACT). ^17^

Pandemic PACT has developed a tool to systematically track and analyse prospective and rolling research funding as well as broader research preparedness effort for epidemic-prone infectious diseases, as outlined in our protocol. ^18^ The tool incorporates data designed to be findable, accessible, interoperable and reusable (FAIR). ^19^ All related metadata and database are made available in a machine-actionable and user-friendly interface. This original database of global funding for pandemic-prone disease research is updated regularly and linked to publications through Europe PMC, is aligned to a standardised research ontology and mapped to the relevant policy roadmaps. The programme seeks to facilitate evidence-informed funding and research activation by providing timely information that can enhance the efficient allocation of limited research resources and enable a coherent global research response, thereby strengthening the global health research system. ^18^

Despite the lessons of COVID-19, a comprehensive, real-time analysis of how research funding is distributed across the full range of pandemic-prone infectious diseases remains absent from the literature, leaving a gap in our understanding of the broader global research investment landscape and its alignment with preparedness priorities. This paper presents the first analysis for the research grant funding tracking component of Pandemic PACT, which will form the baseline for our living mapping review (LMR) that will be regularly updated incorporating newly funded research grants as they become available. The key research questions we aim to answer are (i) “where are the gaps in the global distribution of research funding for infectious disease with a pandemic-potential disaggregated by disease, geography, and research topic?”, and (ii) “which funders are supporting infectious disease research globally, and, specifically, in low and middle-income countries (LMICs)?”

## Methods

### Protocol updates

Our methodology is documented in our previously published protocol. ^18^ This includes the eligibility criteria, data sources and search strategy, data flow and processes, database structure, data governance, and data updates and archiving. Methodological details provided in this paper include updates and specificities of this baseline analysis.

A key update to the protocol is the expanded coverage of funding sources, which now includes a broader range of non-commercial funders beyond those affiliated with GloPID-R and UKCDR. The complete list of funders captured in the Pandemic PACT dataset is provided in Extended Data 1, while Extended Data 2 details the data sources and the date of the last search. Specifically, the first table lists the funding databases from which data were directly and systematically extracted, whereas the second documents additional sources identified indirectly through those primary databases.

Further, we modified our approach to grant categorisation to accommodate grants that lack sufficient information to be categorised or fall into research areas outside our framework. Specifically, we distinguished between two types of cases: grants for which information was insufficient, and grants that clearly did not fit any of the predefined categories. The former was coded as “unspecified,” reflecting the absence of sufficient information, whereas the latter were assigned to “N/A,” indicating a mismatch with our existing classification structure rather than missing data, often corresponding to emerging or cross-cutting research directions.

### Rationale for using a living method

The COVID-19 pandemic prompted a swift response from research funding organisations, supporting both new research projects through rolling and one-off funding opportunities and also repurposing existing grants. This reflects the dynamic and evolving nature of infectious disease funding landscape, which is anticipated to witness continued expansion of funding for ‘priority diseases’ and reactive funding whenever there are outbreaks. Furthermore, funding calls often have short timeframes in outbreak scenarios and allocations may be influenced by rapidly changing needs and contexts.

To capture this dynamic landscape consistently and in near real time, newly funded research grants are sourced monthly from funder databases and added to the Pandemic PACT database in batches as they become available from funders. The living mapping review is subsequently updated at six-month intervals under normal circumstances, providing regular snapshots of the evolving global research funding portfolio. This will assist the coordination of ongoing responses from researchers and funders by ensuring that up-to-date information on investment priorities and gaps remains openly accessible. The review is also designed to respond to the accelerated demands of acute health emergencies. In the event of a major outbreak, analyses for the specific disease will be prioritised within the established review cycle, allowing timely insights to be generated for the most pressing public health needs. During a pandemic, the full living mapping review will transition to three-monthly updates, ensuring that the frequency of evidence synthesis keeps pace with the speed of the research and funding response.

### Eligibility criteria

***Start date.*** Newly funded and repurposed grants with a start date on or after January 1, 2020 and retrieved from the funders before March 1, 2025, were included in this baseline analysis, to align with the start of COVID-19 research funding initiatives and the preceding COVID CIRCLE dataset. Grants without any available information on the award or start date were not included in this baseline analysis. We may review this inclusion criteria in future analyses to determine how to leverage other available data for grants that are lacking sufficient information.

***Funders.*** The funders were identified based on their inclusion in the previous COVID-19 funding tracker or their collaborations with other funders, such as participation in joint funding initiatives and membership in funder networks.

***Diseases/pathogens.*** We have included all diseases listed on the WHO R&D Blueprint 2020 ^2^ priority disease: COVID-19; Crimean-Congo haemorrhagic fever; Ebola and Marburg virus disease; Lassa fever; Middle East respiratory syndrome coronavirus (MERS-CoV) and Severe Acute Respiratory Syndrome (SARS); Nipah and henipaviral disease; Rift Valley fever; Zika virus disease and Disease “X”. Disease “X”, is a concept rather than a specific disease, which represents “the knowledge that a serious international outbreak could be caused by a pathogen currently unknown to cause human disease”. ^20^ Three additional diseases (Pandemic-prone influenza, Mpox and plague) were included following the advice of our expert advisory group.

In 2024, WHO Published the Pathogen Prioritization: *A Scientific Framework for Epidemic and Pandemic Research Preparedness* Report ^21^ which outlines a new approach of prioritisation of high-risk pathogens focusing on entire pathogen Families. The Pandemic PACT programme is now working to expand its scope to this new framework and future updates to this review will therefore include this expansion.

### Data processes

Data was sourced directly from research funders using a standardised data collection template or scraped from their databases and was stored in its original format. Basic cleaning to remove special characters was done to improve the clarity of grant information. Researchers with expertise in public health reviewed and classified the data according to the Pandemic PACT schema. The list and descriptions of research categories to which grants were classified is presented in the Extended Data 3. This is an updated list from our published research categorisation ontology outlined in Norton et al. ^17^ and the variables were introduced at a higher level in the Pandemic PACT protocol. ^18^ Data underwent peer review by at least two coders, ensuring independent scrutiny. Disagreements were resolved through joint discussions. The standardised training of the annotators and collaborative meetings ensured consistency of processes. In this baseline analysis, data was exported from the Pandemic PACT dashboard. We undertook descriptive analyses of the data to summarise our findings.

### Limitations

A key limitation of the database is that the data from each funder has varying available information, which can result in less refined categorisation when the qualitative details provided were insufficient (for example some funders only list grant titles and do not publish abstracts). Therefore, the values assigned by annotators may not have fully captured all aspects of the grants in relation to research areas of focus, funding amounts, study populations, and study type and other relevant fields in the database. To address this, the data was validated by reviewers with an array of expertise including global health research, policy, and funding, and other areas ensuring that the assigned values were as accurate as possible given the available information. While the database is limited by incomplete funding information (financial data were available for only 68.7% of the grants analysed) it represents the most comprehensive resource currently available, having drawn on all publicly accessible sources. Another key limitation is that we are unable to trace financial flows beyond the lead institutions, which restricts our ability to fully capture the downstream allocation of funds. Additionally, many grants span multiple countries, research areas, pathogens, and diseases without disclosing how funds are distributed across these categories. This lack of granularity poses challenges for financial analysis. To address this, we provide both potential and minimum funding estimates: potential values assume that the full amount of a grant is allocated to each relevant category, while minimum values include only grants that are exclusively focused on a single variable (e.g., research area, disease, or location). This approach offers a more nuanced understanding of funding distributions while acknowledging the limitations of the available data.

Furthermore, engaging with (and obtaining data from) a broader range of funders than those included proved challenging, either due to lack of publicly available contacts or limited capacity from the funders to contribute to the grants (especially funders with non-English award information). This challenge is compounded by the dynamic nature of the database which expand continually to keep pace with the evolving landscape of research funding for infectious disease with pandemic potential.

Whilst most research funders operate as independent entities, mechanisms for funding including funding sources and flows vary and can interrelate across various funding entities and funding schemes. In this analysis, we do not account for funding relationships or funding flows among funders unless where entities are explicitly listed as co-funders of grants. Therefore, contributions of some funders to other entities or funding schemes (which are not explicit) are not reckoned against their total funding investment resulting in an underestimation of their contributions in these instances.

The term grant is used instead of project, because a single research project may receive multiple funding sources, each with its own unique identifier or grant ID. This means our data analysis might not be able to accurately reflect the number of distinct projects that focus on a particular research priority area.

The search terms used to retrieve the grants for the database are in English which could pose limitations; however, these standard Latin characters search terms are aligned to global ontologies and are therefore universal.

### Risk of bias

This baseline analysis employs descriptive and thematic analyses to summarise the scope of these funded grants on research on pandemic-prone diseases. No efforts were made to assess the quality of individual studies or their ability to achieve stated objectives. Potential sources of bias from grant selection, quality of reviewed data, and data extraction and classification are addressed through robust monthly searches, template completion by funders and independent assessment and review during project classification respectively, as mentioned in the Information Sources and Search Strategy.

While the tracker and the subsequent analysis aim to provide a comprehensive overview of the research landscape for infectious disease with pandemic potential, the data collected is more likely to be derived from funders of research that are members of UKCDR (all UK-based) and/or GloPID-R (global membership spanning high income countries (HICs) to low-income countries (LICs) with the majority of national funders based in HICs, ^3^ and a biomedical focus). This may likely result in the findings skewing towards a greater representation of research funded from these organisations and reflecting trends in their respective portfolios (in terms of location, research focus and research activity type) rather than necessarily mirroring the broader landscape.

### Scope of analysis

Due to the disproportionate allocation of funding toward COVID-19, our main analyses in this paper focus on grants that include at least one of the other diseases listed in the Pandemic PACT framework, using grants entirely dedicated to COVID-19 solely as a comparator reference point. This decision was motivated by the consideration that Pandemic PACT data builds on the COVID CIRCLE database (representing 73.9% of the data in Pandemic PACT) and associated Living Mapping Review ^3^ meaning that several comprehensive analyses of COVID-19 funding have already been conducted.

## Results

### Grant selection

A total of 235,534 grants were initially retrieved from various web-based data sources using predefined search terms, and screened against the eligibility criteria described in the *Methods* section. An additional 17,632 grants from the COVID CIRCLE dataset were also incorporated. Following data aggregation, deduplication and automated pre-screening were conducted using internally developed Python-based data processing pipelines. These pipelines applied rule-based and metadata-driven approaches to identify and remove duplicate records using combinations of key fields, including grant identifiers, grant titles, abstracts, funder information, and other associated metadata. Where unique identifiers were unavailable or inconsistent across sources, text-based similarity matching (e.g., on titles and abstracts) was used to detect potential duplicates.

Automated pre-screening procedures were also applied to exclude clearly out-of-scope records based on predefined eligibility criteria, including relevance to pathogens within scope and the nature of the funded activity. In addition, automated processes supported data standardisation and harmonisation, such as normalising funder names, research institutions, and date formats prior to further review. Following the application of these automated procedures, 224,854 records were excluded. The remaining 28,312 grants were imported into our primary database (REDCap) for manual review and validation. During this process, 7,950 grants were excluded, primarily for not meeting the eligibility criteria or for not focusing on pathogens included in our scope. The final dataset used for analysis comprised 20,362 grants. A summary of this process is presented in the PRISMA flow diagram ( Figure 1).

**Figure 1.**
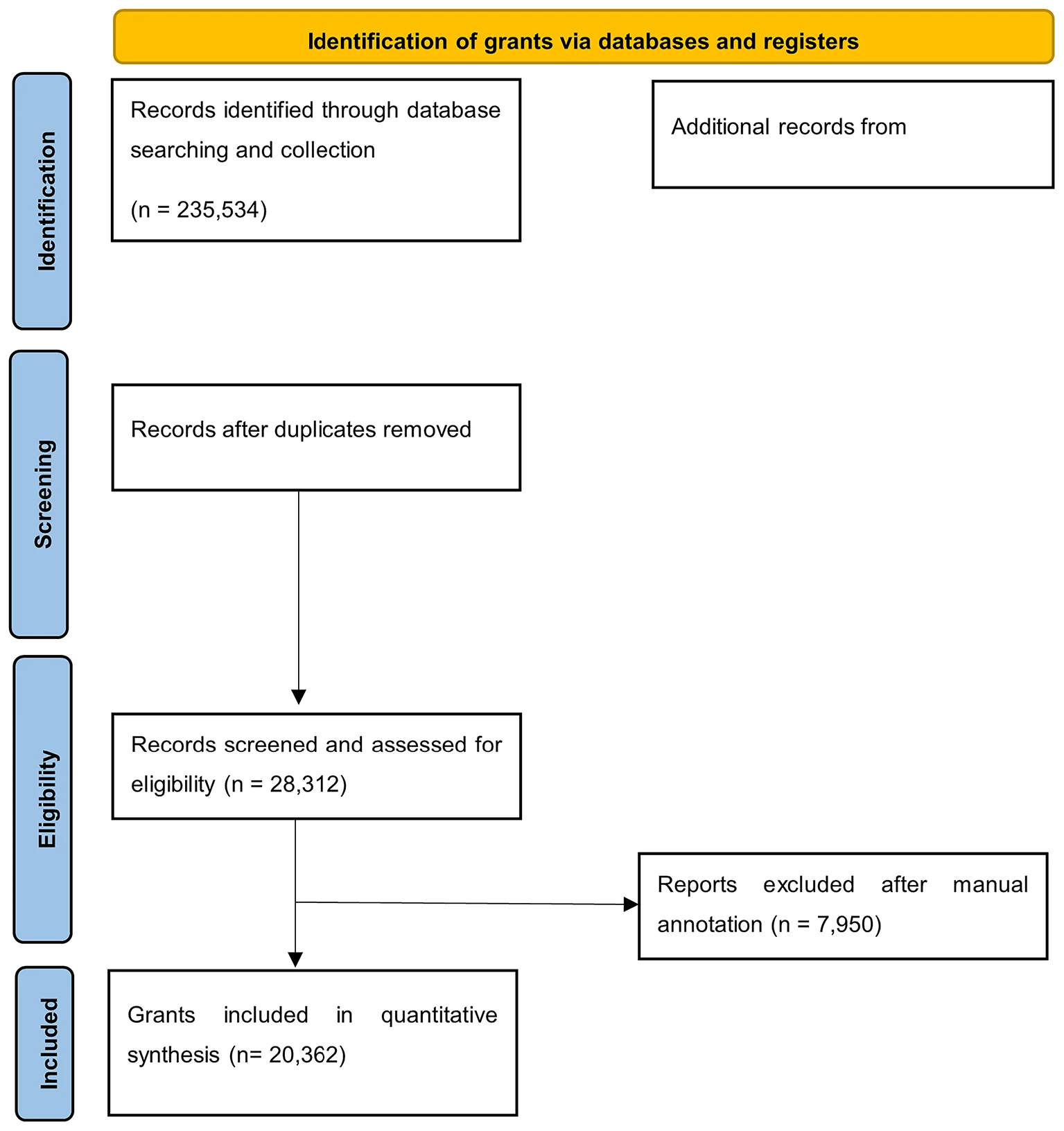
PRISMA flow diagram. Summary of the Pandemic PACT grant selection process.

### Data completeness

As of 1 March 2025, a total of 20,362 grants were captured in the Pandemic PACT Tracker. Funding amount information was available for 68.7% (n = 13,994/20,362) of these grants. Additionally, the grant abstract was available for 70.3% (n = 14,322/20,362), and the grant title for 99.8% (n = 20,321/20,362) of the grants. Information on the funder-assigned grant identifier, research institution, and study subject were available for 63.6% (n = 12,950/20,362), 87.8% (n = 17,879/20,362), and 90.0% (n = 18,319/20,362) of the grants, respectively. Table 1 summarises the availability and sources of data for key Pandemic PACT fields.

**Table 1.** List of key Pandemic PACT variables with corresponding data completeness and data source.

Variable name	Completeness (n)	Completeness (%)	Data source
Funder-assigned grant ID	12,950	63.6%	Funder website/provided data
Grant title	20,321	99.8%	Funder website/provided data
Grant abstract	14,322	70.3%	Funder website/provided data
Start year	20,362	100.0%	Funder website/provided data
End year	10,151	49.9%	Funder website/provided data
Financial commitment	13,994	68.7%	Funder website/provided data
Funder organisation	20,362	100.0%	Funder website/provided data
Research institution name	17,879	87.8%	Funder website/provided data
Research location country	19,730	96.9%	Funder website/provided data
Study subject	18,319	90.0%	Human annotation
Study type	13,898	68.3%	Human annotation

Summary of the availability and sources of data for key Pandemic PACT fields. Data cover the period from 2020 to 1 March 2025.

### Total known financial commitments

Of the known financial commitments, the grant amounts awarded ranged from $111 to $416.3 million, with a median value of $209,657 and a combined total investment of at least $14.4 billion invested by 326 funding entities spread across 58 different countries.

### Funding organisations

Most grants (30.6%, n = 6,234/20,362) were awarded by US-based funders, with a total known financial commitment of nearly $5.1 billion, representing 35.4% of the total known funding commitments. Of these, the National Institutes of Health (NIH) funded $3.5 billion across 3,181 grants, while the National Science Foundation (NSF) and the Gates Foundation awarded 1,499 and 651 grants, financing $426.5 million and $1 billion, respectively. Figure 2 illustrates the global distribution of total known financial commitments by funder location.

**Figure 2.**
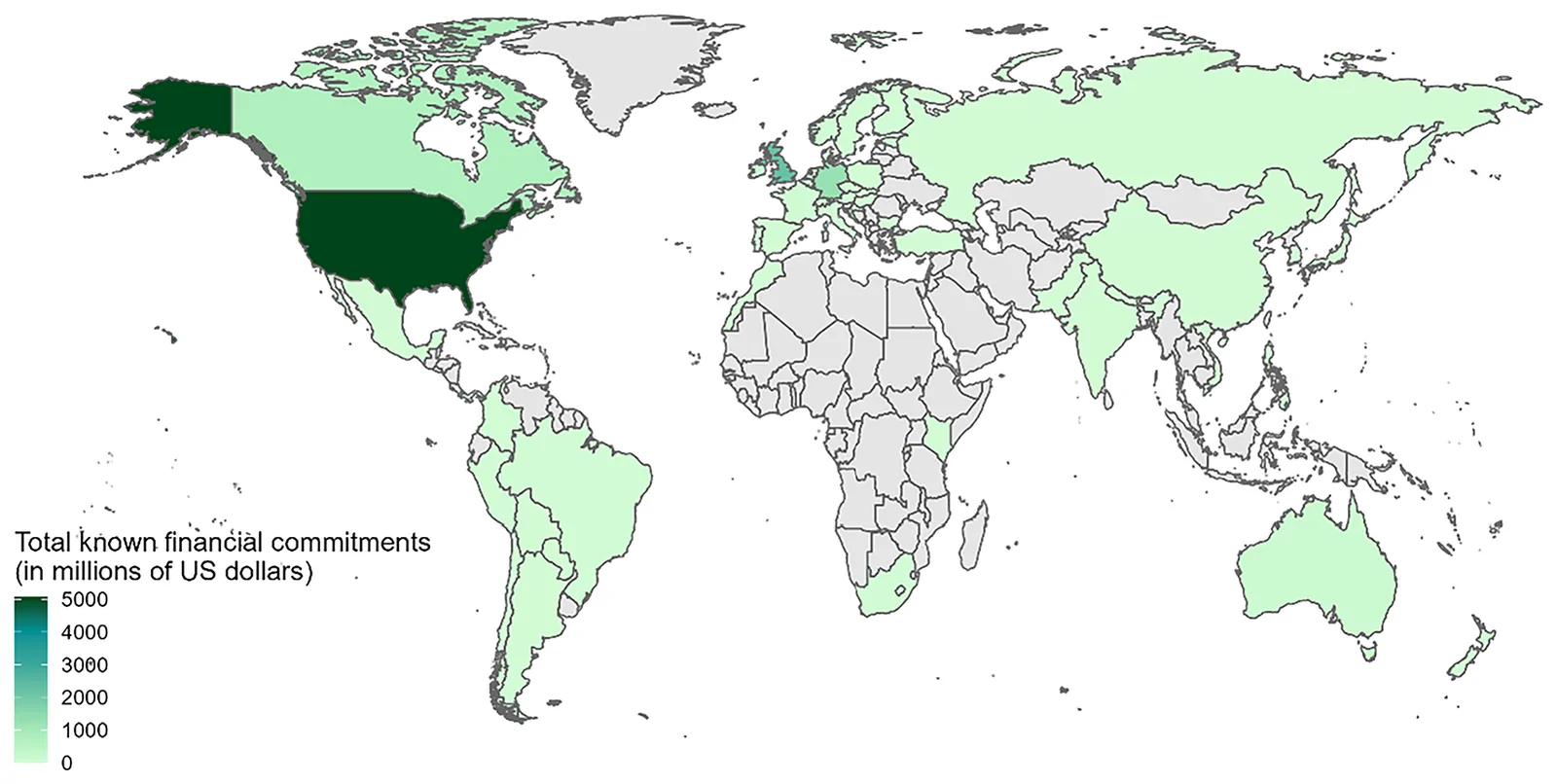
Global distribution of total known financial commitments by funder location. Funding amounts are included only when they have been published by the funder. Data cover the period from 2020 to 1 March 2025. Funding amounts are presented as absolute values. Financial commitment information is available for 68.7% of the grants in the Pandemic PACT database (n = 13,994/20,362). Although grants may be awarded by multiple funding organisations from different countries, this is uncommon, with only 0.7% of grants with known financial commitments involving cross-country funding (n = 100/13,994). In such cases, there is a possibility of double counting, as the same financial commitment may be recorded under multiple countries. In Extended Data 4, we illustrate the global distribution of total grants by funder location available in the Pandemic PACT database.

UK-based institutions funded the second-highest known financial commitment, totalling $2.2 billion, or 15.3% of all known funding commitments. Among UK-based funders, the UK Research and Innovation (UKRI) awarded $1.3 billion across 1,375 grants, while the Wellcome Trust awarded $519.7 million across 285 grants. Figure 3 provides a detailed breakdown of the total number of grants and financial commitments for the funding organisations with the highest levels of known funding, according to the data.

**Figure 3.**
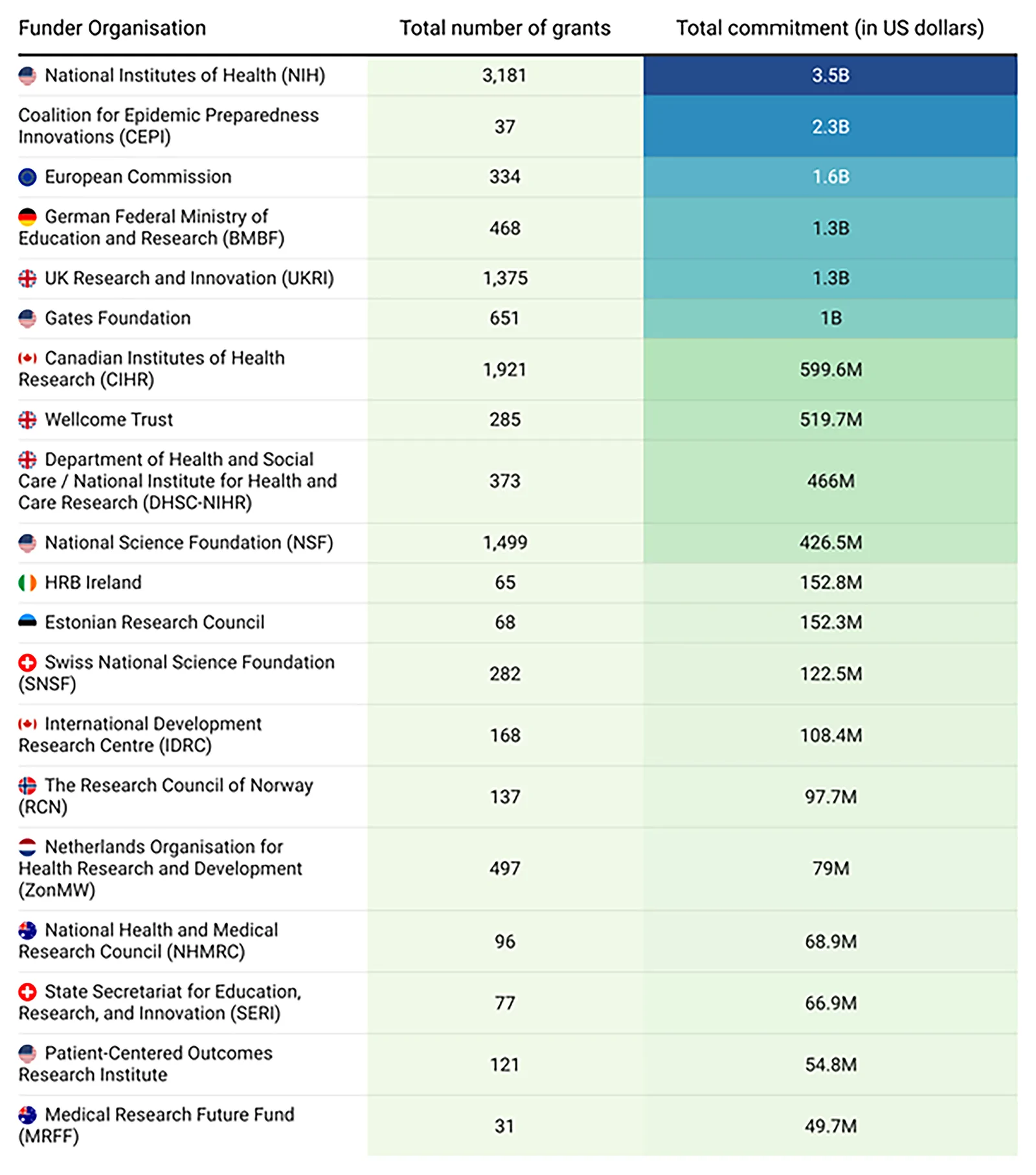
Total number of grants and known financial commitments by funding organisations, sorted by total known financial commitments. The figure shows the twenty funding organisations with the highest funding amounts, according to the Pandemic PACT data. Data cover the period from 2020 to 1 March 2025. Flags next to funder names represent the location where the funder is based. No flags are used for multinational organisations. Many research funding organisations operate independently. However, funding mechanisms, sources, and flows vary across funders and can be interconnected. The analyses in this paper do not account for relationships between funding organisations unless they are explicitly listed as co-funders. As a result, contributions from some funders to other entities or funding schemes (when not explicitly stated) were not included in the total funding calculations, potentially leading to an underestimation of their contributions in some cases. Funding amounts are presented as absolute values.

Among all 326 funders, the NIH in the US ranks first with a total known financial commitment of $3.5 billion distributed across 3,181 grants, averaging $1.1 million per grant, followed by the Coalition for Epidemic Preparedness Innovations (CEPI), which contributed $2.3 billion across 37 grants, with an average of $62.2 million per grant. It is important to note that CEPI receives its funds from some of the other funders in this figure. Extended Data 1 presents a detailed breakdown of total number of grants and financial commitments, along with the percentage of grants with funding amount information available, for each funding organisation.

### Research institutions

Research funding was allocated to research institutions spread across 129 countries. Among these, 26.9% (n = 5,468/20,362) of the grants available in the Pandemic PACT Tracker were awarded to institutions in the US, amounting to $4.7 billion and representing 32.4% of the total known funding commitments. Research institutions in the UK received $2.3 billion, allocated across 1,998 grants. The BioNTech SE, based in Germany, secured the largest known financial investment, totalling $421 million allocated across three grants aimed at supporting research on COVID-19. Ranking second, third, and fourth in total known funding were Novavax in the US, Clover Biopharmaceuticals in China, the University of Oxford in the UK. These organisations received total financial commitments of $418 million, $397 million, and $384 million, respectively, distributed across multiple grants covering a wide range of research areas. Figure 4 illustrates the global distribution of total known financial commitments by research institution location.

**Figure 4.**
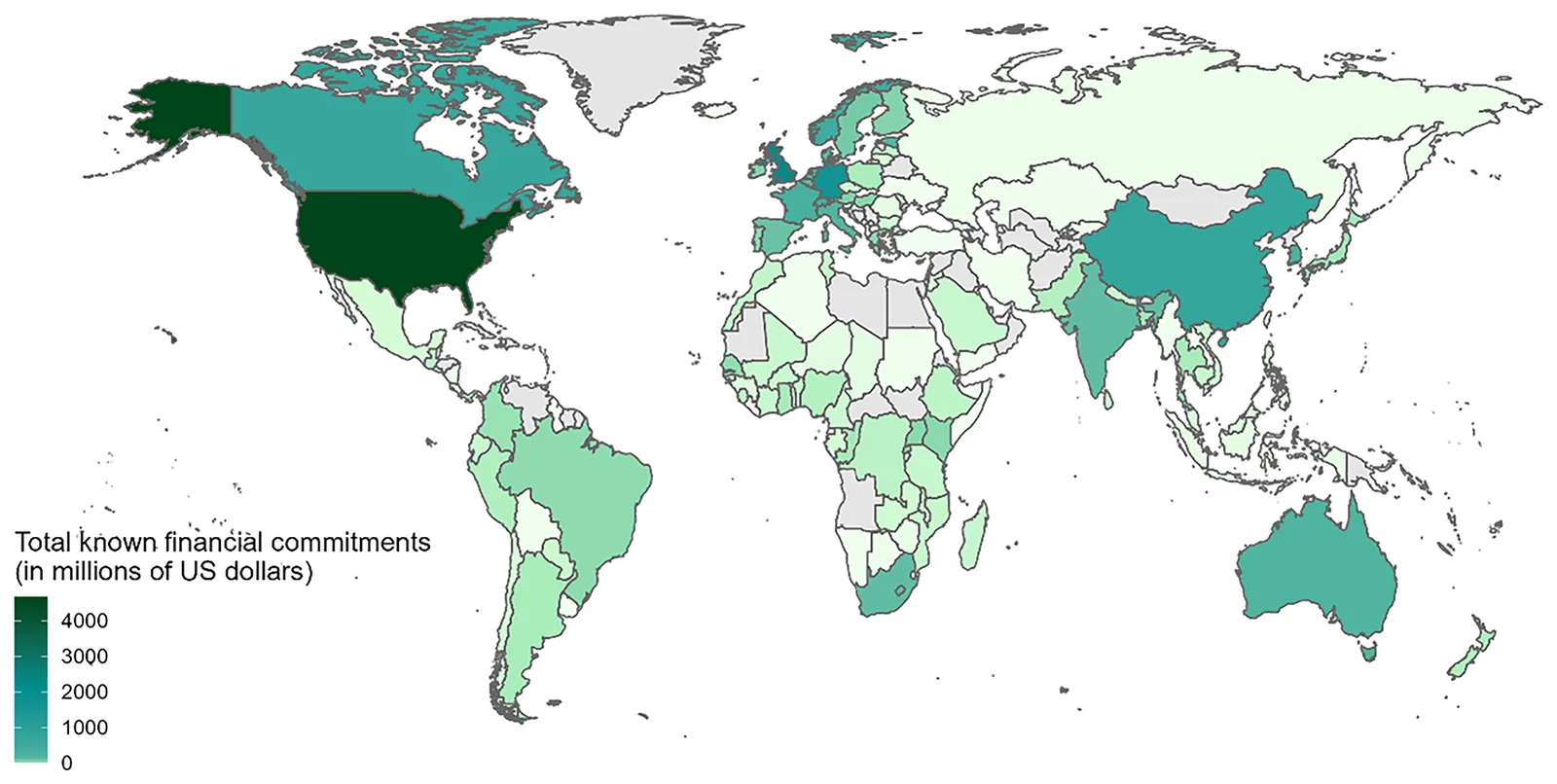
Global distribution of total known financial commitments by research institution location. Funding amounts are included only when they have been published by the funder. Funding amounts are presented as absolute values. Data cover the period from 2020 to 1 March 2025. Financial commitment information is available for 68.7% of the grants in the Pandemic PACT database (n = 13,994/18,470). Although grants may be awarded to multiple research institutions from different countries, this is uncommon, with only 0.2% of grants with known financial commitments involving cross-country awarding (n = 29/13,994). In such cases, there is a possibility of double counting, as the same financial commitment may be recorded under multiple countries. In Extended Data 4, we illustrate the global distribution of total grants by research institution location available in the Pandemic PACT database.

The distribution of funded research institutions closely reflects the patterns observed in funding sources. The majority of funding sources originate from organisations exclusively based in high-income countries (HICs) [1], accounting for 87.7% (n = 17,847/20,362) of the total grants and $14.3 billion in investments, which represent 99.3% of the total financial commitments. Relatedly, only 11.5% (n = 2,336/20,362) of the grants were allocated to grants involving at least one research institution based in a LMIC. These investments amounted to $1.3 billion, representing 9% of the total known financial commitments. Among LMICs, the largest funding amount was directed to research institutions in China, which received $740.2 million across 62 grants. Notably, the majority of these grants (80.6%, n = 50/62) were funded by non-Chinese organisations, with nearly two-thirds (64.5%; n = 40/62) of the total grants supported by either Canadian (22 grants) or US-based (18 grants) funders. However, it is important to acknowledge that the Pandemic PACT database currently has limited representation of funding data from Chinese institutions, which may influence the completeness of these estimates. Indian research institutions ranked as the second-largest recipients, securing at least $124.4 million in funding across 296 grants. More than two-thirds of these grants (67.9%, n = 201/296) were funded by funders based in India. An additional 10.1% (n = 30/296) were co-funded by both Indian and non-Indian funders, while 22% (n = 65/296) were exclusively funded by external sources.

Figure 5 provides a detailed breakdown of the total number of grants and financial commitments for research led by at least one institution based in an LMIC, highlighting the countries with the highest levels of known funding, according to the data, and Figure 6 depicts the flow of financial commitments, contrasting grants led by institutions in LMICs with those entirely led by institutions in HICs. The figure highlights the disparity in funding allocation between these groups. For instance, US-based funder organisations allocated $2.2 billion in total funding, but only 3.4%—equivalent to $75.2 million—was directed toward grants led by at least one institution in an LMIC, reflecting the common requirement of many UK-funded programmes for lead institutions to be UK-based. Similarly, US-based funders invested $5.1 billion in total funding, of which just 4.5%, or $228.1 million, was awarded to grants led by at least one LMIC-based institution.

**Figure 5.**
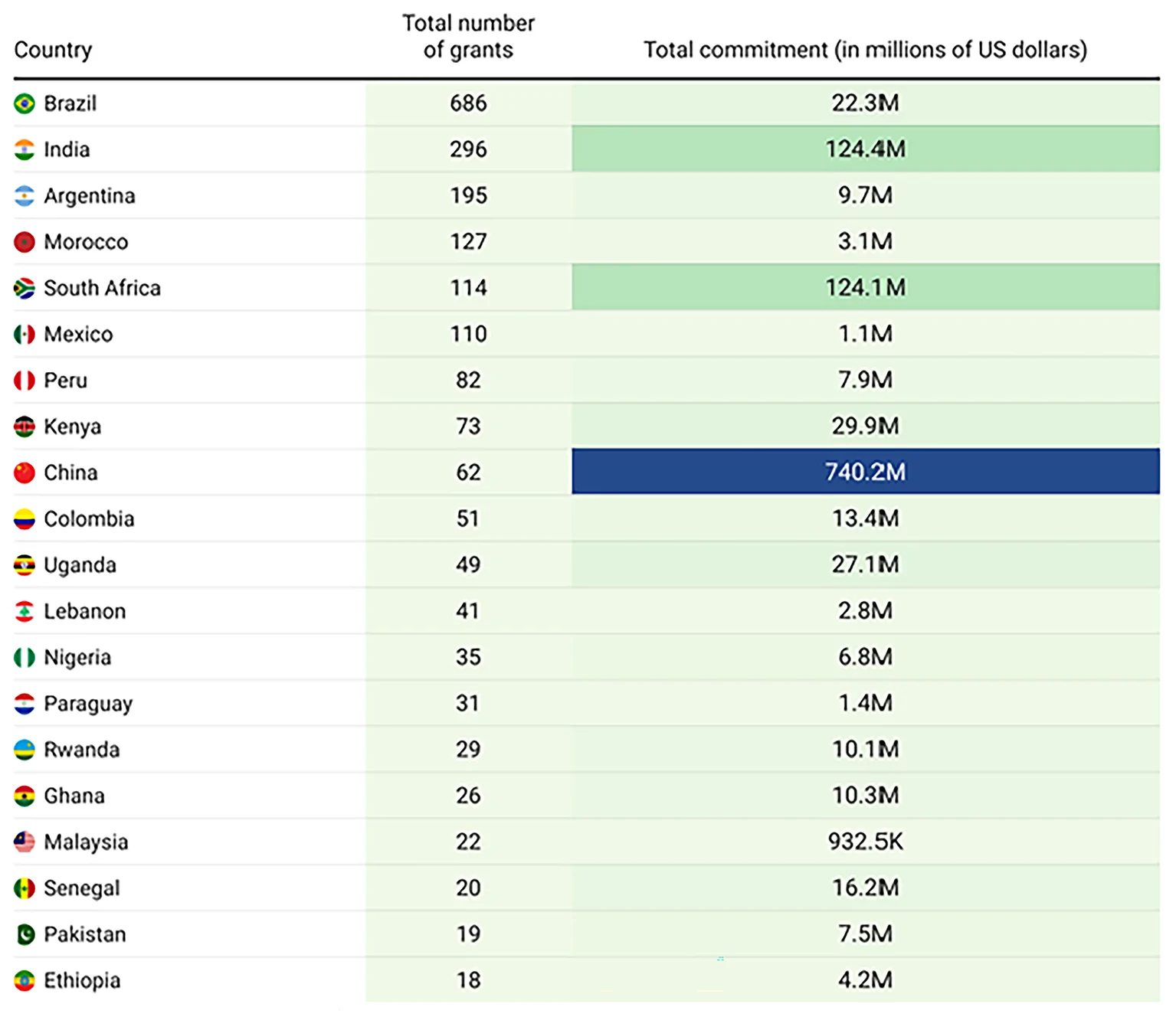
Total number of grants and known financial commitments by research institution countries located in LMICs, sorted by total number of grants. The figure shows the twenty research institution countries located in LMICs with the highest funding amounts, according to the Pandemic PACT data. Data cover the period from 2020 to 1 March 2025. Grants may be awarded to multiple research institutions from different countries, resulting in a total count by country exceeding the overall number of grants and known financial commitments available in Pandemic PACT. Funding amounts are presented as absolute values.

**Figure 6.**
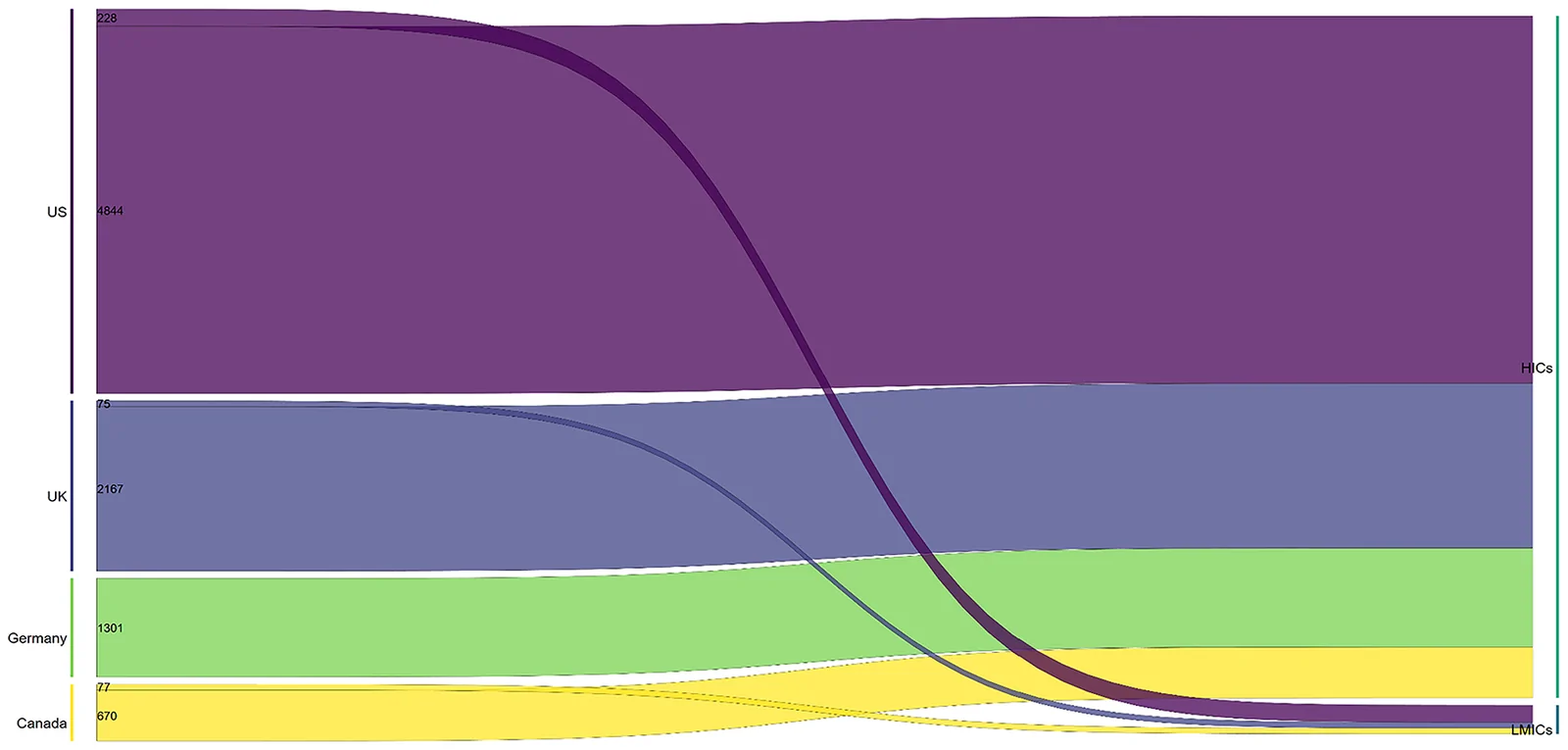
Distribution of known financial commitments (in millions of US dollars) to research institutions in LMICs and HICs by funding organisation country. The figure shows the four countries where funder organisations with the largest financial commitments are based, according to the dataset: UK, US, Germany, and Canada. Grants awarded by at least one these countries to grants led by at least one institution based in a LMIC totalled approximately $371.4 million. This accounts for 28.6% of the total known financial commitments to LMIC-led grants in the dataset, which amount to $1.3 billion. These grants represent 23.2% (n = 543/2,366) of grants awarded to research involving at least one LMIC-based institution. Funding amounts are included only when reported by the funder. Financial commitments information is available for 46.5% of LMIC-led grants in the Pandemic PACT database (n = 1,087/2,366). Although grants may be awarded by multiple funding organisations from different countries, this is uncommon, with only 2.1% of grants led by at least one institution based in a LMIC with known financial commitments involving cross-country funding (n = 23/1,087). In such cases, there is a possibility of double counting, as the same financial commitment may be recorded under multiple countries. Funding amounts are presented as absolute values. Data cover the period from 2020 to 1 March 2025.

According to the data, South Africa stands out as the primary LMIC recipient of financial commitments from UK-based funders, receiving nearly $20 million across 17 grants. Of these, seven were funded by the Wellcome Trust, three by the UKRI, two by the Department of Health and Social Care/National Institute for Health and Care Research (DHSC-NIHR), two co-funded by both DHSC-NIHR and UKRI, two co-funded by DHSC-NIHR and the Royal Society of Tropical Medicine and Hygiene (RSTMH), and one by Cambridge Africa.

US-based funders similarly directed a significant share of their LMIC investments to South Africa, which received approximately $85 million across 47 grants. Of these, 26 grants, amounting to approximately $50 million, were awarded to the Wits Health Consortium, with all funding provided by the Gates Foundation. Other major LMIC recipients of US-based funding spanned Asia, Africa, and Latin America. India received approximately $45 million across 51 grants, followed by Uganda with $15 million across 8 grants, and Kenya with $12 million spread over 12 grants. In Brazil, $11 million was allocated through 8 grants, while China received $10 million across 18 grants. Figure 7 illustrates the distribution of known financial commitments to grants led by institutions in LMICs from UK- and US-based funders, whose countries contributed the largest share of high-income country investments in LMICs.

**Figure 7.**
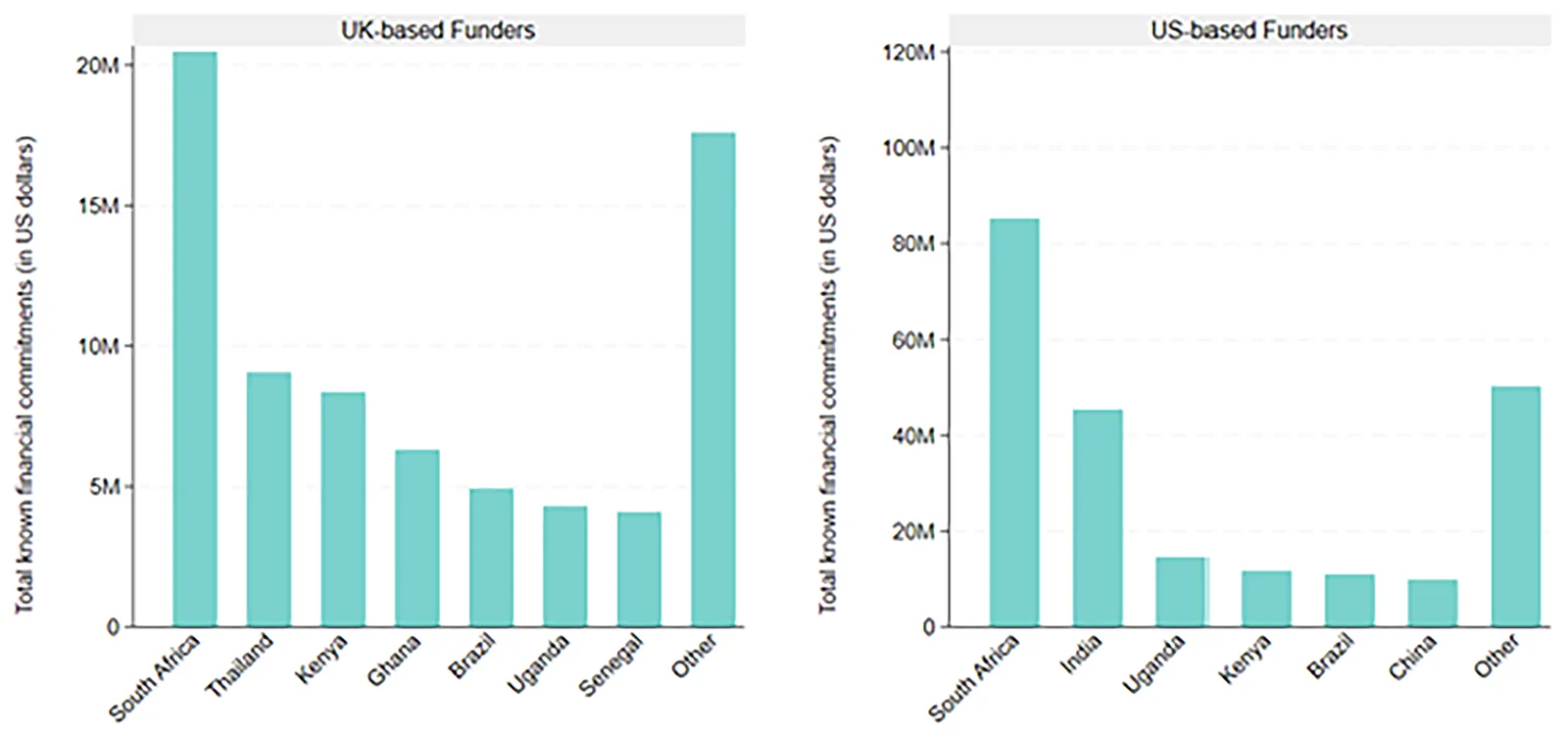
Distribution of known financial commitments (in US dollars) from UK- and US-Based Funders to Grants Led by Institutions in LMICs. Funding amounts are included only where they have been publicly reported by the funder. Funding amounts are presented as absolute values. Data cover the period from 2020 to 1 March 2025. Financial commitment information is available for 61.6% of the grants led by at least one institution based in a LMIC and funded by UK-based organisations (n = 68/177), and for 87.7% of those funded by US-based organisations (n = 193/220). Grants may be awarded to multiple research institutions from different countries, resulting in a total count by country exceeding the overall number of grants and known financial commitments available in Pandemic PACT. Other LMICs funded by UK-based organisations include: Bangladesh, Benin, Bolivia, Brazil, Cambodia, China, Colombia, Congo (DRC), Ecuador, Ethiopia, Gambia, Ghana, India, Indonesia, Jamaica, Kenya, Lebanon, Madagascar, Malawi, Malaysia, Mali, Mexico, Nepal, Namibia, Nigeria, Pakistan, Palestine, Peru, Philippines, Rwanda, Senegal, Sierra Leone, Somalia, South Africa, Sri Lanka, Tanzania, Thailand, Uganda, Zambia, and Zimbabwe. Other LMICs funded by US-based organisations include: Argentina, Bangladesh, Botswana, Cambodia, Cameroon, Colombia, Congo, Democratic Republic of the Congo, El Salvador, Ethiopia, Ghana, Indonesia, Kazakhstan, Madagascar, Malawi, Mali, Mexico, Mozambique, Nepal, Nicaragua, Nigeria, Pakistan, Peru, Rwanda, Senegal, Sierra Leone, Thailand, Tunisia, Turkey, and Zambia.

Funding from Canadian institutions to LMIC-based institutions was also meaningful. Among Canadian funders, Kenya was the largest recipient of LMIC-directed funding, receiving approximately $8 million across 15 grants. Other key recipients included Uganda, with $7 million across 8 grants; Senegal, which secured $6 million through 11 grants; South Africa, with $5 million across 8 grants, and Peru, which was awarded $5 million through 3 grants. In comparison, Danish funders supported a smaller number of projects involving LMIC-based institutions. A total of thirteen grants were identified, with known financial commitment information available for two. These two grants, each valued at $800,000, were awarded to institutions in Jordan and Tanzania, respectively. Both grants were funded by the Novo Nordisk Foundation.

### Global funding for epidemic-prone infectious disease research

Since some research grants may span multiple diseases (6.6% of grants with known financial commitments; n = 927/13,994), it is challenging to determine the exact portion of the award directed to each disease. To account for this, we present both potential and minimum funding estimates. The potential values represent the total amount a specific disease could have received if it had been allocated the entirety of the awards, while the minimum values reflect funding for grants exclusively focused on that disease. An analysis of the total known funding allocated to epidemic-prone infectious diseases revealed that grants focused on COVID-19 were particularly prominent. COVID-19 research received at least $11.1 billion, with a potential total financial commitment nearing $11.8 billion distributed across 18,119 grants. This substantial investment reflects the global impact of the pandemic and the concentrated efforts to address it. Accordingly, the potential total known financial commitment to COVID-19 research, which includes grants exclusively focused on COVID-19 as well as those addressing multiple diseases that include COVID-19, accounts for approximately 81.9% of the total grant commitments available in the Pandemic PACT database. Figure 8 illustrates the distribution of total known financial commitments across diseases, highlighting both the minimum and maximum amount. The distribution is highly skewed, with COVID-19 dominating the funding landscape. To put into perspective, the disease with the second-largest known financial commitment is the concept ‘Disease X’, which has received at least $415.1 million, with potential commitments approaching $866.4million. Grants addressing the concept of “Disease X” broadly support research on unknown or emerging pathogens with pandemic potential. They generally focus on strengthening pandemic preparedness and response, aiming to generate the evidence, tools, and networks that can be rapidly mobilised when a previously uncharacterised pathogen emerges.

**Figure 8.**
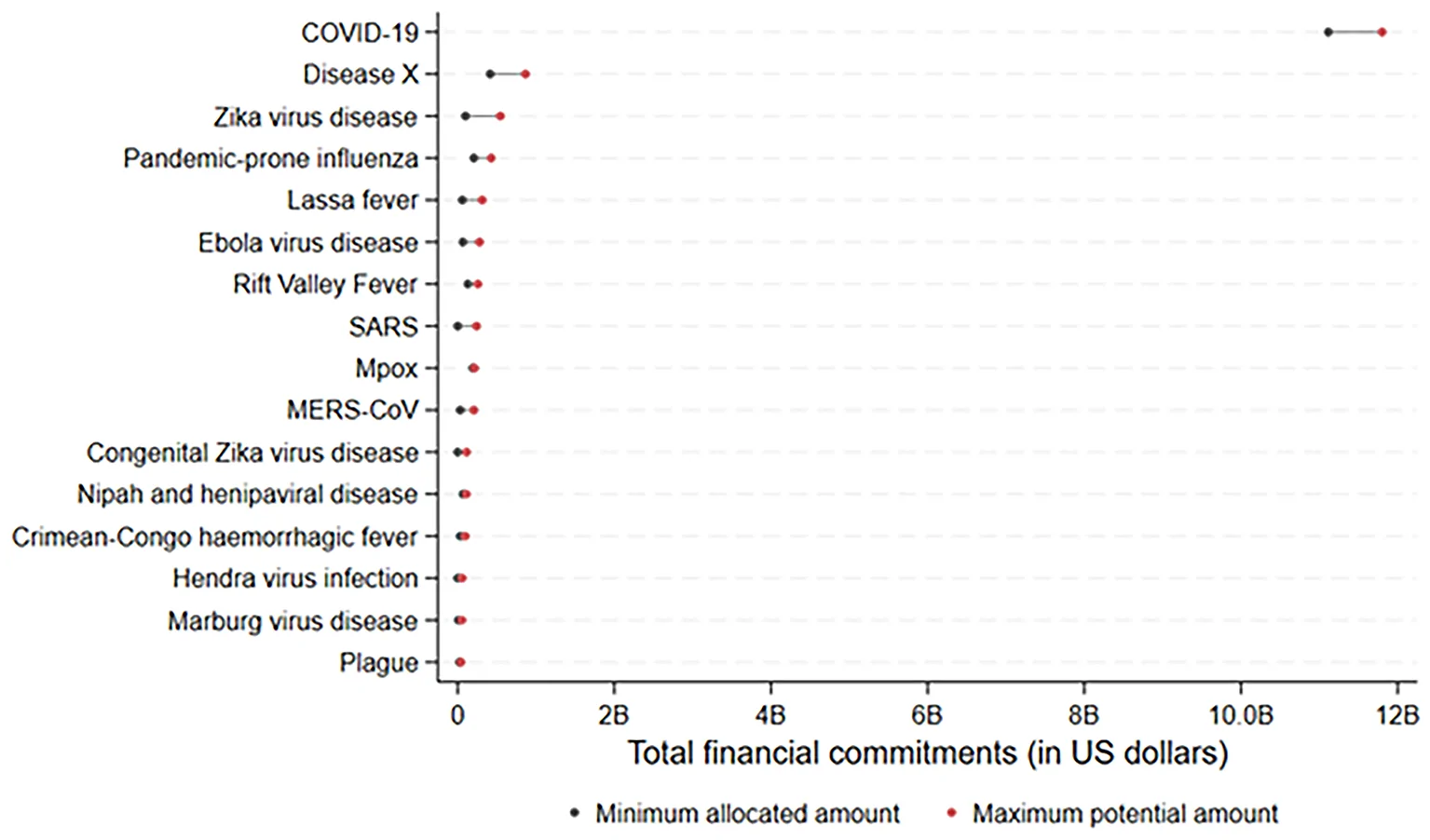
Funding distribution across Pandemic PACT list of diseases. Grey dots represent the minimum known funding amount allocated to projects entirely focused on a specific disease. The maximum values indicate the potential maximum total funding amount that a particular disease could have received. The red dots represent the maximum total funding that could have been allocated to a disease if all awards for projects spanning multiple diseases had been directed to that disease. Data cover the period from 2020 to 1 March 2025.

When analysing the total known funding allocated across diseases, excluding grants exclusively focused on COVID-19, the concept of ‘Disease X’ received the highest amount of financial commitments, according to the dataset. Following this, research on Zika virus disease, Pandemic-prone influenza, and Lassa fever ranked second, third, and fourth, with potential commitments of $544.9 million, $428.2 million, and $313 million, respectively. Figure 9 illustrates the distribution of total known financial commitments across diseases, excluding COVID-19-focused grants. Notably, plague and Marburg virus disease received the lowest levels of financial commitments, with potential total investments of approximately $42.8 million and $51.9 million, respectively.

**Figure 9.**
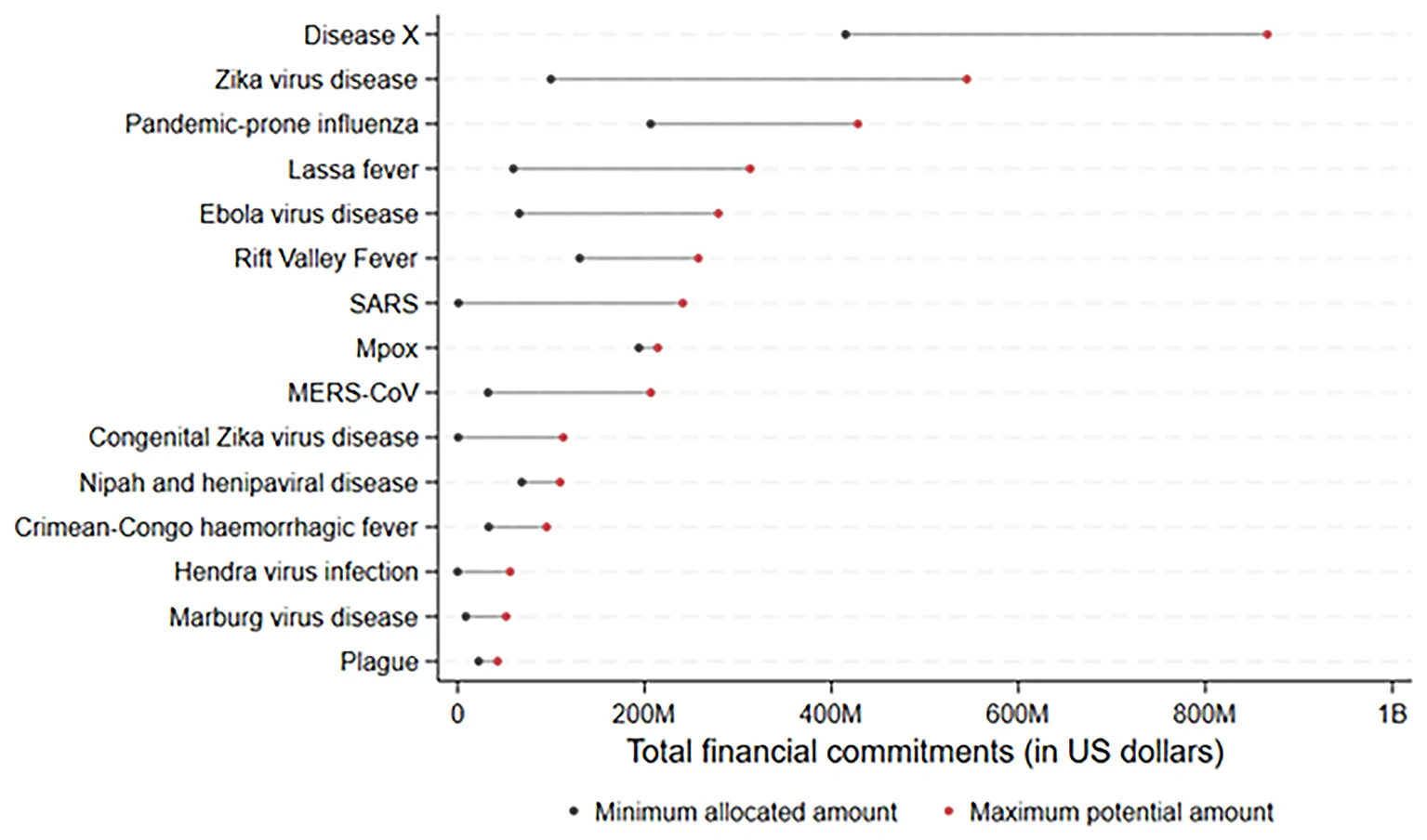
Funding distribution across Pandemic PACT list of diseases, excluding grants exclusively focused on COVID-19. Grey dots represent the minimum known funding amount allocated to projects entirely focused on a specific disease. The maximum values indicate the potential maximum total funding amount that a particular disease could have received. The red dots represent the maximum total funding that could have been allocated to a disease if all awards for projects spanning multiple diseases had been directed to that disease. Data cover the period from 2020 to 1 March 2025.

By presenting potential and minimum funding estimates for research on pandemic-prone diseases, Figure 9 provides additional insight into the extent to which each disease is studied independently versus in combination with others. For example, while the concept of ‘Disease X’ has received the highest potential financial commitments, there is a substantial gap between its minimum and potential funding totals. Specifically, among the 350 grants that include the concept of ‘Disease X’, fewer than half (42.9%; n = 150/350) are exclusively focused on it, indicating that most grants study the concept of ‘Disease X’ with other diseases. In contrast, research on Mpox is predominantly conducted in isolation: 77.9% (n = 95/122) of grants dedicated to Mpox focus solely on this disease. Of the $214.2 million in potential funding for Mpox research, $194 million has been allocated to grants studying Mpox alone. Hendra virus infection is the only disease for which no known financial commitments have been allocated exclusively to it. The entirety of its $56.2 million in potential funding has been directed toward grants that also include other diseases.

### Annual trends in global funding (2020–2024)

Pandemic PACT also tracks the timing of research funding, including the start dates of grants or the publication years of awards. Although it is challenging to precisely determine the yearly distribution of funding within a grant’s duration, understanding the start dates of grants provides valuable insights into the timing of investment decisions and the alignment of funding priorities with emerging public health needs. Figure 10 illustrates the evolution of total known financial commitments across diseases, highlighting trends in financial commitments for diseases with the highest potential known investments from 2020 to 2024, revealing distinct trajectories.

**Figure 10.**
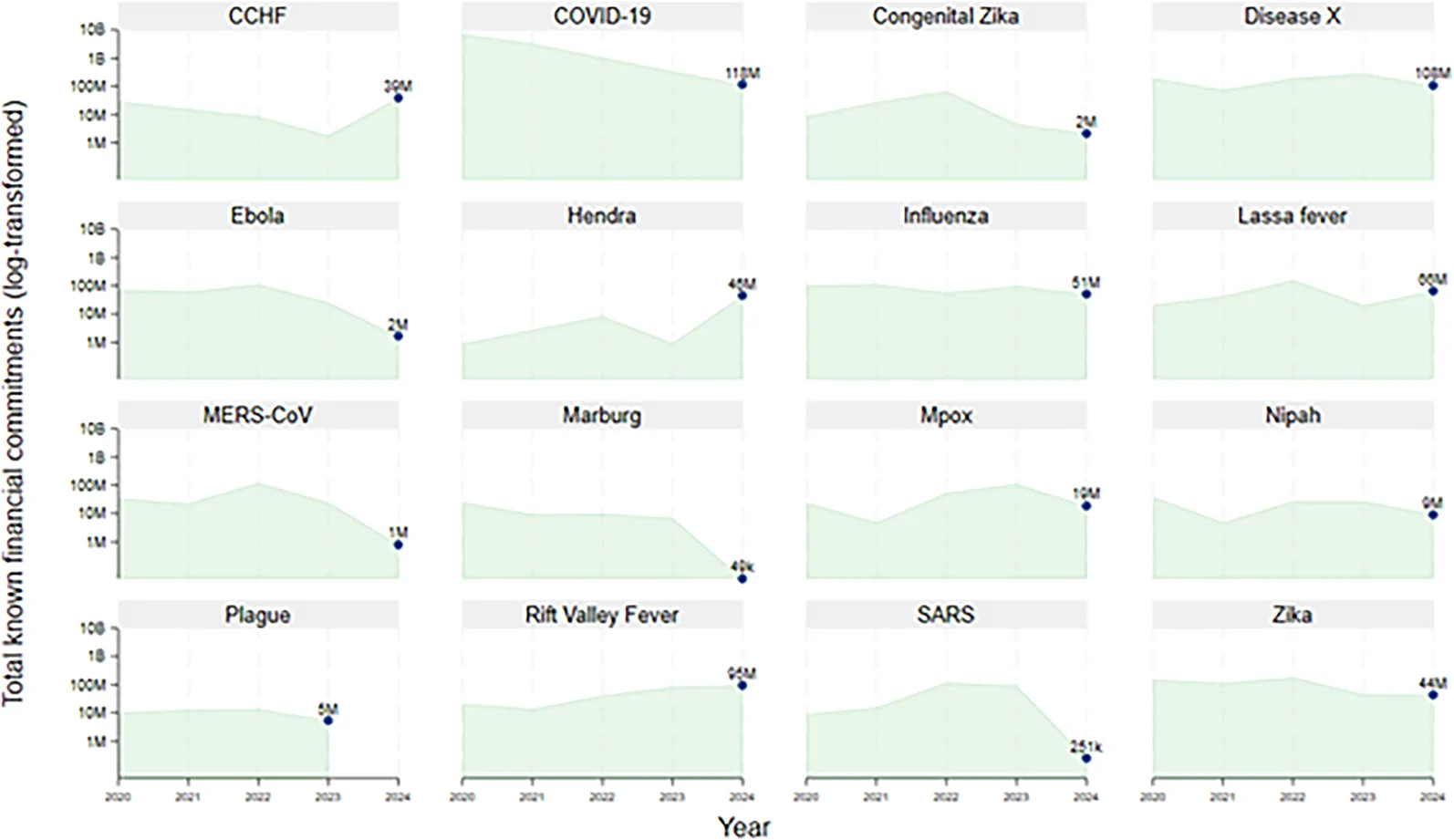
Total know financial commitments across diseases over time. The figure illustrates the total maximum potential investment in each epidemic-prone disease listed in Pandemic PACT. Although grants may be awarded to research grants spanning more than one disease, this is uncommon, with only 6.6% of grants with known financial commitments involving multiple diseases grants (n = 927/13,994). In such cases, there is a possibility of double counting, as the same financial commitment may be recorded under multiple diseases. Due to the highly skewed distribution of funding amounts, where a small number of diseases (particularly COVID-19) received disproportionately large investments, total known financial commitments were log-transformed. Data cover the period from 2020 to 1 March 2025.

For instance, funding for the concept of ‘Disease X’ exhibited clear fluctuations. After a potential known investment of $197.1 million in 2020, new commitments dropped to $75.2 million in 2021, then increased to $197.2 million in 2022 and $288.5 million in 2023, before rebounding to nearly $108.4 million in 2024. Pandemic-prone influenza followed a different pattern, with relatively high new funding of approximately $110 million in 2020 and 2021, a decline to $56.5 million in 2022, and a recovery to $100 million in 2023, before rebounding again to nearly $50 million in 2024. In contrast, funding for Mpox began at relatively low levels, with $24.2 million in 2020 and $4.9 million in 2021, but grew steadily over time, reaching $112.2 million in 2023. Meanwhile, Zika reached its peak funding in 2022 with $185.7 million in potential commitments, which represents the highest annual investment among grants for non-COVID-19 and non-Disease X research. However, funding for Zika declined over the next years.

These shifting funding trajectories reflect, at least in part, the evolving landscape of infectious disease outbreaks (which provide opportunities for types of research, such as assessment of medical countermeasures, that can only be conducted during an outbreak) and their associated public health burden, although the relationship between funding allocation and disease burden is not always direct. The rapid increase in Mpox funding from 2021 onwards coincides with the escalation of the global Mpox outbreak, which was declared a Public Health Emergency of International Concern (PHEIC) by WHO in 2022. ^22^ Similarly, the sustained relatively high investment in the concept of ‘Disease X’ throughout the period reflects the prominent position that broad pandemic preparedness has occupied on the global health agenda since COVID-19. The steady increase in funding for Rift Valley fever is also noteworthy. Following its classification by WHO in 2024 as a high-priority pathogen with PHEIC potential, ^23^ and the subsequent outbreak in Senegal in 2025, this pattern may suggest that funding allocations can, in some cases, align with emerging epidemiological risks prior to formal emergency declarations.

In contrast, other diseases highlight the limitations in rapidity of reactive funding models. For example, Marburg virus disease received its lowest level of funding in 2024, despite Rwanda declaring its first-ever outbreak in September of that year, an event assessed by WHO as posing very high national risk. ^24^ A similar pattern is observed for Ebola. Despite ongoing outbreaks in the Democratic Republic of the Congo and Uganda, funding levels did not show a corresponding increase in the dataset. For both diseases, research investments responding to these outbreaks are expected to be reflected in subsequent updates to this living review.

### Global funding by epidemic-prone infectious diseases and funding organisations

A total of 48 funding organisations announced financial commitments to research on epidemic-prone infectious diseases, extending their focus beyond COVID-19. During this period, the NIH reported the highest known financial commitments, amounting to $1.5 billion distributed across 1,627 grants. The European Commission and CEPI followed, with commitments of $670.3 million across 123 grants and $322.9 million across 17 grants, respectively. Figure 11 provides a detailed breakdown of the total number of grants and known financial commitments for the funding organisations with the highest levels of known funding, according to the data, supporting research on epidemic-prone infectious diseases during the entire period. Notably, all organisations with the largest funding commitments are based in HICs.

**Figure 11.**
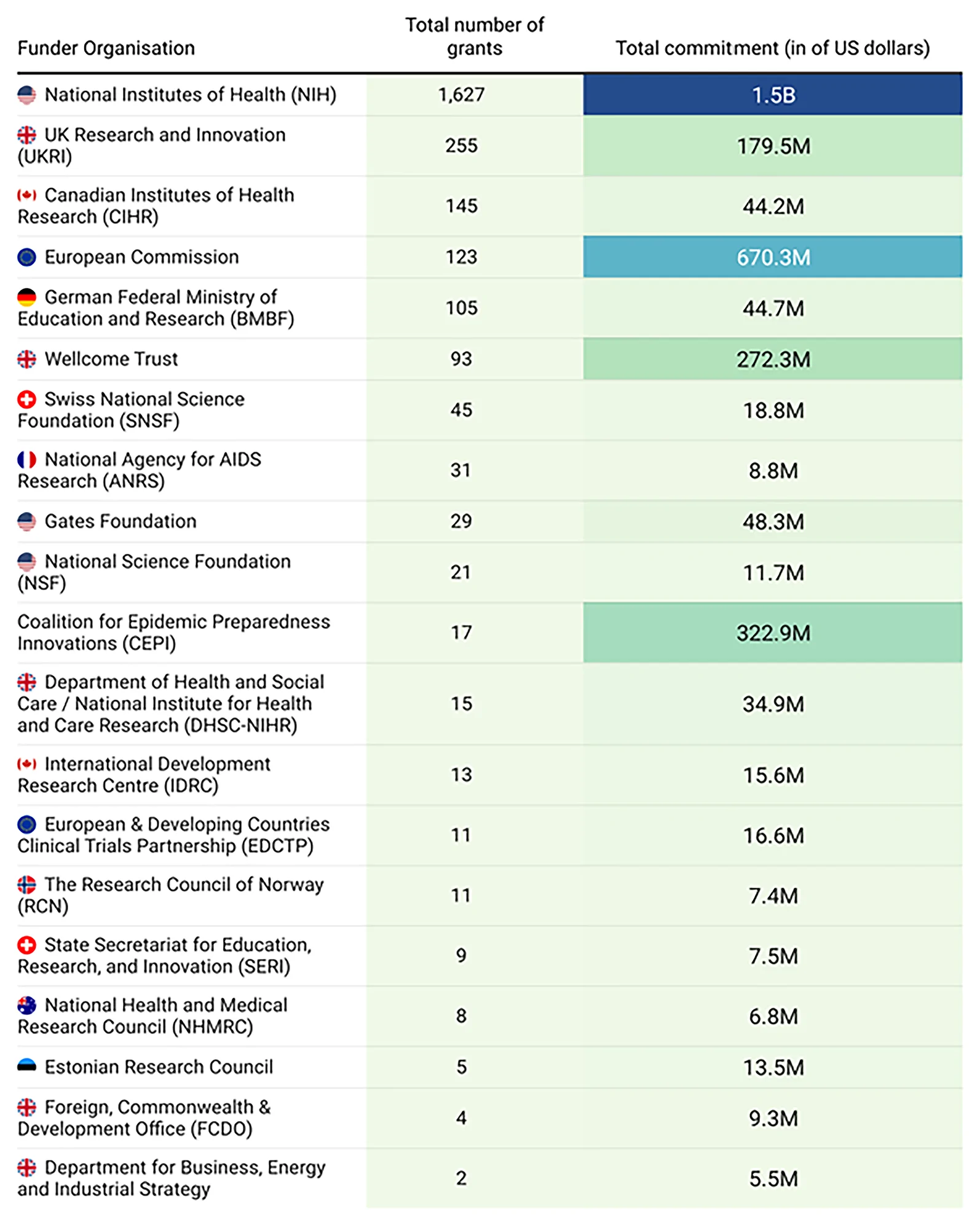
Total number of grants and total known financial commitments by funding organisations supporting research on Pandemic PACT diseases (excluding COVID-19), sorted by total number of grants. The figure shows the twenty funding organisations with the highest funding amounts, according to the Pandemic PACT data. Data cover the period from 2020 to 1 March 2025. Flags next to funder names represent the country where the funder is based. No flags are used for multinational organisations. Many research funding organisations operate independently. However, funding mechanisms, sources, and flows vary across funders and can be interconnected. The analyses in this paper do not account for relationships between funding organisations unless they are explicitly listed as co-funders. As a result, contributions from some funders to other entities or funding schemes (when not explicitly stated) were not included in the total funding calculations, potentially leading to an underestimation of their contributions in some cases.

### Global funding by epidemic-prone infectious diseases and research areas

Apart from grants solely focussed on COVID-19, the distribution of funding across research areas reveals that the largest portion was allocated to vaccine research, development, and implementation, with a potential total financial commitment of $1.2 billion distributed across 466 grants to this area. This was closely followed by funding for research on pathogen morphology, transmission dynamics and diagnostics, which accounted for a potential total financial commitment of $934.9 million distributed across 1,287 grants. In contrast, the least-funded research area, according to the data, was on research to inform ethical issues, with $13.1 million in potential financial commitments distributed across 26 grants. It is important to note that differences in the average grant amount across research areas, particularly the higher costs associated with vaccine research compared to ethics research, may contribute to these disparities. Figure 12 illustrates the total known financial commitments allocated to research areas across diseases, highlighting the variations in investment focus.

**Figure 12.**
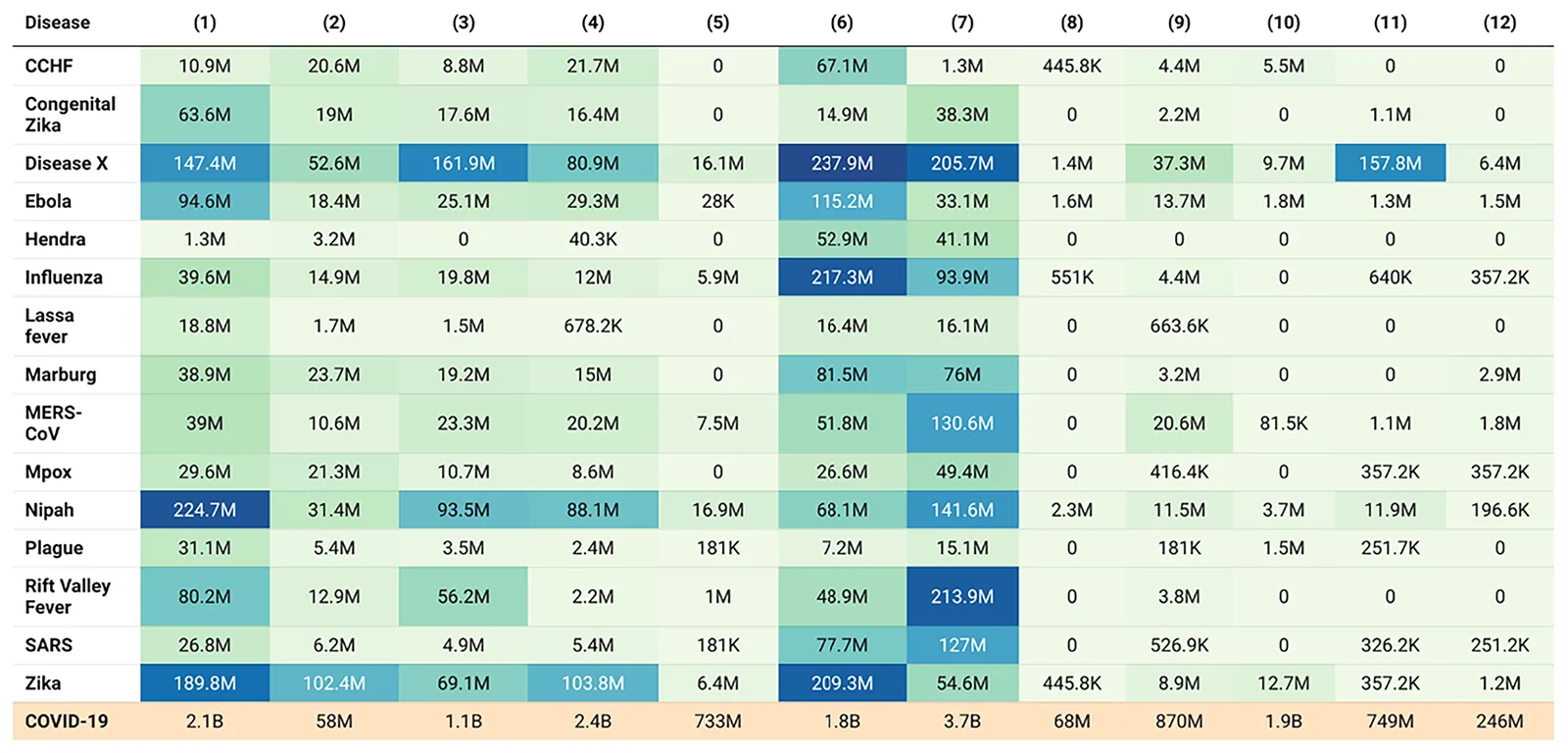
Total known funding distribution across Pandemic PACT list of diseases and research areas. (1) Pathogen: natural history, transmission and diagnostics; (2) Animal and environmental research and research on diseases vectors; (3) Epidemiological studies; (4) Clinical characterisation and management; (5) Infection prevention and control; (6) Therapeutics research, development and implementation; (7) Vaccines research, development and implementation; (8) Research to inform ethical issues; (9) Policies for public health, disease control and community resilience; (10) Secondary impacts of disease, response, and control measures; (11) Health systems research; (12) Research on capacity strengthening. Funding amounts are included only when they have been published by the funder. Financial commitment information is available for 68.7% of the grants in the Pandemic PACT database (n = 13,994/20,362). Although grants may be awarded to grants covering multiple diseases, this is uncommon, with only 6.6% of grants with known financial commitments involving funding for grants covering more than one disease (n = 927/13,994). In such cases, there is a possibility of double counting, as the same financial commitment may be recorded under multiple diseases. In Extended Data 4, we illustrate the global distribution of total grants by disease available in the Pandemic PACT database. Data cover the period from 2020 to 1 March 2025.

Vaccine research stands out as a key investment area, with over $100 million allocated to grants for each of the following diseases since 2020: the concept of ‘Disease X,’ Mpox, Pandemic-prone influenza, Rift Valley Fever, and SARS. However, certain areas remain critically underfunded. Notably, we did not identify in the database grant commitments allocated to infection prevention and control research for diseases such as CCHF, Congenital, Hendra, Marburg, MERS-CoV, or Nipah.

In the following section, we provide a breakdown of how grant commitments have been allocated across research sub-categories and diseases (apart from grants solely focussed on COVID-19) within each of the twelve Pandemic PACT broad research areas. This analysis aims to offer a clearer understanding of where investments have been concentrated and where critical gaps remain, providing an overview of the funding landscape for research on epidemic-prone diseases.

### Global funding by epidemic-prone infectious diseases and sub-priority research areas

By examining the highest levels of known financial commitments allocated to each research sub-category by disease, we can identify both the priorities that have guided funding decisions and potential gaps in the research landscape. Extended Data 3 provides a comprehensive list of the research areas along with their corresponding sub-categories.

As illustrated in Table 2, we identify, for each broad research area, the specific sub-category that received the greatest share of funding. For example, in the area of “Pathogen: natural history, transmission, and diagnostics,” investment for diseases such as CCHF, Pandemic-prone influenza, and plague was primarily directed toward immunity research. In contrast, research on the concept of ‘Disease X’ was largely concentrated on diagnostics.

**Table 2.** Total known funding distribution across Pandemic PACT list of diseases and research sub-categories.

Disease	Pathogen	Animal	Epidemiology	Clinical	IPC	Therapeutics	Vaccines	Ethics	Policy	Secondary impacts	Health systems	Capacity strengthening
**CCHF**	$43.5	$10.5	$13.6	$11.2	$0.0	$13.6	$34.2	$0.0	$2.1	$0.0	$1.0	$0.0
	(Immunity)	(Vector control)	(Disease surveillance)	(Prognostic factors)	-	(Pre-clinical)	(Pre-clinical)	-	(Policy)	-	(Service delivery)	-
**Congenital Zika**	$9.5	$19.9	$8.8	$20.5	$0.0	$67.1	$0.9	$0.4	$4.4	$5.5	$0.0	$0.0
	(Pathogen morphology)	(Vector control)	(Disease surveillance)	(Disease pathogenesis)	-	(Pre-clinical)	(Design and administration/Induced immunity)	(Social determinants)	(Policy)	(Health)	-	-
**Disease X**	$65.0	$40.4	$102.2	$41.0	$7.2	$130.0	$66.4	$0.9	$20.0	$8.2	$138.6	$3.9
	(Diagnostics)	(Animal source and routes)	(Disease surveillance)	(Disease pathogenesis)	(Control measures)	(Pre-clinical)	(Pre-clinical)	(Research)	(Policy)	(Other)	(Leadership and governance)	(Cross-cutting)
**Ebola**	$47.3	$17.7	$14.4	$27.4	$0.03	$114.8	$15.8	$1.4	$7.0	$1.6	$0.6	$0.7
	(Pathogen morphology)	(Animal source and routes)	(Disease transmission)	(Disease pathogenesis)	(Unspecified)	(Pre-clinical)	Design and administration	(Governance)	(Community engagement)	(Health)	(Technologies)	(Cross-cutting)
**Hendra**	$0.9	$3.2	$0.0	$0.04	$0.0	$94.0	$82.2	$0.0	$0.0	$0.0	$0.0	$0.0
	(Pathogen morphology)	(Animal source and routes/vector biology)	-	(Disease pathogenesis)	-	(Pre-clinical)	(Pre-clinical)	-	-	-	-	-
**Influenza**	$108.4	$27.4	$45.1	$62.2	$6.8	$33.1	$53.0	$2.3	$7.5	$1.9	$11.5	$0.2
	(Immunity)	(Animal source and routes)	(Disease transmission)	(Disease pathogenesis)	(Restriction measures)	(Clinical trial [unspecified phase])	(Pre-clinical)	(Public health measures)	(Policy)	(Economic)	(Technologies)	(Institutional level)
**Lassa fever**	$19.3	$14.9	$11.7	$12.0	$0.0	$250.4	$93.9	$0.6	$4.4	$0.0	$0.3	$0.4
	(Pathogen morphology)	(Animal source and routes)	(Disease transmission)	(Disease pathogenesis)	-	(Pre-clinical)	(Pre-clinical)	(Research)	(Policy)	-	(Service delivery)	(Cross-cutting)
**MERS-CoV**	$22.1	$23.7	$18.5	$13.9	$0.0	$81.0	$37.5	$0.0	$2.9	$0.0	$0.0	$2.9
	(Pathogen morphology)	(Animal source and routes)	(Disease transmission)	(Disease pathogenesis)	-	(Pre-clinical)	(Pre-clinical)	-	(Treatment hesitancy)	-	-	(Individual level)
**Marburg**	$9.3	$1.2	$0.6	$0.6	$0.0	$16.1	$9.4	$0.0	$0.6	$0.0	$0.0	$0.0
	(Pathogen morphology)	(Animal source and routes)	(Disease surveillance)	(Disease pathogenesis)	-	(Pre-clinical)	Design and administration	-	(Communication)	-	-	-
**Mpox**	$26.3	$8.8	$16.0	$16.7	$3.4	$42.2	$108.6	$0.0	$14.7	$0.1	$1.1	$1.6
	(Pathogen morphology)	(Animal source and routes)	(Disease surveillance)	(Disease pathogenesis)	(Human-animal interface)	(Clinical trial [unspecified phase])	(Phase 2)	-	(Communication)	(Health/Social/Economic)	(Leadership and governance)	(Individual level)
**Nipah**	$21.5	$21.3	$9.2	$8.6	$0.0	$23.9	$42.3	$0.0	$0.4	$0.0	$0.3	$0.4
	(Pathogen morphology)	(Animal source and routes)	(Disease susceptibility)	(Disease pathogenesis)	-	(Pre-clinical)	(Phase 1/Phase 2)	-	(Policy)	-	(Leadership and governance)	(Cross-cutting)
**Plague**	$16.3	$5.4	$2.5	$2.3	$0.2	$0.5	$4.8	$0.0	$0.2	$0.0	$0.3	$0.0
	(Immunity)	(Vector biology)	(Control measures)	(Disease pathogenesis)	(Restriction measures)	(Pre-clinical)	(Pre-clinical)		(Policy)		(Technologies)	-
**Rift Valley Fever**	$25.7	$10.3	$55.6	$2.2	$0.6	$94.0	$114.1	$0.0	$3.5	$0.0	$0.0	$0.0
	(Pathogen morphology)	(Animal source and routes)	(Disease surveillance)	(Disease pathogenesis)	(Restriction measures)	(Pre-clinical)	(Phase 1/Phase 2)	-	(Policy)	-	-	-
**SARS**	$15.6	$5.8	$4.2	$4.6	$0.2	$74.9	$74.9	$0.0	$0.6	$0.0	$0.4	$0.0
	(Pathogen genomics)	(Animal source and routes)	(Disease transmission)	(Disease pathogenesis)	(Restriction measures)	(Pre-clinical)	(Phase 4/Logistics)	-	(Policy)	-	(Leadership and governance)	-
**Zika**	$114.2	$64.9	$43.7	$83.6	$6.4	$197.7	$36.9	$0.5	$4.9	$8.7	$0.4	$0.9
	(Pathogen morphology)	(Vector control)	(Disease surveillance)	(Disease pathogenesis)	(Control measures)	(Pre-clinical)	(Pre-clinical)	(Social determinants)	(Policy)	(Health)	(Leadership and governance)	(Individual/Institutional level)

Funding amounts are included only when they have been published by the funder. Data cover the period from 2020 to 1 March 2025. Financial commitment information is available for 68.7% of the grants in the Pandemic PACT database (n = 13,994/20,362). Although grants may be awarded to grants covering multiple diseases, this is uncommon, with only 6.6% of grants with known financial commitments involving funding for grants covering more than one disease (n = 927/13,994). In such cases, there is a possibility of double counting, as the same financial commitment may be recorded under multiple diseases.

For research on therapeutics, pre-clinical studies emerged as the sub-category that received the most funding for nearly all diseases, except for Pandemic-prone influenza and Mpox, where the dominant investment was in clinical trials with unspecified phase. A similar pattern can be observed in vaccine research, where pre-clinical studies generally received the largest share of funding, though this was not consistent across all diseases. For instance, research on Mpox focussed more on Phase 2 clinical trials, while funding for Nipah and Rift Valley fever was largely concentrated on both Phase I and Phase II trials.

Overall, across the twelve broad research areas tracked by Pandemic PACT, vaccine research received the highest level of potential financial commitment ($1.2 billion), followed by pathogen-related research ($934.9 million) and therapeutics ($751.6 million). The Pandemic PACT data indicate that financial commitments were primarily concentrated in early-phase and pre-clinical studies, particularly for diseases such as Zika, Lassa fever, Rift Valley fever, Mpox, and the concept of ‘Disease X’. Notably, Disease X attracted substantial investment across nearly all research domains, with especially relative high allocations in health systems research. In contrast, several diseases, including Hendra virus infection, Marburg virus disease, and Congenital Zika, received limited or narrowly focused funding. Other research areas, such as epidemiology ($466.3 million), clinical characterisation and management ($348.9 million), and public health policy and community resilience ($139.1 million), displayed relative variability in funding by disease and sub-category, with several diseases receiving no known commitments in specific areas. Overall, the data reflect a concentration of resources in certain diseases and clinical trial phases, revealing important gaps, particularly in later-stage trials, health systems, ethics, and capacity-building efforts. Extended Data 5 provides a detailed breakdown of known funding distributions across the Pandemic PACT list of diseases by research sub-category.

### Study populations in epidemic-prone infectious diseases research

The Pandemic PACT database provides relevant information on the study populations included in the research funded by the grants. The database categorises study subjects into seven groups: animals, bacteria, humans, disease vectors, viruses, environment, and other specified populations not listed. Some grants include multiple study populations, such as examining both human populations and viruses simultaneously.

Apart from grants exclusively focused on COVID-19, across all diseases in the Pandemic PACT database, less than 25% of grants involve human populations as study subjects, with the majority focussing on other populations, including animals, viruses, disease vectors, or have an unspecified study population. Mpox is the only exception, with 40% of grants for this disease involving human populations as one of the study subjects. Figure 13 provides a detailed view of the total number of grants allocated across different study populations by disease, alongside the proportional breakdown of the total number of grants. Among the diseases, Hendra, for instance, has twelve grants, with only two of them involving human populations as study subjects. Similarly, Marburg has a relatively low proportion of grants (8%) involving human populations. In contrast, diseases such as CCHF, the concept of ‘Disease X’, MERS-CoV, and Nipah each have nearly 30% of grants covering human populations as study subjects. Additionally, the concept of ‘Disease X’ has the highest proportion of grants with unspecified study populations, accounting for 24% of the total grants allocated to this disease.

**Figure 13.**
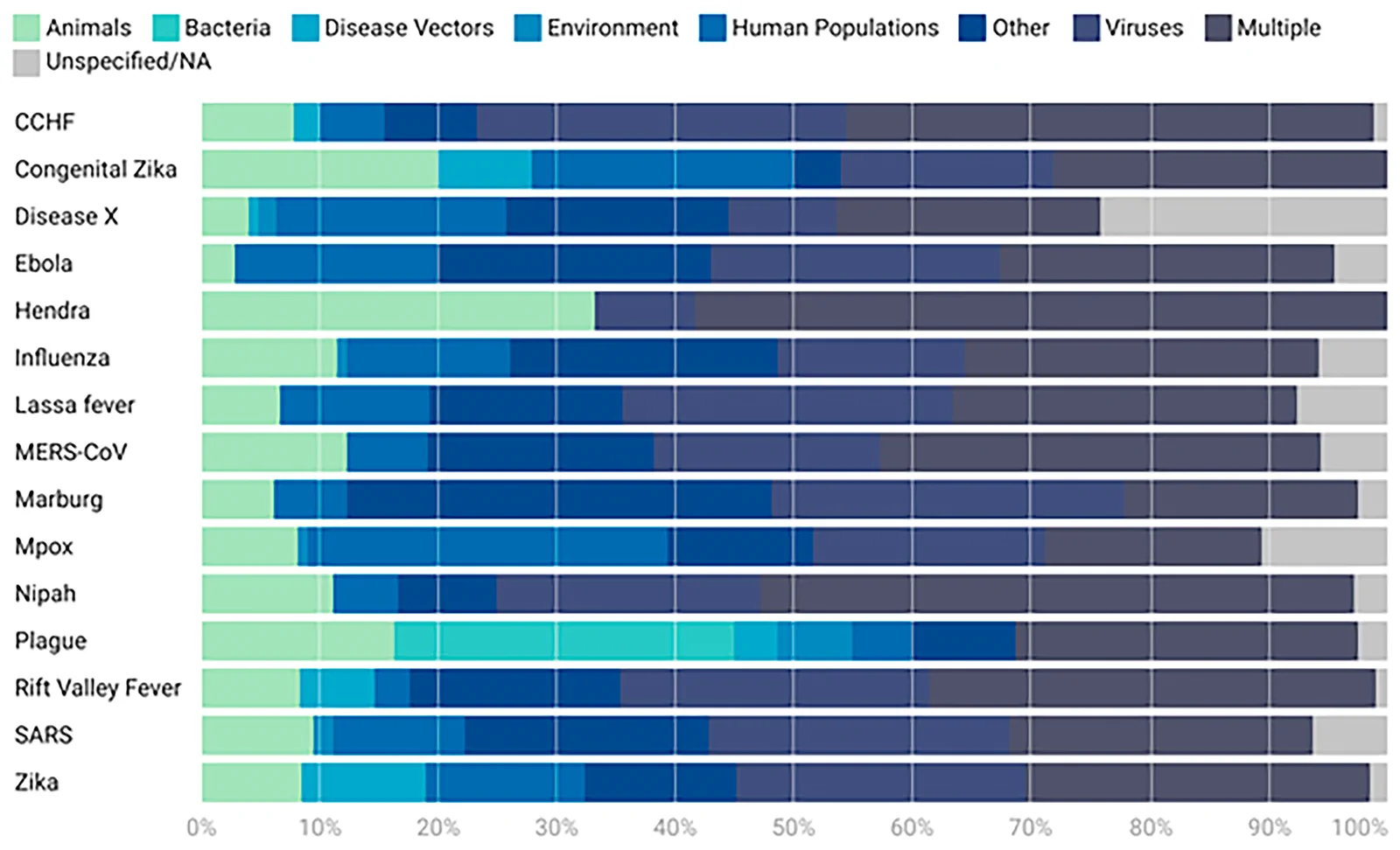
Percentage and total number of grants for research involving human population subjects across Pandemic PACT list of diseases. Distribution of the total number of grants allocated across different study populations by disease. Data cover the period from 2020 to 1 March 2025.

### Specific human populational groups in epidemic-prone infectious diseases research

When human populations are included in the research, the Pandemic PACT database provides insights into several sociodemographic characteristics of these populations. This information is crucial for improving the coordination and efficient allocation of resources, especially during health emergencies, as specific populations may face heightened risks. Pandemic PACT categorises human populations according to ethnicity, age group, geographical scope (urban or rural), vulnerable group, and occupational group. Detailed descriptions of each characteristic and its corresponding categories can be found in the Pandemic PACT published protocol. ^18^

Figure 14 presents the distribution of grants for research involving human populations across the diseases in the Pandemic PACT database, alongside the proportional breakdown of grants targeting specific population groups. For instance, of the twelve grants related to Congenital Zika involving human populations, 75% focused on children, while 25% included women. This is consistent with the nature of Congenital Zika, which primarily involves birth defects and complications arising from Zika virus infection during pregnancy. Similarly, in research on Mpox, 26.5% of the 49 grants that involved human populations targeted vulnerable groups, particularly men who have sex with men, who have been disproportionately affected by recent outbreaks.

**Figure 14.**
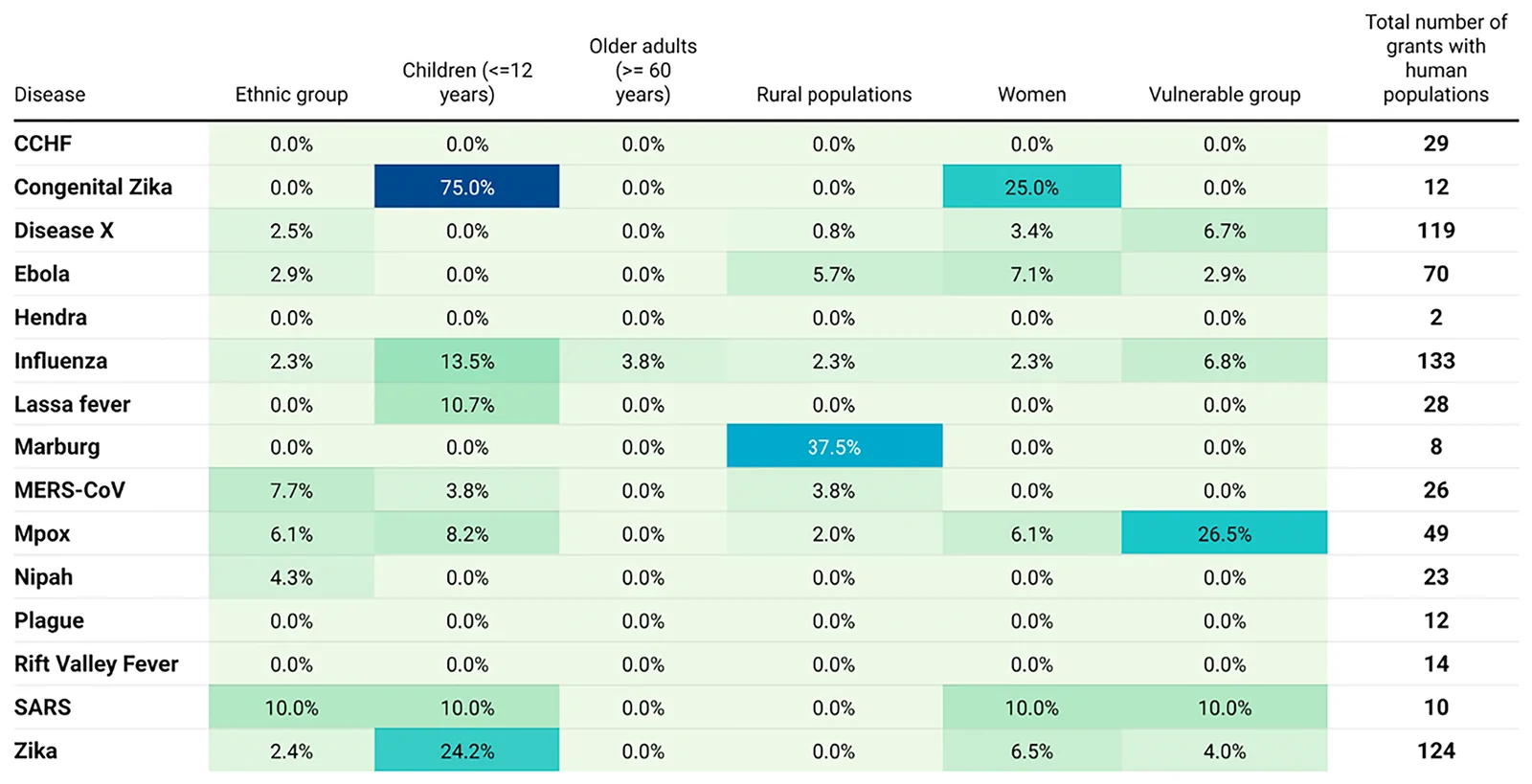
Percentage of grants involving specific populational groups across Pandemic PACT list of diseases. Distribution of the total number of grants allocated across different populational groups by disease. Data cover the period from 2020 to 1 March 2025.

The only disease where older adults were specifically included as study subjects is Pandemic-prone influenza. This focus may be explained by the fact that older adults are at a higher risk of severe complications from influenza and related health issues. Additionally, research on Marburg included 37.5% of grants involving human populations that focused on rural populations, reflecting the tendency for outbreaks of this virus to disproportionately affect such areas. These findings illustrate the existing distribution of research investments across different sociodemographic groups. While this information does not provide insight into whether the investments are sufficient, they offer a mapping of current research priorities, which can inform ongoing and future efforts to address the needs of specific populations during health emergencies.

### Study type in epidemic-prone infectious diseases research

The Pandemic PACT database also provides information on the study type of funded research when specified. Pandemic PACT classifies research as either clinical or non-clinical, with clinical studies broadly defined as those involving primary data collection on humans to diagnose, characterise, describe, or conduct disease surveillance.

Across all diseases in the Pandemic PACT database, the majority of grants support non-clinical research. Some diseases, such as Congenital Zika, Hendra virus infection, and Marburg virus disease, received no funding for purely clinical research. Mpox has the highest proportion of grants dedicated exclusively to clinical research, accounting for 14% (n = 17/122) of its total funded grants. Additionally, four other grants have mixed-study types, meaning they include both clinical and non-clinical research components. Figure 15 provides a detailed overview of the total number of grants supporting clinical research across diseases in the Pandemic PACT database, alongside the proportional distribution of grants for non-clinical and mixed-type research.

**Figure 15.**
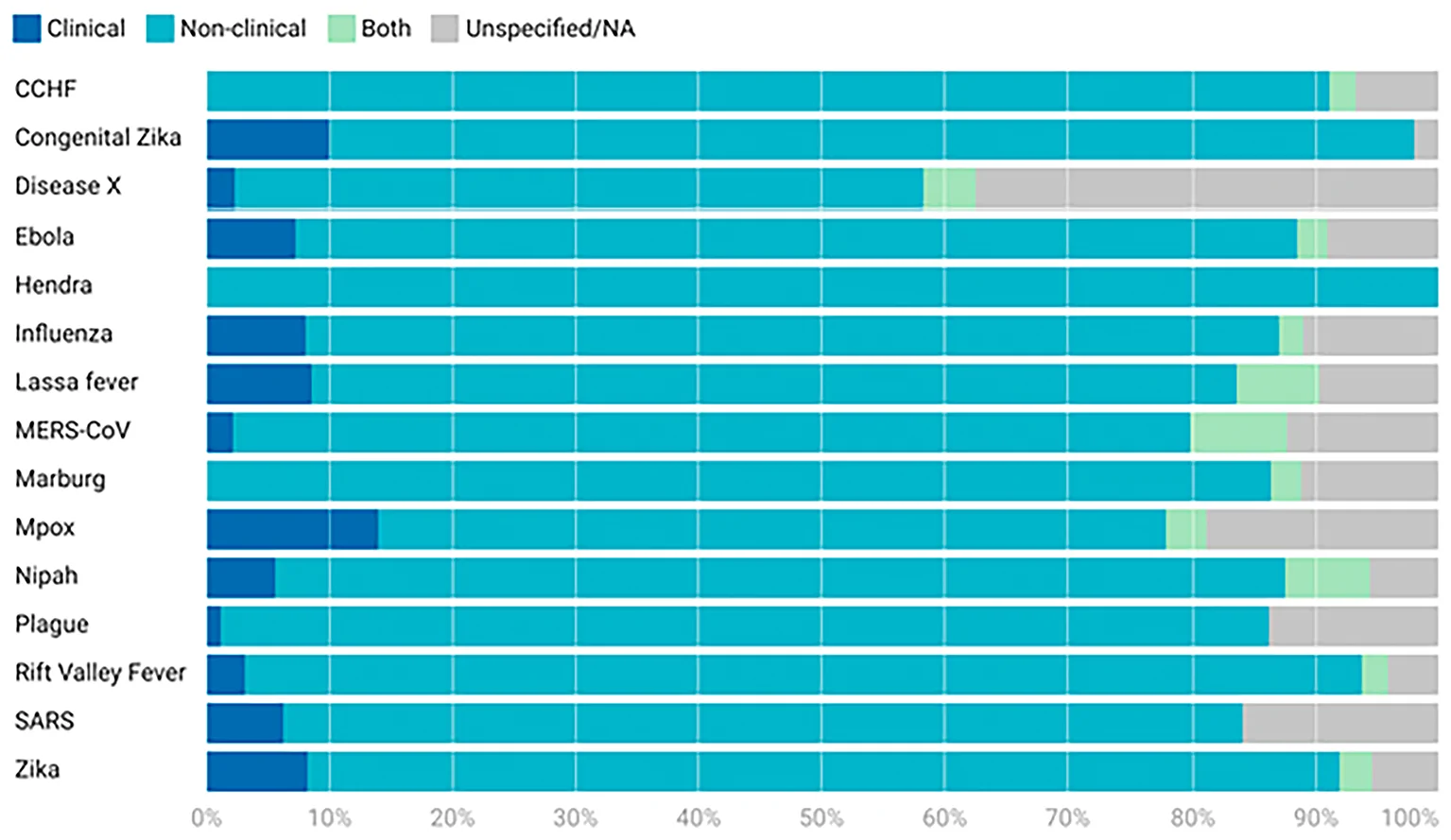
Percentage and total number of grants by study type across Pandemic PACT list of diseases. Distribution of the total number of grants allocated across different study types by disease. Data cover the period from 2020 to 1 March 2025.

While the number of grants allocated to clinical versus non-clinical research provides useful comparative information, it does not fully capture the financial commitments associated with each study type. As shown in Figure 16, although non-clinical research receives a higher proportion of total grants across diseases, financial commitments do not always follow the same pattern. For instance, despite clinical research accounting for only 14% of Mpox grants, it received $14.3 million in known financial commitments—nearly four times the funding allocated to non-clinical Mpox research. In contrast, all funding for Hendra virus infection was directed toward non-clinical research. Notably, a substantial portion of financial commitments for the concept of ‘Disease X’ were allocated to grants that did not specify the study type, amounting to $462.9 million. Figure 16 provides a detailed breakdown of financial commitments across study types and diseases, illustrating investment priorities for clinical and non-clinical research.

**Figure 16.**
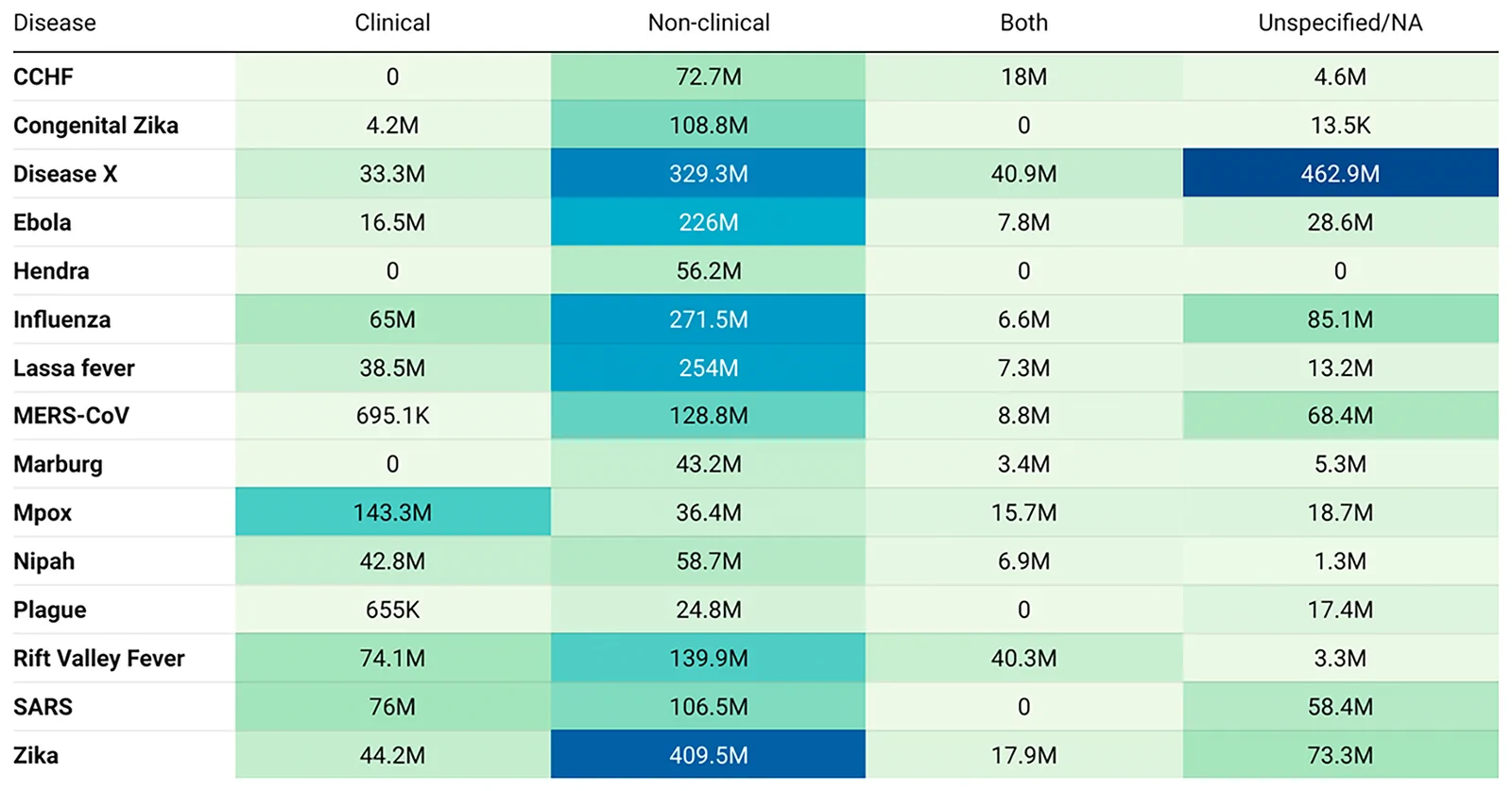
Total known funding distribution across Pandemic PACT list of diseases and study type. Funding amounts are included only when they have been published by the funder. Data cover the period from 2020 to 1 March 2025. Financial commitment information is available for 68.7% of the grants in the Pandemic PACT database (n = 13,994/20,362). Although grants may be awarded to grants covering multiple diseases, this is uncommon, with only 6.6% of grants with known financial commitments involving funding for grants covering more than one disease (n = 927/13,994). In such cases, there is a possibility of double counting, as the same financial commitment may be recorded under multiple diseases.

## Discussion

The COVID-19 pandemic prompted unprecedented levels of research funding. Importantly, one of its lasting impacts has been a growing recognition of the need to strengthen research preparedness for a broader range of high-risk, pandemic-prone infectious diseases. ^21^ Pandemic PACT is designed to facilitate that greater preparedness, through providing a broader understanding of the existing research investments and identifying critical investment gaps across infectious diseases, research areas, and geographic scope as well as the nature of funders who support pandemic-prone infectious disease research, particularly in LMICs. This paper presents the first baseline analysis of the Pandemic PACT dataset. By extending the scope of analysis beyond COVID-19 to the full range of pandemic-prone diseases tracked by Pandemic PACT, this study advances prior work, including the COVID CIRCLE living mapping review, ^3^ which, while comprehensive in its coverage of COVID-19 research funding, did not provide a systematic analysis of the broader infectious disease research funding landscape.

Our baseline analysis shows that, while funding patterns have generally aligned well with the Pandemic PACT list of priority research areas, many remain significantly limited. In numerous diseases, funding is concentrated in a limited set of research areas, with notable gaps in diagnostics, later-phase clinical trials, health systems research, and capacity strengthening. One persistent investment gap in infectious disease and pandemic preparedness research is the continued lack of funding for research to ethical issues. This pattern was already present in the COVID CIRCLE portfolio ^3^ and has carried over into the broader Pandemic PACT dataset across broader diseases. Despite widespread recognition of the importance of ethical considerations, ^12, 25, 26^ this area remains to receive limited attention within global research funding priorities.

The Pandemic PACT dataset also reveals that several areas of research have received comparatively less investment across the broader landscape of pandemic-prone infectious diseases. Though it was pivotal to the COVID-19 pandemic response, financial commitments towards research on infection prevention and control rank among the lowest of the twelve broad research categories in the Pandemic PACT dataset. Similarly, funding for research on policies for public health, disease control, and community resilience, as well as on the secondary impacts of disease outbreaks and response measures, has declined in prominence compared to the COVID-19 research portfolio. Funding for diagnostics, which in the Pandemic PACT research categorisation framework is a sub-category within the broader pathogen-related research area, has also declined relative to COVID-19. This discrepancy may reflect the exceptional nature of the global response to COVID-19, where the urgent need to contain a rapidly spreading virus catalysed an unprecedented mobilisation of political will and research investments. In contrast, other high-risk infectious diseases often lead to more localised or slower-moving outbreaks that do not trigger the same level of sustained attention or funding urgency. As a result, critical areas of research that are important to effective preparedness and long-term resilience have received comparatively lower levels of investment.

In contrast, one shift compared to the COVID-19 research investments is the increasing financial commitments allocated to research on animal and environmental research and research on disease vectors. During the COVID-19 pandemic, this area received relatively lower levels of funding. ^3^ However, in the Pandemic PACT dataset, research investments in this area aligned more frequently across diseases. While the inclusion of vector-borne diseases in the list of pandemic-prone diseases tracked by Pandemic PACT, such as CCHF, Lassa fever, Rift Valley fever, and Zika, helps account for increased funding in areas like vector biology and vector control strategies, it does not fully explain the relative rise in investment in this broader research area. This trend might also reflect a growing alignment with the One Health approach, ^27^ which emphasises the interconnectedness of human, animal, and environmental health in the emergence and transmission of infectious diseases, suggesting a greater recognition of the complex ecological dynamics that drive pandemics.

A relatively greater share of research funding in the Pandemic PACT dataset continued to focus on vaccine and therapeutic development and implementation, as well as on pathogen morphology, transmission dynamics, and diagnostics. However, even within these high-priority areas, notable gaps remain. Pathogen-focused research was largely concentrated on Nipah, Zika, and the concept of ‘Disease X’. While studies on pathogen morphology, viral shedding, and natural history received comparatively higher levels of investment, areas such as diagnostics, disease modelling, and the environmental stability of pathogens attracted less attention. In therapeutic research, the majority of funding was directed toward pre-clinical studies, which encompass early-stage drug development activities such as laboratory-based experiments and testing in animal or cell models. A notable share also supported clinical trials without a specified phase, including randomised controlled trials. Yet, key gaps persist: no funding was identified for Phase 4 therapeutic trials, while research into adverse events associated with therapeutic use and Phase 1 trials was limited to Mpox- and Zika-related studies, respectively. Vaccine research showed a somewhat broader spread, with most funding directed toward Phase 1 and Phase 2 trials, particularly for Lassa fever, MERS-CoV, Mpox, Nipah, and Rift Valley fever. However, similar to therapeutics, no funding was allocated to Phase 4 vaccine trials, except for financial commitments directed to research on SARS. Phase 3 trials received funding exclusively for Mpox and Ebola. While funding was allocated to all diseases on the Pandemic PACT list for this broad research area, some diseases lacked funding for any vaccine clinical studies. This was the case for CCHF, Congenital Zika, and Hendra. Notably, pre-clinical research received commitments across the entire spectrum of epidemic-prone diseases included in Pandemic PACT.

The disaggregation of research grants by population groups in the Pandemic PACT dataset offers useful insights into how sociodemographic considerations are being integrated into research on epidemic-prone diseases and serves as an important tool for informing policy and ensuring that research activities engage the full range of populations affected by epidemic-prone diseases. In some instances, study populations appear to reflect disease-specific risk profiles. For example, research on Congenital Zika included children and women and Mpox-related grants showed some attention to vulnerable groups. However, the overall inclusion of specific population groups remains limited across the dataset. Ethnic groups were included in a small number of grants related to MERS-CoV, Mpox, and SARS, while representation in research on other diseases was either minimal or entirely absent from the dataset. Similarly, vulnerable groups were not identified in grants related to CCHF, Congenital Zika, Hendra, Lassa fever, Marburg, MERS-CoV, Nipah, plague, and Rift Valley fever. These gaps highlight the need for more systematic inclusion of diverse population groups in research design and funding strategies, to ensure that future preparedness efforts are both equitable and responsive to the needs of those most at risk.

Another clear gap identified in our dataset is the relative lack of research conducted in LMICs, which highlights inherent structural imbalances within the global health research ecosystem. Despite growing recognition of the need for more equitable research investment, particularly in regions that bear a disproportionate burden of infectious disease outbreaks, ^5, 28^ funding continues to be concentrated in HICs, although our dataset likely underestimates LMIC research investments, as some funding is intramural and therefore not captured in publicly available sources. While intramural funding occurs in both HICs and LMICs, the limitation is likely more pronounced in LMICs, where a greater proportion of domestic research activity may be funded through such mechanisms and therefore remain less visible to analyses relying on publicly available data.

Since the COVID-19 pandemic, the global distribution of funding has shifted slightly, with more funding calls and announcements related to LMICs and some domestic funding captured, such as in Brazil and India. Although some funders based in HICs, especially in the UK, US, and Canada, have made efforts to direct research investments toward LMICs, along with the growing recognition that context-specific research is needed in LMICs, our data shows that only a small proportional expansion of funding dedicated to context-specific research priorities. This persistent concentration reflects structural barriers within international funding mechanisms that systematically disadvantage LMIC-based applicants, limited institutional capacity in many LMIC settings, and geopolitical factors that may shape where research funding flows. Moreover, expected declines in official development assistance (ODA) in 2025, ^29^ combined with planned cuts in US spending on medical and scientific research, public health, and disease-prevention programmes, are likely to further affect LMICs and exacerbate existing vulnerabilities There remain clear research gaps relating to health systems, clinical characterisation management, disease severity studies and effective public health interventions in various settings. These imbalances limit the production of context-specific evidence that is critical for effective and locally relevant responses.

To ensure that research investments translate into meaningful impact, there is an urgent need for stronger coordination and continuous alignment with local research priorities. This is particularly critical given the diverse nature of disease outbreaks, which vary in their modes of transmission, affected populations, and contextual needs. Real-time tracking of research funding is crucial in such fast-moving environments, where timely, informed decisions can shape the trajectory of an effective response. Beyond pandemic contexts, this capacity for timely and transparent monitoring holds significant value for other global research efforts, where coordinated action across borders and disciplines is increasingly essential. It should be noted that this analysis captures financial commitments rather than research outputs or outcomes. Funding allocation does not in itself guarantee successful implementation, knowledge generation, or policy uptake. Linking investment data to measurable downstream outcomes, such as published findings, product development milestones, or strengthened health systems, remains an important direction for future work. Nonetheless, the persistence of fragmented, underpowered, and often duplicative nationally funded studies highlights the need for both funders and researchers to adopt more strategic, collaborative approaches to maximise the efficiency and utility of research during health emergencies. Tools such as the Pandemic PACT tracker play a crucial role in enabling this shift, facilitating transparency, fostering global collaboration, and strengthening the collective capacity to direct research funding where it is most needed and where it can have the greatest impact.

## Conclusion

We have provided a comprehensive review and descriptive analysis of funded research across infectious diseases with pandemic potential, as captured in the Pandemic PACT dataset, representing an important step forward in our ability to track, map, and evaluate global research funding for pandemic disease threats. Key research gaps in funding remain in diagnostics, health systems, and ethics research, while most vaccine and therapeutic studies are still at early stages. Vulnerable populations are rarely included, and context-specific research in LMICs receives limited financial support. From a health policy perspective, these findings point to the need for greater equity considerations in how research investments are designed and allocated, particularly in light of anticipated declines in official development assistance and the increasing need for transparent, real-time tracking of funding flows. We encourage the research community to make use of these findings and the Pandemic PACT tracker tool to inform evidence-based decisions on future research prioritisation, guided by an understanding of existing research efforts. We also urge research funders to continue contributing their data to the tracker, thereby enhancing its utility as a resource for improving pandemic preparedness through greater transparency and early visibility of research funding and activity during emerging outbreaks.

## Data Availability

The continuously updated Pandemic PACT data and the specific dataset used for this study are openly available on Figshare platform at https://figshare.com/s/9e94a0e039df18316faa and https://doi.org/10.25446/oxford.30296383, respectively. The data can also be explored and exported on the online public dashboard at https://www.pandemicpact.org, which includes high-quality figures and charts, also available for downloading. Figshare: Extended data for: ‘Living mapping review of global research funding for infectious diseases with a pandemic potential – Pandemic PACT Grant Tracker. https://doi.org/10.25446/oxford.30296383 This project contains the following extended data:
• Figures 1–16.png • Extended Data 1. Total number of grants, known financial commitments, and percentage of grants with funding amount information available by funding organisation, sorted by total known financial commitments.docx • Extended Data 2. Funding data sources.docx • Extended Data 3. Pandemic PACT research categories and subcategories.docx • Extended Data 4. Pandemic PACT research categories and subcategories.docx • Extended Data 5. Total known funding distributions across the Pandemic PACT list of diseases by research sub-category.docx Figshare: PRISMA checklist for ‘Living mapping review of global research funding for infectious diseases with a pandemic potential – Pandemic PACT Grant Tracker. https://doi.org/10.25446/oxford.30296383 Data are available under the CC BY 4.0 license.

## Footnotes

^1^
Country income classifications were determined based on the Official Development Assistance (ODA) recipient list, as defined in the OECD’s 2024 and 2025 flows report. A detailed list of countries classified as low- and middle-income can be found at https://www.oecd.org/en/topics/sub-issues/oda-eligibility-and-conditions/dac-list-of-oda-recipients.html
