# Living mapping review of global research funding for high-risk pathogens-Pandemic PACT Grant Tracker

**DOI:** 10.12688/wellcomeopenres.24958.3

**Published:** 2026-11-02

**Authors:** Rodrigo Furst, Emilia Antonio, Shanthi Levanita, Zaharat Kadri-Alabi, Johannes Paolo Cerrado, Nusrat Jabin, Susan Khader Ibrahim, Thomas Mendy, Adrian Bucher, Alice Norton

**Affiliations:** 1 Policy and Practice Research Group, Pandemic Sciences Institute, University of Oxford, Oxford, Select, OX3 7BN, UK; 2 GloPID-R Research and Policy Team, Policy and Practice Research Group, Pandemic Sciences Institute, University of Oxford, Oxford, Select, OX3 7BN, UK; 3 UK Collaborative on Development Research, London, UK

**Keywords:** Living systematic review, infectious diseases, research funding, coordination, pandemic preparedness, global health policy

## Abstract

**Background:**

The COVID-19 pandemic spurred an unprecedented global research response, highlighting the need for better coordination amid limited resources and the ongoing threat of emerging diseases. The Pandemic PACT Programme has developed a live database tracking funded research on high-risk pathogens and broader preparedness efforts to facilitate improved pandemic preparedness. This paper presents the first analysis update of this dataset, examining trends in research funding identified by the Pandemic PACT programme by disease, geography and research topic, and identifying which funders are supporting high-risk pathogen research, particularly in low- and middle-income countries (LMICs).

**Methods:**

As of December 6, 2025, the database includes 28,176 awards from 330 funding organisations spread across 60 different countries, representing at least $25.9 billion in financial commitments. We conducted a living mapping review to provide an open, accessible analysis of the funded research portfolio on high-risk infectious diseases. The review analyses funding trends from 2020 to 2025 across high-risk pathogens and research areas, identifying key investment priorities and potential gaps.

**Results:**

Analysis of the Pandemic PACT dataset reveals an uneven global research investment across high-risk infectious diseases. While funding has generally aligned with research priorities, research around diagnostics, health systems research, and ethics are the focus of comparatively fewer research awards. Although vaccine and therapeutic research dominate the portfolio, much of it remains focused on pre-clinical and early-phase trials. There is, however, increasing attention to vector and environmental research in comparison to prior analyses on COVID-19 research. Investments remain concentrated in high-income countries, with limited financial support for context-specific research led by institutions in LMICs. The stated inclusion of populations identified to be vulnerable in funded awards is also limited.

**Conclusions:**

Our findings reveal critical gaps and disparities in global pandemic preparedness research funding through research awards identified and collected by Pandemic PACT. Investments remain concentrated in high-income countries, with limited funding for LMIC-led research and identifyable representation of vulnerable populations across the funded research portfolio. The relatively lower levels of investment in diagnostics, health systems research, and ethics indicate potential gaps within the current high-risk pathogen research portfolio. The living design of this review will enable ongoing monitoring of these gaps, providing real-time evidence to support funder coordination and policy decisions.

## Plain Language Summary

The COVID-19 pandemic showed how quickly infectious diseases can spread and highlighted the need for better global research coordination. The Pandemic PACT Programme has created a live database that tracks research funding on diseases that could cause future pandemics, helping to improve preparedness.

As of March 2025, the database includes over 20,000 grants from more than 300 funders across 58 countries, representing at least $14.4 billion in financial support. We reviewed this data to understand global research funding trends from 2020 to 2024, focusing on priority diseases and research areas.

Our analysis shows that funding is unevenly distributed. Vaccine and therapeutic research receive most of the financial support, but much of it focuses on early-stage clinical trials. Areas such as diagnostics, health systems research, and ethics receive comparatively less attention. Funding is largely concentrated in high-income countries, with limited support for research led by institutions in low- and middle-income countries. Vulnerable populations are also rarely included in funded studies.

These findings highlight gaps in global research investment and can help guide better coordination and decision-making to strengthen pandemic preparedness worldwide.

## Introduction

The COVID-19 pandemic triggered an unprecedented flow of funding for research, resulting in a proliferation of publications and evidence generation. However, it also posed significant challenges for coordination of funding, research implementation, and evidence synthesis. Ultimately, despite the wealth of relevant and impactful knowledge generated by this research response, duplication and redundancy were unavoidable. ^1^

In 2020, the World Health Organisation (WHO), in collaboration with Global Research Collaboration for Infectious Disease Preparedness (GloPID-R), developed the Coordinated Global Research Roadmap: 2019 Novel Coronavirus. ^2^ This roadmap outlined key knowledge gaps and research priorities identified by over 400 global experts ^3^ and was influential in the allocation of resources in the pandemic response. ^4^ The research community responded extensively at unprecedented scale, initiating numerous clinical trials for therapeutics and vaccines. However, as a result of poor design and coordination, many were conducted inefficiently limiting their actionable impact. ^5^ ^,^ ^6^ Moreover, these research programmes and trials were predominantly launched in high-income countries (HICs), leaving low-resource settings with limited research activity despite their growing vulnerability and the need for context specific knowledge. This also highlighted the uneven distribution of global funding for research activity. ^3^ ^,^ ^5^

In response to the critical need to coordinate and synergise funders’ research activities, GloPID-R and UK Collaborative on Development Research (UKCDR) launched the COVID-19 Research Coordination and Learning Initiative (COVID CIRCLE). This joint effort, which was founded on an agreed set of principles, tracked and mapped global research funding ^7^ to the COVID-19 research priorities outlined in the WHO Roadmap, UN Research Roadmap for the COVID-19 Recovery (UNRR), and other priority setting activities. ^8^ ^–^ ^10^ The COVID-19 Research Project Tracker, a live database of funded research with its associated living mapping review tracked over 21,000 COVID-19 grants with at least $8.5 billion invested by over 360 global funders. ^3^ Through a living mapping review of the data captured and various funder and researcher coordination activities, COVID CIRCLE provided an overview of the COVID-19 research landscape. The project provided enhanced identification of gaps and opportunities, and informed ongoing investments and coordination during the pandemic, which aimed to support decision makers in ensuring resources were directed towards the most pressing remaining global research needs. Its impact was highlighted in the *R&D Preparedness Ecosystem: Preparedness for Health Emergencies Report* to the Global Preparedness Monitoring Board ^11^ and in the *WHO Overall Achievements Report on COVID-19* released in 2021. ^12^

The COVID-19 pandemic highlighted pre-existing deficiencies within the global landscape of health research and development, which extend beyond the pandemic period. These limitations are indicative of broader challenges within the R&D ecosystem and have far-reaching consequences for the impact of health research, encompassing research conduct and dissemination of research findings, R&D governance, and research funding models. ^13^ Critically, COVID-19 also exposed the fragility of pandemic financing mechanisms and the structural limitations of existing preparedness funding architectures, which remain poorly suited to sustaining investment across the full range of high-risk diseases between emergencies. ^14^ Effective health systems require not only increased capacity during crises but sustained, well-coordinated research investment across the full spectrum of high-risk diseases in non-emergency periods. ^15^ Furthermore, the persistent challenge of monitoring the health R&D investments presents an obstacle to ensuring the effective use of limited resources in a timely manner. ^16^ This is particularly relevant in the fast-moving context of infectious disease outbreaks, where real-time visibility into research funding at the point of initiation is crucial Evidence-informed funding allocation and coordination can be enabled by providing insights into the nature of the research, its alignment to prioritised areas, its geographical distribution, and the stakeholders involved. ^16^ ^,^ ^17^ Real-time tracking can also ensure agility in responding to emerging needs and localise effort where appropriate. With the aim of building on and improving the COVID CIRCLE initiative, we established the Pandemic Preparedness: Analytical Capacity and Funding Tracking Programme (Pandemic PACT). ^17^

Pandemic PACT has developed a tool to systematically track and analyse prospective and rolling research funding as well as broader research preparedness effort for high-risk infectious diseases, as outlined in our protocol. ^18^ The tool incorporates data designed to be findable, accessible, interoperable and reusable (FAIR). ^19^ All related metadata and database are made available in a machine-actionable and user-friendly interface. This original database of global funding for high-risk disease research is updated regularly and linked to publications through Europe PMC, is aligned to a standardised research ontology and mapped to the relevant policy roadmaps. The programme seeks to facilitate evidence-informed funding and research activation by providing timely information that can enhance the efficient allocation of limited research resources and enable a coherent global research response, thereby strengthening the global health research system. ^18^

Despite the lessons of COVID-19, a comprehensive, real-time analysis of how research funding is distributed across the full range of high-risk pathogens remains absent from the literature, leaving a gap in our understanding of the broader global research investment landscape and its alignment with preparedness priorities. This paper presents the second analysis for the research award funding tracking component of Pandemic PACT, which constitutes the first update to our baseline analysis for our living mapping review (LMR). The baseline analysis served as the starting point of our LMR, which will be regularly updated incorporating newly funded research awards as they become available. The key research questions we aim to answer are (i) “where are the gaps in the global distribution of research funding for high-risk pathogens disaggregated by disease, geography, and research topic?”, and (ii) “which funders are supporting high-risk pathogen research globally, and, specifically, in low and middle-income countries (LMICs)?”

## Methods

### Protocol updates

Our methodology is documented in our previously published protocol. ^18^ This includes the eligibility criteria, data sources and search strategy, data flow and processes, database structure, data governance, and data updates and archiving. Methodological details provided in this paper include updates and specificities of this analysis. No reporting frameworks or risk-of-bias assessments are available for research grants or awards that would be applicable for this analysis.

A key update to the protocol is the expanded coverage of funding sources, which now includes a broader range of non-commercial funders beyond those affiliated with GloPID-R and UKCDR. The complete list of funders captured in the Pandemic PACT dataset is provided in Extended Data 1, while Extended Data 2 details the data sources and the date of the last search. Specifically, the first table lists the funding databases from which data were directly and systematically extracted, whereas the second documents additional sources identified indirectly through those primary databases.

Further, we modified our approach to award categorisation to accommodate awards that lack sufficient information to be categorised or fall into research areas outside our framework. Specifically, we distinguished between two types of cases: awards for which information was insufficient, and awards that clearly did not fit any of the predefined categories. The former was coded as “unspecified,” reflecting the absence of sufficient information, whereas the latter were assigned to “N/A,” indicating a mismatch with our existing classification structure rather than missing data, often corresponding to emerging or cross-cutting research directions.

### Rationale for using a living method

The COVID-19 pandemic prompted a swift response from research funding organisations, supporting both new research projects through rolling and one-off funding opportunities and also repurposing existing awards. This reflects the dynamic and evolving nature of infectious disease funding landscape, which is anticipated to witness continued expansion of funding for ‘priority diseases’ and reactive funding whenever there are outbreaks. Furthermore, funding calls often have short timeframes in outbreak scenarios and allocations may be influenced by rapidly changing needs and contexts.

To capture this dynamic landscape consistently and in near real time, newly funded research grants are sourced monthly from funder databases and added to the Pandemic PACT database in batches as they become available from funders. The living mapping review is subsequently updated at six-month intervals under normal circumstances, providing regular snapshots of the evolving global research funding landscape as reflected by the Pandemic PACT database. This will assist the coordination of ongoing responses from researchers and funders by ensuring that up-to-date information on investment priorities and gaps remains openly accessible. The review is also designed to respond to the accelerated demands of acute health emergencies. In the event of a major outbreak, analyses for the specific disease will be prioritised within the established review cycle, allowing timely insights to be generated for the most pressing public health needs. During a pandemic, the full living mapping review will transition to three-monthly updates, ensuring that the frequency of evidence synthesis keeps pace with the speed of the research and funding response.

### Eligibility criteria

***Start date.*** Newly funded and repurposed awards with a start date on or after January 1, 2020 and retrieved from the funders before December 6, 2025, were included in this baseline analysis, to align with the start of COVID-19 research funding initiatives and the preceding COVID CIRCLE dataset. Awards without any available information on the award or start date were not included in this baseline analysis. We may review this inclusion criteria in future analyses to determine how to leverage other available data for awards that are lacking sufficient information.

***Funders.*** The funders were identified based on their inclusion in the previous COVID-19 funding tracker or their collaborations with other funders, such as participation in joint funding initiatives and membership in funder networks.

***Diseases/pathogens.*** Our original baseline analysis included all diseases listed on the WHO R&D Blueprint 2020 ^2^ priority disease: COVID-19; Crimean-Congo haemorrhagic fever; Ebola and Marburg virus disease; Lassa fever; Middle East respiratory syndrome coronavirus (MERS-CoV) and Severe Acute Respiratory Syndrome (SARS); Nipah and henipaviral disease; Rift Valley fever; Zika virus disease and Disease “X”. Disease “X”, is a concept rather than a specific disease, which represents “the knowledge that a serious international outbreak could be caused by a pathogen currently unknown to cause human disease”. ^20^ Three additional diseases (Pandemic-prone influenza, Mpox and plague) were included following the advice of our expert advisory group which include representatives from research funding organisations and subject matter experts or research groups such as CIDRAP for influenza.

In 2024, the WHO published the Pathogen Prioritization: *A Scientific Framework for Epidemic and Pandemic Research Preparedness* report ^21^ which outlines a new approach of prioritisation of high-risk pathogens focusing on entire pathogen families. Under this framework, WHO identified a set of bacterial and viral families with the potential to cause a Public Health Emergency of International Concern (PHEIC). Pathogen families are stratified by the level of risk posed into low, low to medium, medium and high PHEIC risk with a prototype pathogen identified for some families. The Pandemic PACT programme has now expanded its scope to align with this new framework. Updates to our LMR therefore, include this expanded pathogen coverage of all pathogens with a high-PHEIC risk and associated prototype pathogens under Arenaviridae, Coronaviridae, Enterobacteriaceae, Filoviridae, Flaviviridae, Hantaviridae, Nairoviridae, Orthomyxoviridae Paramyxoviridae, Poxviridae, Phenuiviridae, Togaviridae, Vibrionaceae and Yersiniaceae families, and Pathogen X (family unknown). Similar to the concept of Disease “X”, this “Unknown” category captures the risk posed by currently unidentified pathogens with epidemic or pandemic potential.

### Data processes

Data was sourced directly from research funders using a standardised data collection template or scraped from their databases and was stored in its original format. Basic cleaning to remove special characters was done to improve the clarity of award information. Researchers with expertise in public health, medicine, and in conducting meta-analyses and evidence synthesis reviewed and classified the data according to the Pandemic PACT schema. The list and descriptions of research categories to which grants were classified is presented in the Extended Data 3. This is an updated list from our published research categorisation ontology outlined in Norton et al. ^17^ and the variables were introduced at a higher level in the Pandemic PACT protocol. ^18^ Data underwent peer review by at least two independent reviewers, ensuring independent scrutiny. Disagreements were resolved through joint discussions and a third reviewer where necessary. The standardised training of the reviewers and collaborative meetings ensured consistency of processes. Inter-rater reliability is monitored and mitigated by the use of a coding guide. Training includes familiarisation with coding guidance and definitions and involves a series of practice reviews with support from trained reviewers. In this baseline analysis, data was exported from the Pandemic PACT dashboard. We undertook descriptive analyses of the data to summarise our findings.

### Limitations

A key limitation of the database is that the data from each funder has varying available information, which can result in less refined categorisation when the qualitative details provided were insufficient (for example some funders only list award titles and do not publish abstracts). Therefore, the values assigned by reviewers may not have fully captured all aspects of the awards in relation to research areas of focus, funding amounts, study populations, and study type and other relevant fields in the database. To address this, the data was validated by reviewers with an array of expertise including global health research, policy, and funding, and other areas such as medicine and public health, ensuring that the assigned values were as accurate as possible given the available information. While the database is limited by incomplete funding information (financial data were available for 76% of the awards analysed) it represents the most comprehensive resource currently available, having drawn on all publicly accessible sources. Another key limitation is that we are unable to trace financial flows beyond the lead institutions, as this information is not presently collected systematically by funders, which restricts our ability to fully capture the downstream allocation of funds should the lead institution disperse funds to partner institutions. Additionally, many awards span multiple countries, research areas, pathogen families, and diseases without disclosing how funds are distributed across these categories. This lack of granularity poses challenges for financial analysis. To address this, we provide two estimates for awards spanning multiple diseases, research areas, locations, or study types: a minimum estimate, which includes only awards exclusively focused on a single category (e.g., a single disease), and an upper-bound allocation estimate, which assumes that the full award amount is allocated to each relevant category. As a multi-category award contributes its full amount to the upper-bound estimate for each category it spans, these estimates cannot be summed across categories without substantially overstating the total funding through double counting. The minimum and upper-bound estimates should therefore be interpreted as a plausible range for each individual category, rather than as components of a decomposable total. This approach provides a representation of potential funding distributions while acknowledging the limitations of the available data on how funding is allocated within multi-category awards.

Furthermore, engaging with (and obtaining data from) a broader range of funders than those included proved challenging, either due to lack of publicly available information, contacts, or limited capacity from the funders to contribute to the awards (especially funders with non-English award information). This challenge is compounded by the dynamic nature of the database which expand continually to keep pace with the evolving landscape of research funding for high-risk infectious diseases.

Whilst most research funders operate as independent entities, mechanisms for funding including funding sources and flows vary and can interrelate across various funding entities and funding schemes. In this analysis, we do not account for funding relationships or funding flows among funders unless where entities are explicitly listed as co-funders of awards or where upstream funding sources are indicated. Therefore, contributions of some funders to other entities or funding schemes (which are not explicit) are not reckoned against their total funding investment resulting in an underestimation of their contributions in these instances.

The term award is used instead of project, because a single research project may receive multiple funding sources, each with its own unique identifier or award ID. This means our data analysis might not be able to accurately reflect the number of distinct projects that focus on a particular research priority area. Furthermore, while the baseline analysis used the term *grant*, this first update uses *award* instead. This change reflects the fact that some of the funding captured takes the form of contracts rather than grants. Since our data track research funding regardless of the funding mechanism—and we do not always have detailed information on whether funding is provided through grants or contracts—we use the more inclusive term *award* going forward.

The search terms used to retrieve the awards for the database are limited to English; however, the search terms used are aligned to global ontologies that are intended to be universal.

### Risk of bias

This work employs descriptive and thematic analyses to summarise the scope of these funded awards on research on high-risk diseases. All identified awards were equally reviewed and categorised using the best available information on proposed research topics and objectives. No efforts were made to assess the quality of individual studies or their ability to achieve stated objectives. Potential sources of bias from award selection, quality of reviewed data, and data extraction and classification are addressed through robust monthly searches, template completion by funders and independent assessment and review during project classification respectively, as mentioned in the Information Sources and Search Strategy.

While the tracker and the subsequent analysis aim to provide a comprehensive overview of the research landscape for high-risk infectious diseases, the data collected is more likely to be derived from funders of research that are members of UKCDR (all UK-based) and/or GloPID-R (global membership spanning high income countries (HICs) to low-income countries (LICs) with the majority of national funders based in HICs, ^3^ and a biomedical focus). This may likely result in the findings skewing towards a greater representation of research funded from these organisations and reflecting trends in their respective portfolios (in terms of location, research focus and research activity type) rather than necessarily mirroring the broader landscape.

### Scope of analysis

Due to the disproportionate allocation of funding toward COVID-19, our main analyses in this paper focus on awards that include at least one of the other diseases listed in the Pandemic PACT framework, using awards entirely dedicated to COVID-19 solely as a comparator reference point. This decision was motivated by the consideration that Pandemic PACT data builds on the COVID CIRCLE database (representing 53.5% of the data in Pandemic PACT) and associated Living Mapping Review ^3^ meaning that several comprehensive analyses of COVID-19 funding have already been conducted.

## Results

### Award selection

A total of 306,490 awards were initially retrieved from various web-based data sources using predefined search terms, and screened against the eligibility criteria described in the *Methods* section. An additional 17,632 awards from the COVID CIRCLE dataset were also incorporated. Following data aggregation, deduplication and automated pre-screening were conducted using internally developed Python-based data processing pipelines. These pipelines applied rule-based and metadata-driven approaches to identify and remove duplicate records using combinations of key fields, including award identifiers, award titles, abstracts, funder information, and other associated metadata. Where unique identifiers were unavailable or inconsistent across sources, text-based similarity matching (e.g., on titles and abstracts) was used to detect potential duplicates.

Automated pre-screening procedures were also applied to exclude clearly out-of-scope records based on predefined eligibility criteria, including relevance to pathogens within scope and the nature of the funded activity. In addition, automated processes supported data standardisation and harmonisation, such as normalising funder names, research institutions, and date formats prior to further review. Following the application of these automated procedures, 261,544 records were excluded. The remaining 62,578 awards were imported into our primary database (REDCap) for manual review and validation. During this process, 34,402 awards were excluded, primarily for not meeting the eligibility criteria or for not focusing on pathogens included in our scope. Awards were removed if there was no direct mention of pathogens of interest or information pertaining to pandemic preparedness and response such as for ‘Disease X’ included in the title or abstract. The final dataset used for analysis comprised 28,176 awards. A summary of this process is presented in the PRISMA flow diagram ( Figure 1).

**Figure 1.**
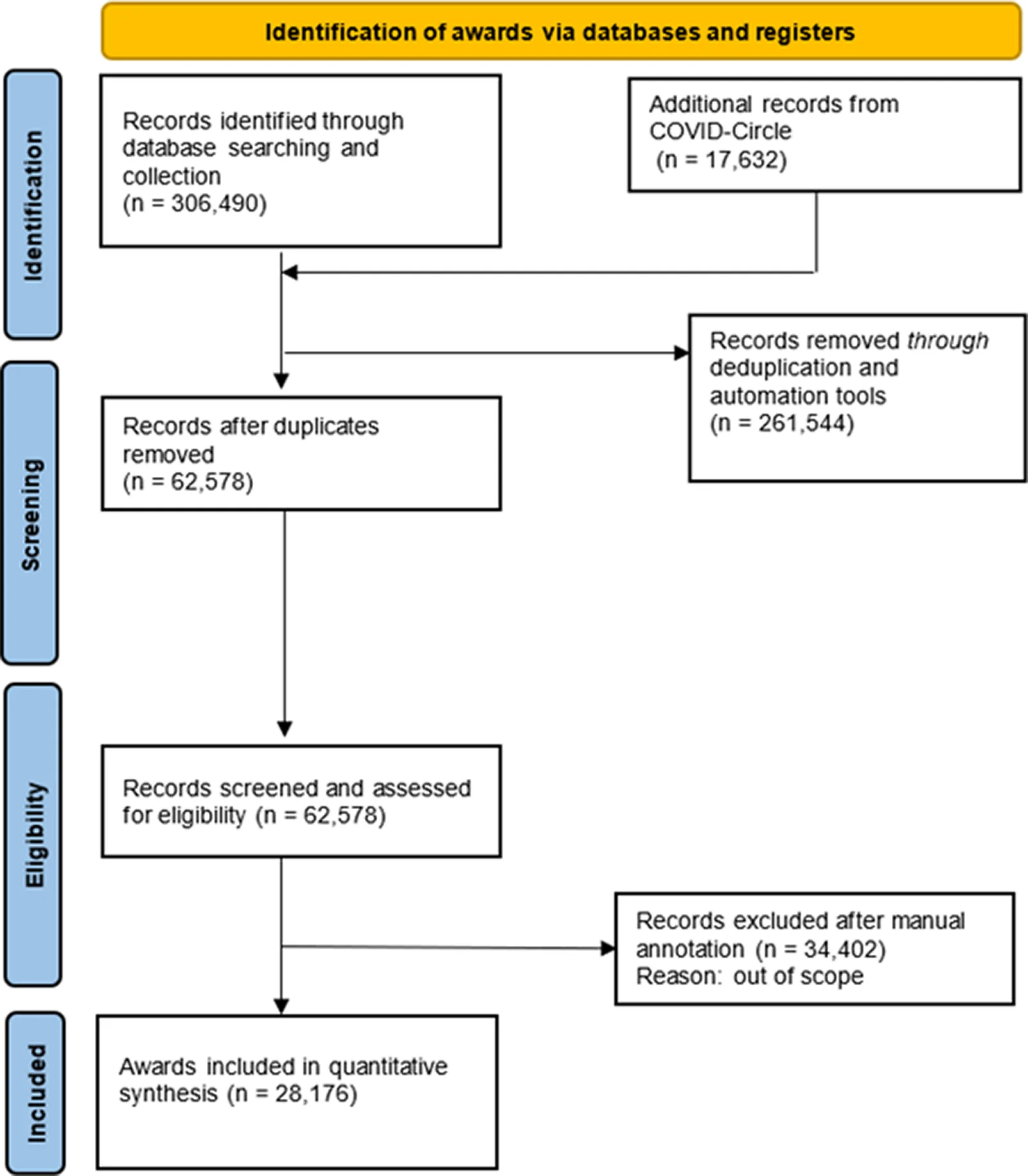
PRISMA flow diagram. Summary of the Pandemic PACT award selection process.

### Data completeness

As of 6 December 2025, a total of 28,176 grants were captured in the Pandemic PACT Tracker. Funding amount information was available for 76% (n = 21,416/28,176) of these awards. Additionally, the award abstract was available for 78.3% (n = 22,056/28,176), and the award title for 99.9% (n = 28,135/28,176) of the awards. Information on the funder-assigned award identifier, research institution, and study subject were available for 73.3% (n = 20,667/28,176), 91.0% (n = 25,633/28,176), and 91.1% (n = 25,669/28,176) of the awards, respectively. Table 1 summarises the availability and sources of data for key Pandemic PACT fields.

**Table 1.** List of key Pandemic PACT variables with corresponding data completeness and data source.

Variable name	Completeness (n)	Completeness (%)	Data source
Funder-assigned award ID	20,667	73.3%	Funder website/provided data
Award title	28,135	99.9%	Funder website/provided data
Award abstract	22,056	78.3%	Funder website/provided data
Start year	28,176	100.0%	Funder website/provided data
End year	17,673	62.7%	Funder website/provided data
Financial commitment	21,416	76.0%	Funder website/provided data
Funder organisation	28,176	100.0%	Funder website/provided data
Research institution name	25,633	91.0%	Funder website/provided data
Research location country	27,514	97.7%	Funder website/provided data
Study subject	25,669	91.1%	Human annotation
Study type	21,267	75.5%	Human annotation

Summary of the availability and sources of data for key Pandemic PACT fields. Data cover the period from 1 January 2020 to 6 December 2025.

### Total known financial commitments

Of the known financial commitments, the award amounts awarded ranged from $111 to $448.3 million, with a median value of $283,569 and a combined total investment of at least $25.9 billion invested by 330 funding entities spread across 60 different countries.

### Funding organisations

In the latest version of the database, awards were most commonly (45.7%, n = 12,888/28,176) made by US-based institutions, with a total known financial commitment of nearly $14.3 billion, representing 55.2% of the total known funding commitments. Of these, the National Institutes of Health (NIH) funded $12.4 billion (86.7% of known US funding commitments) across 9,450 awards, while the National Science Foundation (NSF) and the Gates Foundation funded 1,796 and 777 awards, financing $599.3 million (4.2% of known US funding commitments) and $1.2 billion (8.4% of known US funding commitments), respectively. Figure 2 illustrates the global distribution of total known financial commitments by funder location.

**Figure 2.**
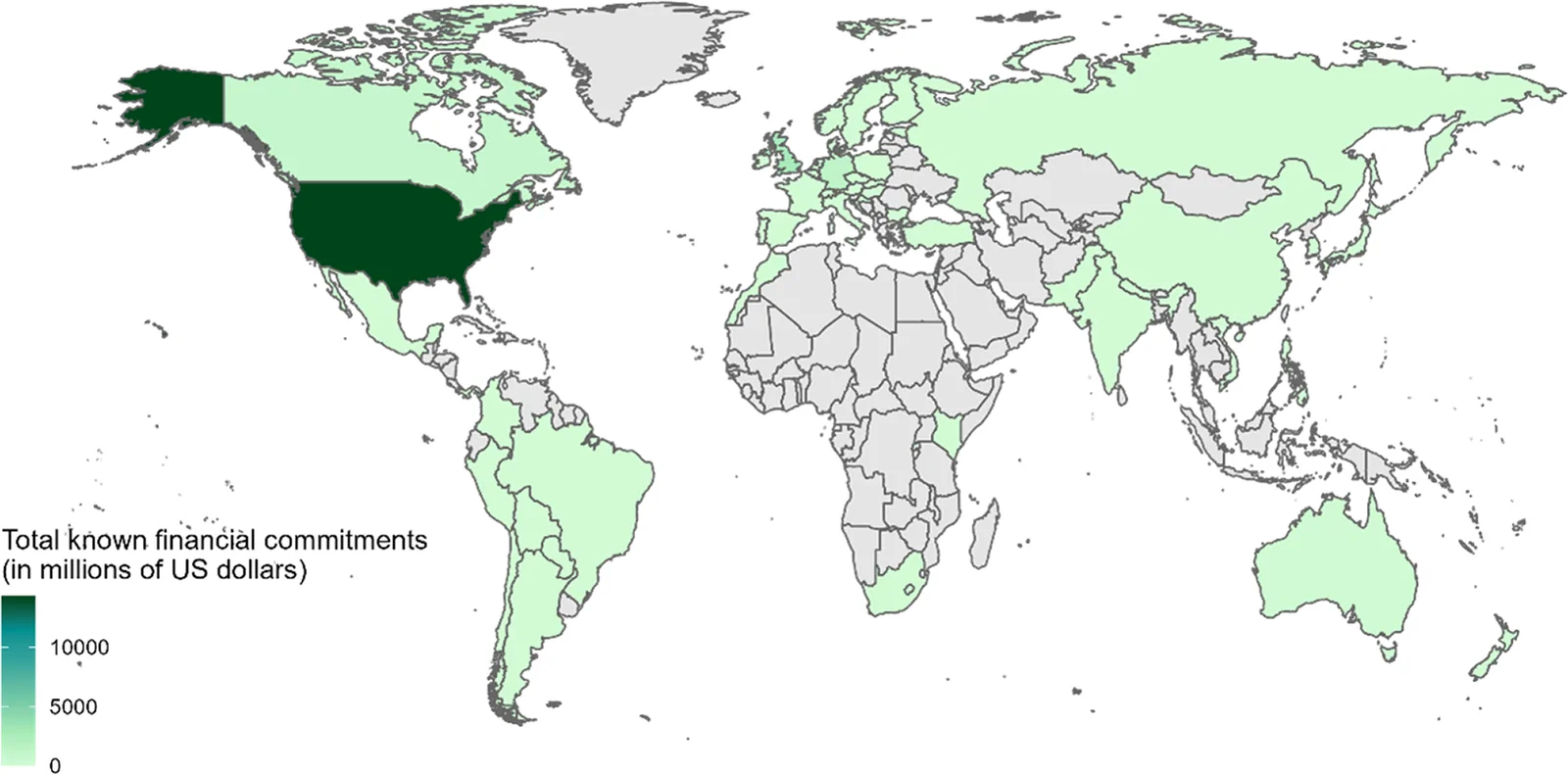
Global distribution of total known financial commitments by funder location. Funding amounts are included only when they have been published by the funder. Data cover the period from 1 January 2020 to 6 December 2025. Funding amounts are presented as absolute values (as recorded in the Pandemic PACT dataset (converted to USD at the time of funding, without adjustment for inflation)). Financial commitment information is available for 76% of the awards in the Pandemic PACT database (n = 21,416/28,176). Although awards may be granted by multiple funding organisations from different countries, this is uncommon, with only 0.2% of awards with known financial commitments involving cross-country funding (n = 44/21,416). In such cases, there is a possibility of double counting, as the same financial commitment may be recorded under multiple countries. In Extended Data 4, we illustrate the global distribution of total awards by funder location available in the Pandemic PACT database.

UK-based institutions funded the second-highest known financial commitment, totalling $2.7 billion, or 10.4% of all known funding commitments in the Pandemic PACT database. Among UK-based funders, the UK Research and Innovation (UKRI) awarded $1.4 billion across 1,600 grants, while the Wellcome Trust awarded $773.5 million across 387 grants. Figure 3 provides a detailed breakdown of the total number of awards and financial commitments for the funding organisations with the highest levels of known funding, according to the data.

**Figure 3.**
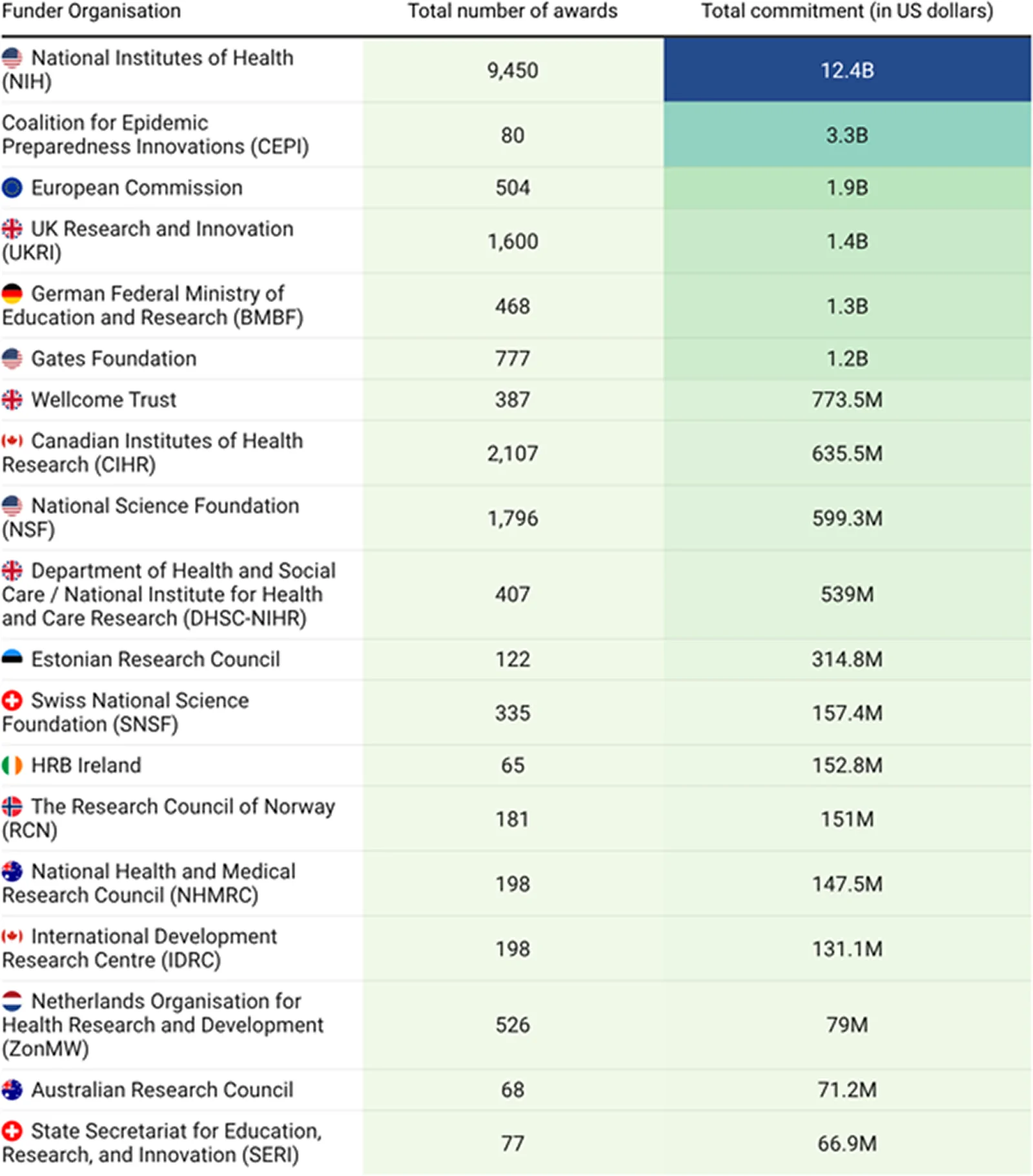
Total number of awards and known financial commitments by funding organisations, sorted by total known financial commitments. The figure shows the twenty funding organisations with the highest funding amounts, according to the Pandemic PACT data. Data cover the period from 1 January 2020 to 6 December 2025. Flags next to funder names represent the location where the funder is based. No flags are used for multinational organisations. Many research funding organisations operate independently. However, funding mechanisms, sources, and flows vary across funders and can be interconnected. The analyses in this paper do not account for relationships between funding organisations unless they are explicitly listed as co-funders. As a result, contributions from some funders to other entities or funding schemes (when not explicitly stated) were not included in the total funding calculations, potentially leading to an underestimation of their contributions in some cases. Funding amounts are presented as absolute values.

Among all 330 funders, the NIH in the US ranks first with a total known financial commitment of $12.4 billion distributed across 9,450 awards, averaging $1.3 million per award, followed by the Coalition for Epidemic Preparedness Innovations (CEPI), which contributed $3.3 billion across 80 awards, with an average of $42.2 million per award. It is acknowledged that there are instances whereby an individual funding organisation may receive funding contributions from other funders also included in the PACT database such as CEPI (who are supported by governments, the European Commission, and philanthropies). Extended Data 1 presents a detailed breakdown of total number of awards and financial commitments, along with the percentage of awards with funding amount information available, for each funding organisation.

### Research institutions

Research funding was awarded to research institutions spread across 130 countries. Among these, 42.7% (n = 12,040/28,176) of the awards available in the Pandemic PACT Tracker were granted to institutions in the US, amounting to $13.9 billion and representing 53.6% of the total known funding commitments. This was followed by research institutions in the UK which received $2.9 billion, allocated across 2,365 awards. The Research Triangle Institute, based in the US, secured the largest known financial investment, totalling $1.3 billion allocated across 30 awards. Ranking second, third, and fourth in total known funding were Leidos Biomedical Research, New York University School of Medicine, and the Fred Hutchinson Cancer Research Center, all of which based in the US. These organisations received total financial commitments of $767 million, $695 million, and $618 million, respectively, distributed across multiple grants covering a wide range of research areas. Figure 4 illustrates the global distribution of total known financial commitments by research institution location.

**Figure 4.**
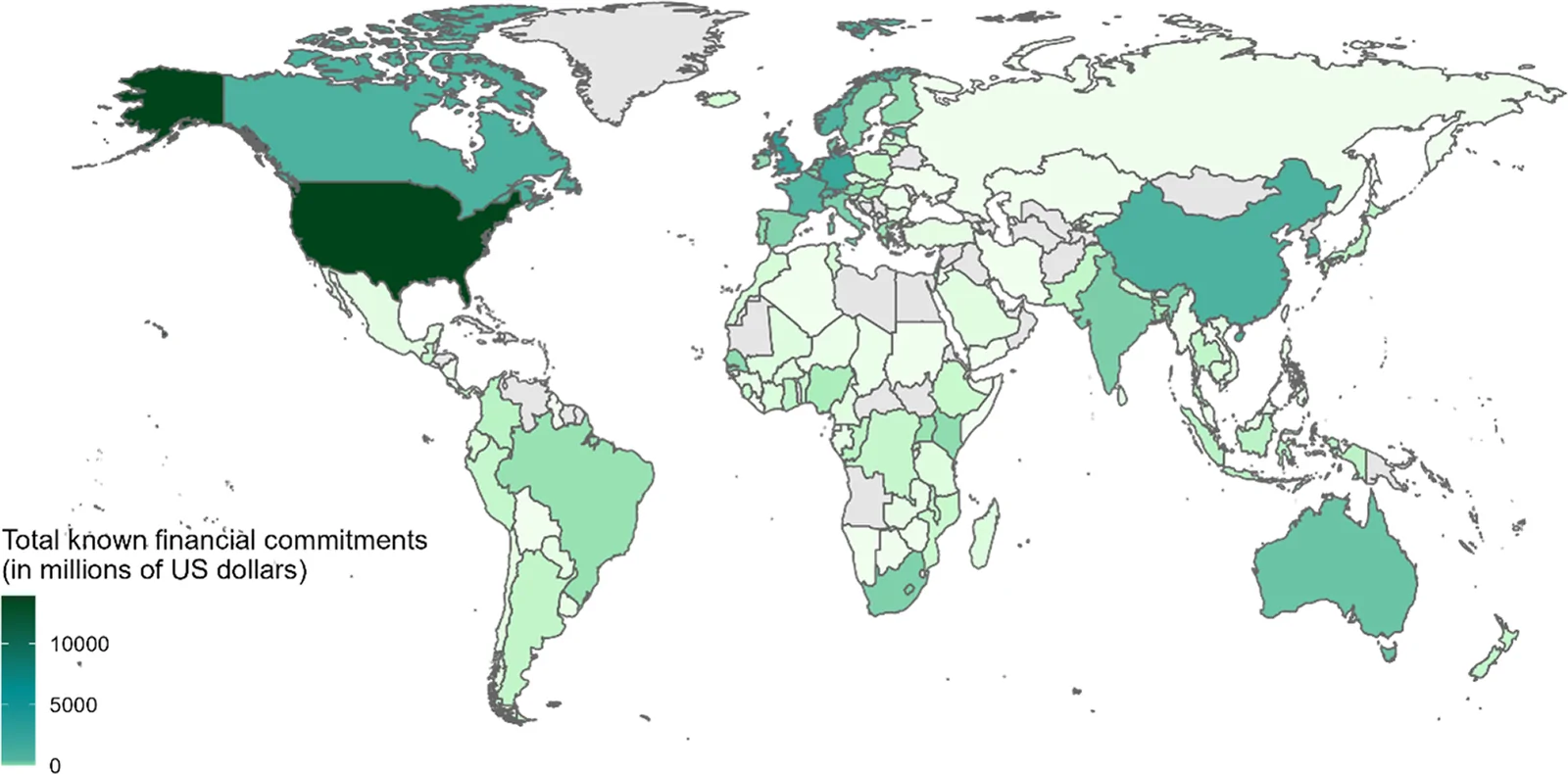
Global distribution of total known financial commitments by research institution location. Funding amounts are included only when they have been published by the funder. Funding amounts are presented as absolute values. Data cover the period from 1 January 2020 to 6 December 2025. Financial commitment information is available for 76% of the awards in the Pandemic PACT database (n = 21,416/28,176). Although awards may be awarded to multiple research institutions from different countries, this is uncommon, with only 0.2% of grants with known financial commitments involving cross-country awarding (n = 44/21,416). In such cases, there is a possibility of double counting, as the same financial commitment may be recorded under multiple countries. In Extended Data 4, we illustrate the global distribution of total awards by research institution location available in the Pandemic PACT database.

The distribution of funded research institutions closely reflects the patterns observed in funding sources. The majority of funding sources originate from organisations exclusively based in high-income countries (HICs) [1], accounting for 91.1% (n = 25,661/28,176) of the total awards and $25.8 billion in investments, which represent 99.7% of the total financial commitments. Relatedly, only 8.8% (n = 2,466/28,176) of the awards were allocated to awards involving at least one research institution based in a LMIC. These investments amounted to $1.5 billion, representing 5.9% of the total known financial commitments. Although financial data completeness was lower for LMIC-led awards than for the dataset overall, the similarly low proportion of awards involving LMIC institutions provides complementary evidence of their relatively limited representation within the PACT dataset. Among LMICs, the largest funding amount was directed to research institutions in China, which received $741 million across 64 grants.

Figure 5 provides a detailed breakdown of the total number of awards and financial commitments for research led by at least one institution based in an LMIC, highlighting the countries with the highest levels of known funding, according to the data, and Figure 6 depicts the flow of financial commitments, contrasting awards led by institutions in LMICs with those entirely led by institutions in HICs. The figure highlights the difference in funding allocation between these groups. For instance, UK-based funder organisations allocated $2.7 billion in total funding, but only 4.8%—equivalent to $130.4 million—was directed toward awards led by at least one institution in an LMIC, reflecting the common requirement of many UK-funded programmes for lead institutions to be UK-based. Similarly, US-based funders invested $14.3 billion in total funding, of which just 2.1%, or $301.1 million, was granted to awards led by at least one LMIC-based institution.

**Figure 5.**
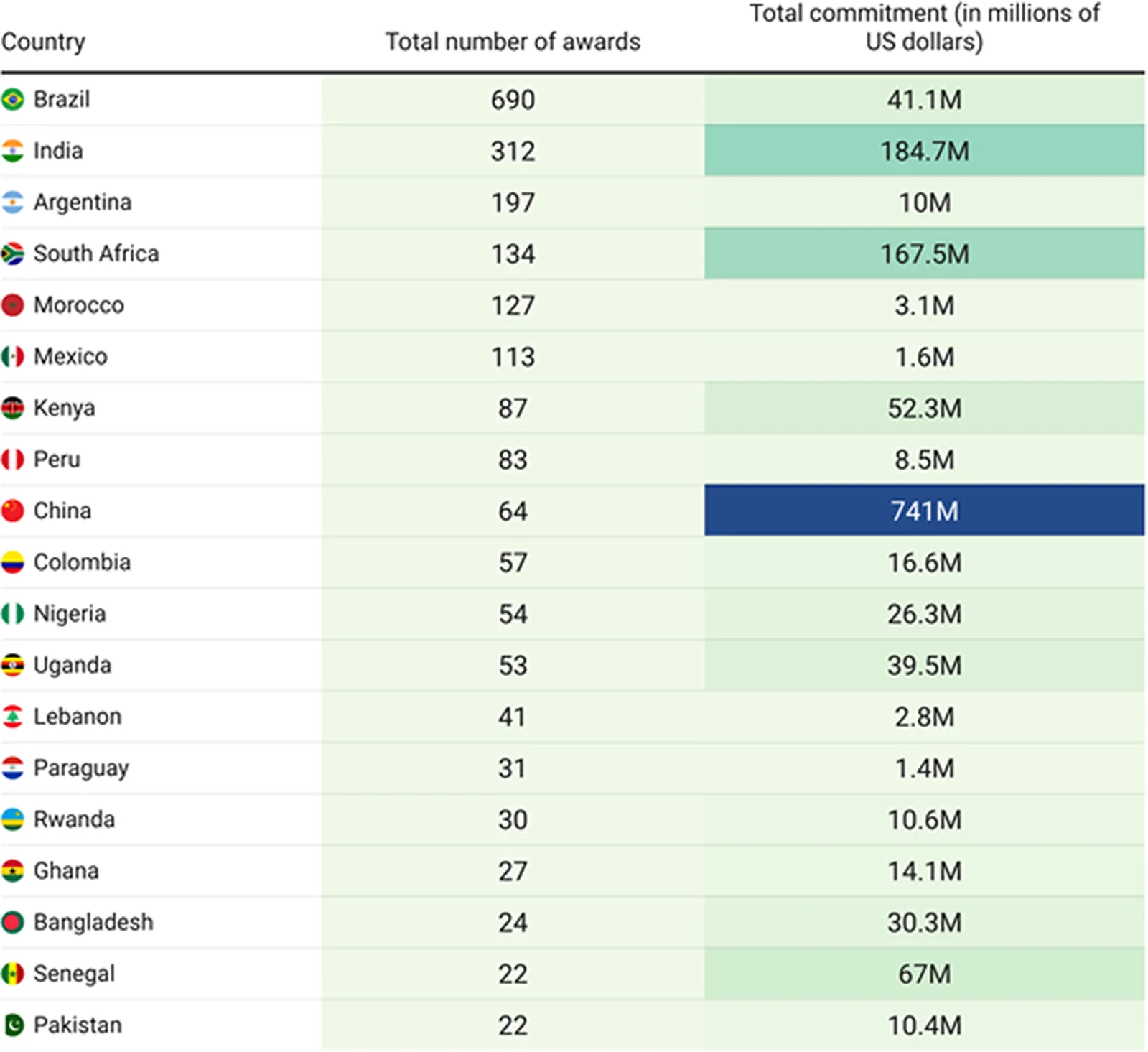
Total number of awards and known financial commitments by research institution countries located in LMICs, sorted by total number of awards. The figure shows the twenty research institution countries located in LMICs with the highest funding amounts, according to the Pandemic PACT data. Data cover the period from 1 January 2020 to 6 December 2025. Awards may be granted to multiple research institutions from different countries, resulting in a total count by country exceeding the overall number of awards and known financial commitments available in Pandemic PACT. Funding amounts are presented as absolute values.

**Figure 6.**
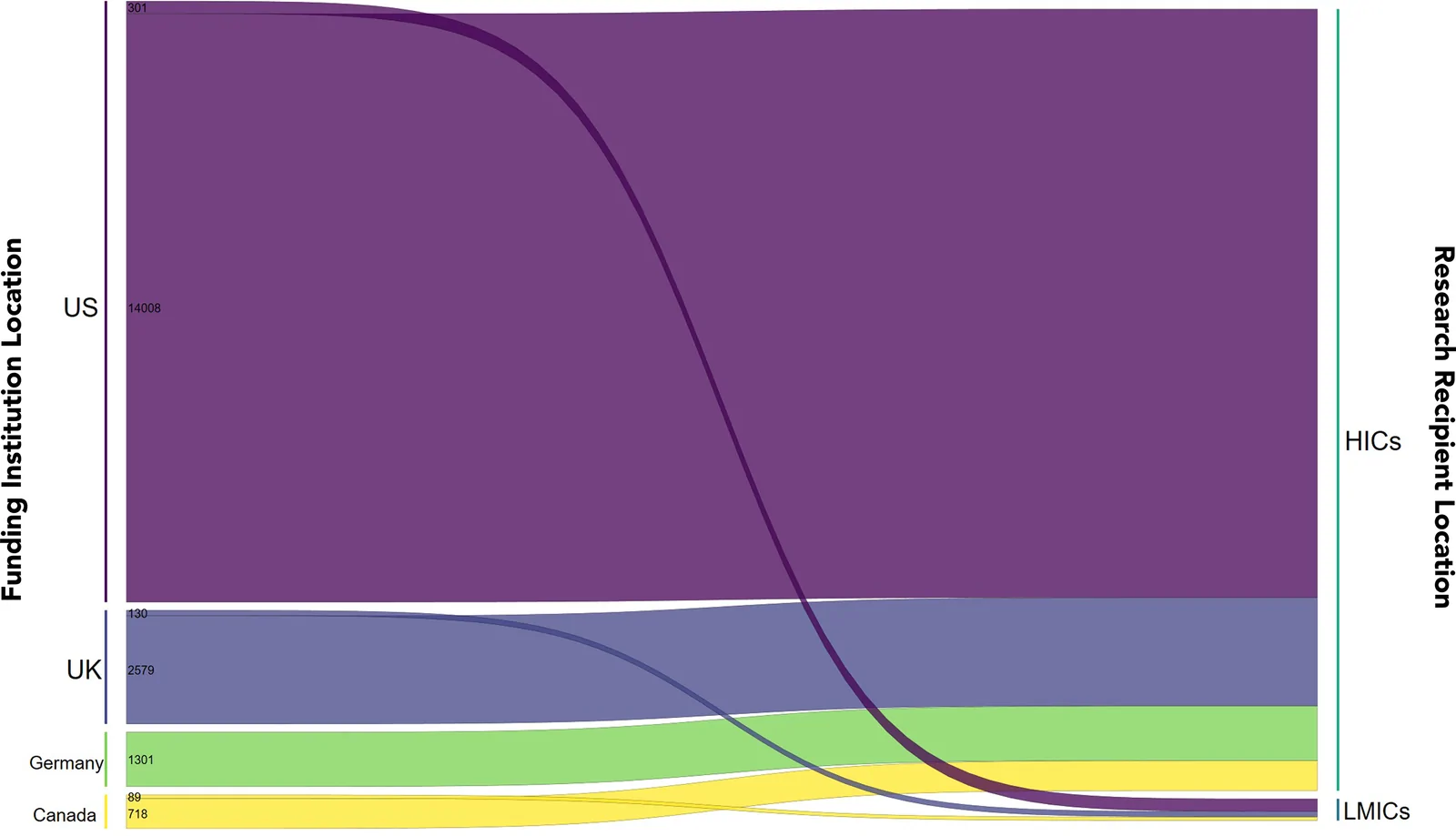
Distribution of known financial commitments (in millions of US dollars) to research institutions in LMICs and HICs by funding organisation country. The figure shows the four countries where funder organisations with the largest financial commitments are based, according to the dataset: UK, US, Germany, and Canada. Awards granted by at least one these countries to awards led by at least one institution based in a LMIC totalled approximately $511 million. This accounts for 34.1% of the total known financial commitments to LMIC-led awards in the dataset, which amount to $1.5 billion. These awards represent 26.6% (n = 656/2,466) of awards awarded to research involving at least one LMIC-based institution. Funding amounts are included only when reported by the funder. Financial commitments information is available for 49.3% of LMIC-led awards in the Pandemic PACT database (n = 1,215/2,466). Although awards may be awarded by multiple funding organisations from different countries, this is uncommon, with only 1.9% of awards led by at least one institution based in a LMIC with known financial commitments involving cross-country funding (n = 23/1,215). In such cases, there is a possibility of double counting, as the same financial commitment may be recorded under multiple countries. Funding amounts are presented as absolute values. Data cover the period from 1 January 2020 to 6 December 2025.

According to the data, South Africa ranked first among all LMICs as recipients of financial commitments from UK-based funders, receiving nearly $37.7 million across 20 awards. Of these, ten were funded by the Wellcome Trust, three by the UKRI, two by the Department of Health and Social Care/National Institute for Health and Care Research (DHSC-NIHR), two co-funded by both DHSC-NIHR and UKRI, two co-funded by DHSC-NIHR and the Royal Society of Tropical Medicine and Hygiene (RSTMH), and one by Cambridge Africa.

US-based funders similarly directed a significant share of their LMIC investments to South Africa, which received approximately $101 million across 58 awards. Of these, 31 awards, amounting to approximately $58 million, were awarded to the Wits Health Consortium, with all funding provided by the Gates Foundation. Other major LMIC recipients of US-based funding spanned Asia, Africa, and Latin America. India received approximately $45 million across 68 awards, followed by Bangladesh with $23 million across 15 awards, and Uganda with $18 million spread over 9 awards. In Nigeria, $16 million was allocated through 24 awards, while Kenya received $13 million across 17 awards. Figure 7 illustrates the distribution of known financial commitments to awards led by institutions in LMICs from UK- and US-based funders, whose countries contributed the largest share of high-income country investments in LMICs. Combined, 2.5% ($431.5 million of $17.0 billion) of their known financial commitments were directed to awards led by at least one institution in an LMIC.

**Figure 7.**
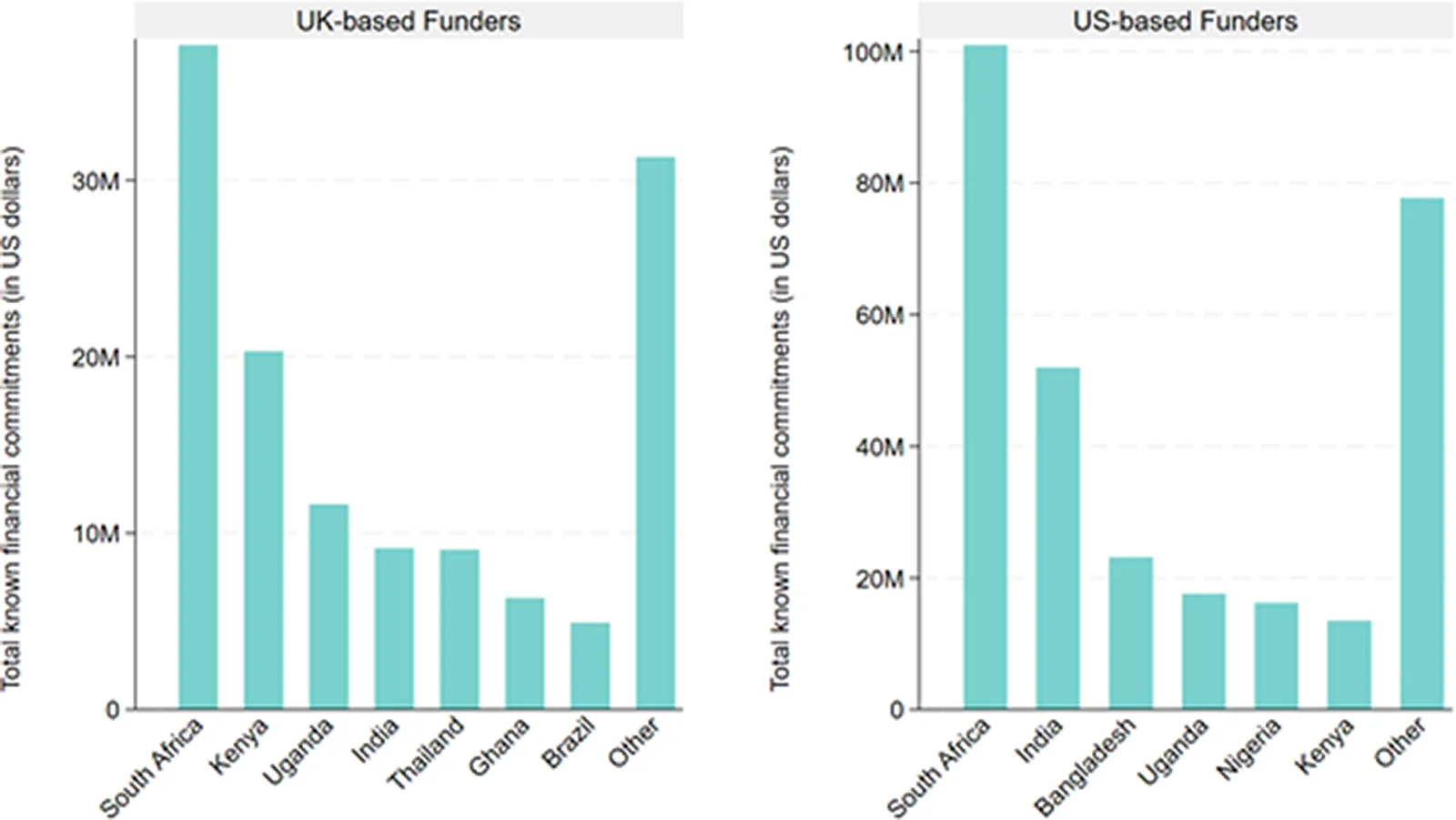
Distribution of known financial commitments (in US dollars) from UK- and US-Based Funders to awards Led by Institutions in LMICs. Funding amounts are included only where they have been publicly reported by the funder. Funding amounts are presented as absolute values. Data cover the period from 1 January 2020 to 6 December 2025. Financial commitment information is available for 64.8 of the awards led by at least one institution based in a LMIC and funded by UK-based organisations (n = 127/196), and for 90.6% of those funded by US-based organisations (n = 269/297). Awards may be awarded to multiple research institutions from different countries, resulting in a total count by country exceeding the overall number of awards and known financial commitments available in Pandemic PACT. Other LMICs funded by UK-based organisations include: Bangladesh, Benin, Bolivia, Brazil, Cambodia, Cameroon, China, Colombia, Democratic Republic of the Congo, Ecuador, Ethiopia, Gambia, Indonesia, Jamaica, Lebanon, Madagascar, Malawi, Malaysia, Mali, Mexico, Mozambique, Nepal, Namibia, Nigeria, Pakistan, Palestine, Peru, Philippines, Rwanda, Senegal, Sierra Leone, Somalia, South Africa, Sri Lanka, Tanzania, Thailand, Uganda, Zambia, and Zimbabwe. Other LMICs funded by US-based organisations include: Argentina, Bangladesh, Botswana, Brazil, Cambodia, Cameroon, Colombia, Congo, Democratic Republic of the Congo, El Salvador, Ethiopia, Ghana, Indonesia, Kazakhstan, Madagascar, Malawi, Mali, Mauritius, Mexico, Mozambique, Nepal, Nicaragua, Nigeria, Pakistan, Peru, Rwanda, Senegal, Sierra Leone, Thailand, Tunisia, Turkey, and Zambia.

Funding from Canadian institutions to LMIC-based institutions was also meaningful. Among Canadian funders, Kenya was the largest LMIC recipient, receiving approximately $10 million across 17 awards. Other key recipients included Uganda, with $9 million across 9 awards; Senegal, which secured $7 million through 13 awards; South Africa, with $7 million across 10 awards, and Peru, which was awarded $5 million through 4 awards. In comparison, Danish funders supported a smaller number of projects involving LMIC-based institutions. A total of fourteen awards were identified, with known financial commitment information available for three. Two of these three awards, each valued at $800,000, were awarded to institutions in Jordan and Tanzania, respectively. Both awards were funded by the Novo Nordisk Foundation.

### Global funding for high-risk infectious disease research

Since some research awards may span multiple pathogen families (7.6% of awards with known financial commitments; n = 1,632/21,416), it is challenging to determine the exact portion of the award directed to each family. To account for this, we present both upper-bound and minimum funding allocation estimates. The upper-bound estimate represent the total amount a specific family could have received if it had been allocated the entirety of the awards, while the minimum values reflect funding for awards exclusively focused on that family. An analysis of the total known funding allocated to high-risk infectious diseases revealed that awards focused on Coronaviridae were particularly prominent. Coronaviridae research received at least $19.3 billion, with a potential total financial commitment nearing $20.6 billion distributed across 23,503 awards. This substantial investment reflects the global impact of the COVID-19 pandemic and the concentrated efforts to address it. Accordingly, the upper-bound allocation estimate to Coronaviridae research, which includes grants exclusively focused on Coronaviridae as well as those addressing multiple pathogen families that include Coronaviridae, accounts for approximately 79.5% of the total commitments available in the Pandemic PACT database. Figure 8 illustrates the distribution of total known financial commitments across pathogen families, highlighting both the potential minimum and maximum amount. The distribution is highly skewed, with Coronaviridae dominating the funding landscape. To put into perspective, the pathogen family with the second-largest known financial commitment is the concept of ‘Unknown’ pathogen, which has received at least $1.1 billion, with potential commitments approaching $1.8 billion. Awards addressing the concept of ‘Unkown’ broadly support research on unidentified or emerging pathogens with pandemic potential. They generally focus on strengthening pandemic preparedness and response, aiming to generate the evidence, tools, and networks that can be rapidly mobilised when a previously uncharacterised pathogen emerges.

**Figure 8.**
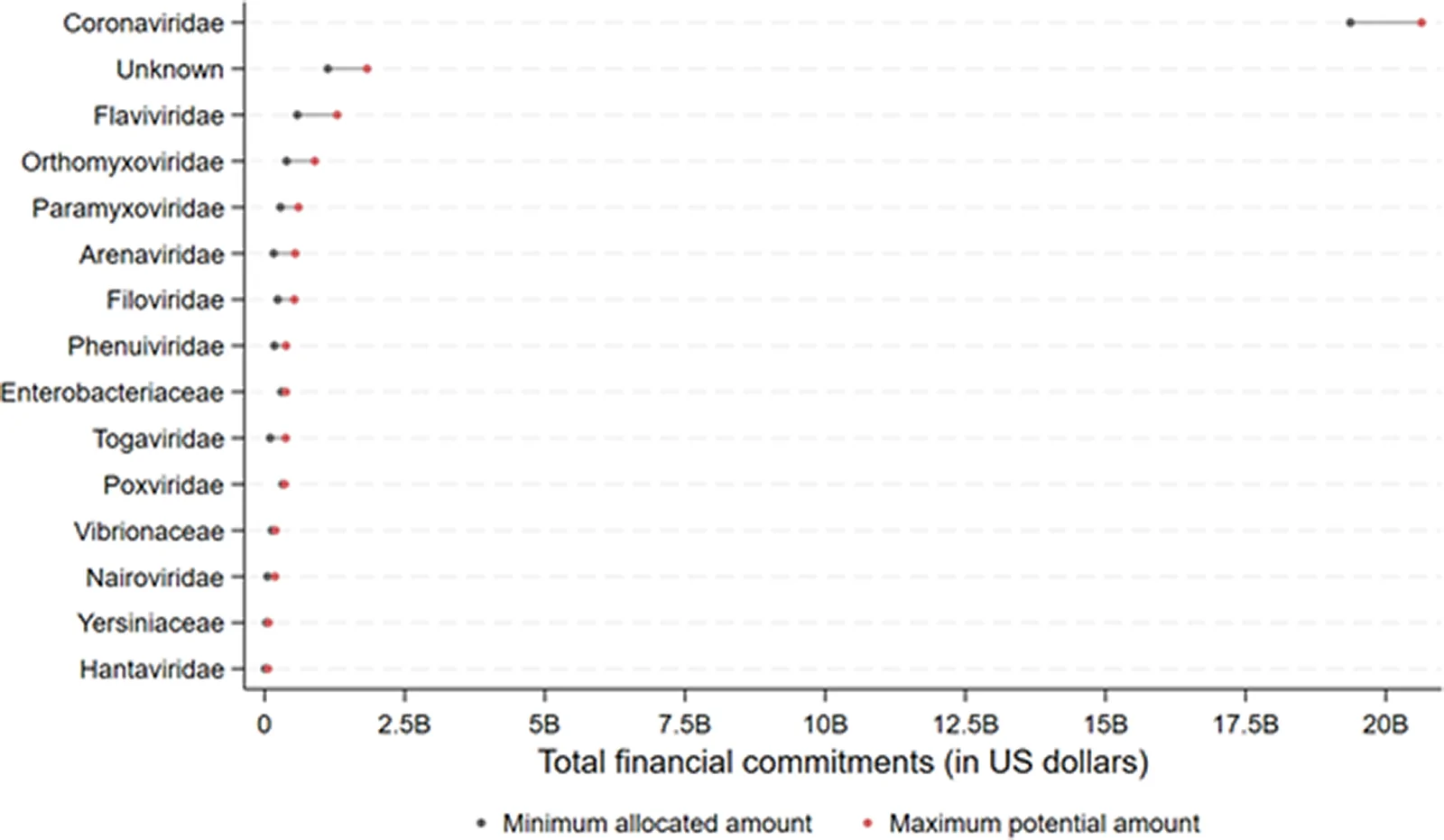
Funding distribution across Pandemic PACT list of pathogen families. Grey dots represent the minimum known funding amount allocated to projects entirely focused on a specific pathogen family. The maximum values indicate the potential upper-bound allocation estimate that a particular family could have received. The red dots represent the maximum total funding that could have been allocated to a family if all awards for projects spanning multiple families had been directed to that family. Data cover the period from 1 January 2020 to 6 December 2025. The “Unknown” pathogen family represents a concept rather than a specific family, capturing the risk posed by currently unidentified pathogens with epidemic or pandemic potential.

When analysing the total known funding allocated across diseases, excluding awards exclusively focused on Coronaviridae, the concept of ‘Unkown’ pathogen received the highest amount of financial commitments, according to the dataset. Following this, research on Flaviviridae, Orthomyxoviridae, and Paramyxoviridae ranked second, third, and fourth, with potential commitments of $1.3 billion, $895.2 million, and $602.6 million, respectively. Figure 9 illustrates the distribution of total known financial commitments across pathogen families, excluding Coronaviridae-focused awards. Notably, Hantaviridae and Yersiniaceae virus disease received the lowest levels of financial commitments, with potential total investments of approximately $59 million and $68.7 million, respectively.

**Figure 9.**
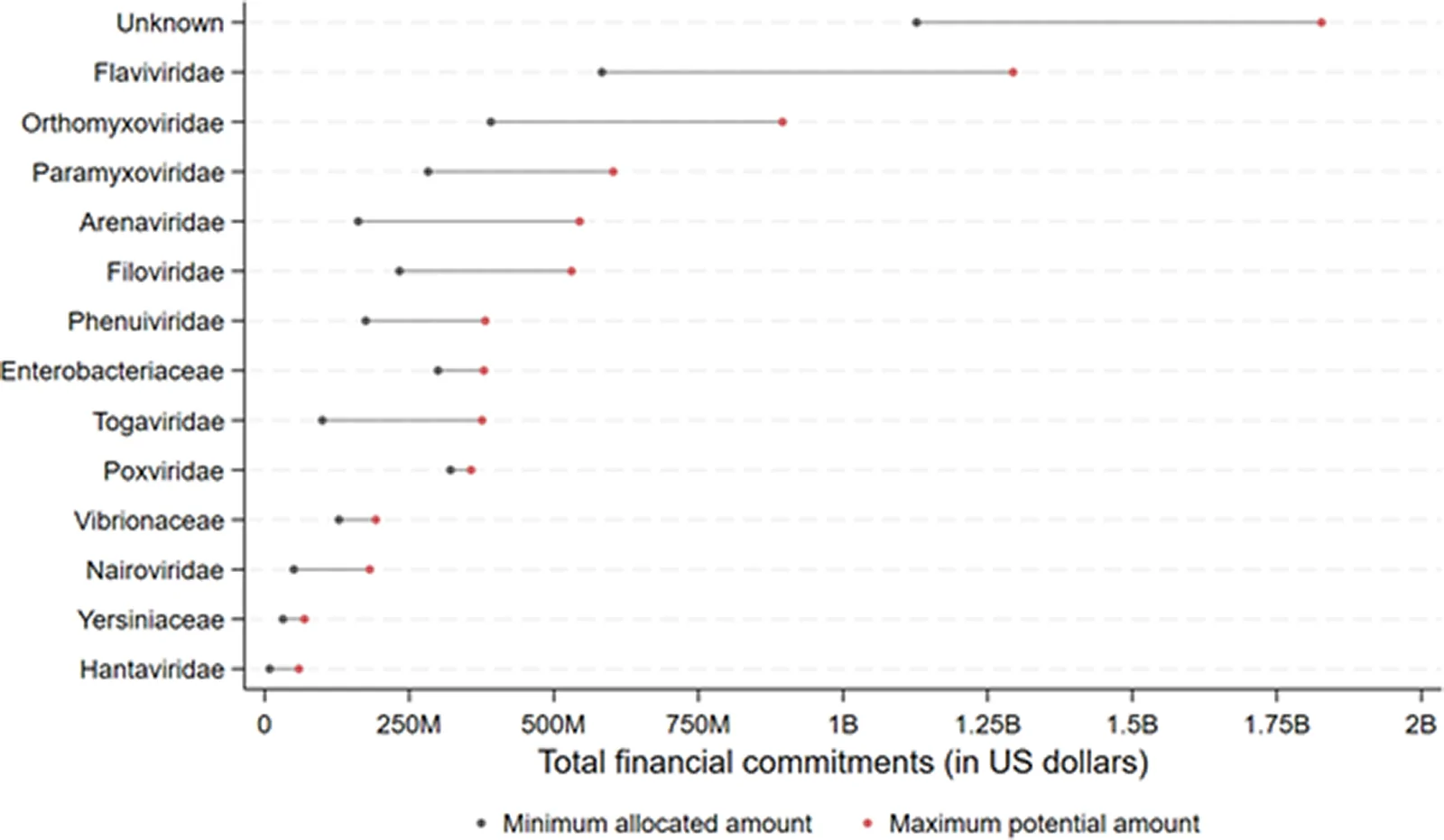
Funding distribution across Pandemic PACT list of pathogen families, excluding awards exclusively focused on Coronaviridae. Grey dots represent the minimum known funding amount allocated to projects entirely focused on a specific pathogen family. The maximum values indicate the upper-bound allocation estimate that a particular family could have received. The red dots represent the potential total funding that could have been allocated to a family if all awards for projects spanning multiple families had been directed to that family. Data cover the period from 1 January 2020 to 6 December 2025.

By presenting both upper-bound and minimum funding allocation estimates for research on high-risk pathogen families, Figure 9 provides additional insight into the extent to which each disease is studied independently versus in combination with others. For example, while the concept of ‘Unkown’ pathogen has received the highest potential financial commitments, there is a substantial gap between its minimum and potential upper-bound funding totals. Specifically, among the 813 awards that include the concept of ‘Unkown’ pathogens, fewer than half (48.8%; n = 397/813) are exclusively focused on it, indicating that most awards study the concept of ‘Unknown’ pathogen with other pathogen families. In contrast, research on Poxviridae is predominantly conducted in isolation: 79.3% (n = 195/246) of awards dedicated to Poxviridae focus solely on this family. Of the $356.8 million in potential funding for Poxviridae research, $321.3 million has been allocated to awards studying Poxviridae alone.

### Trends in global funding (2020–2025) according to grants initiated yearly

Pandemic PACT also tracks the timing of research funding, including the start dates of awards or the publication years of awards. Although it is challenging to precisely determine the yearly distribution of funding within an award’s duration, understanding the start dates of awards provides valuable insights into the timing of investment decisions and the alignment of funding priorities with emerging public health needs. Figure 10 illustrates the evolution of total known financial commitments across diseases grouped by pathogen families, highlighting trends in financial commitments for diseases with the highest potential known investments from 2020 to 2025, revealing distinct trajectories.

**Figure 10.**
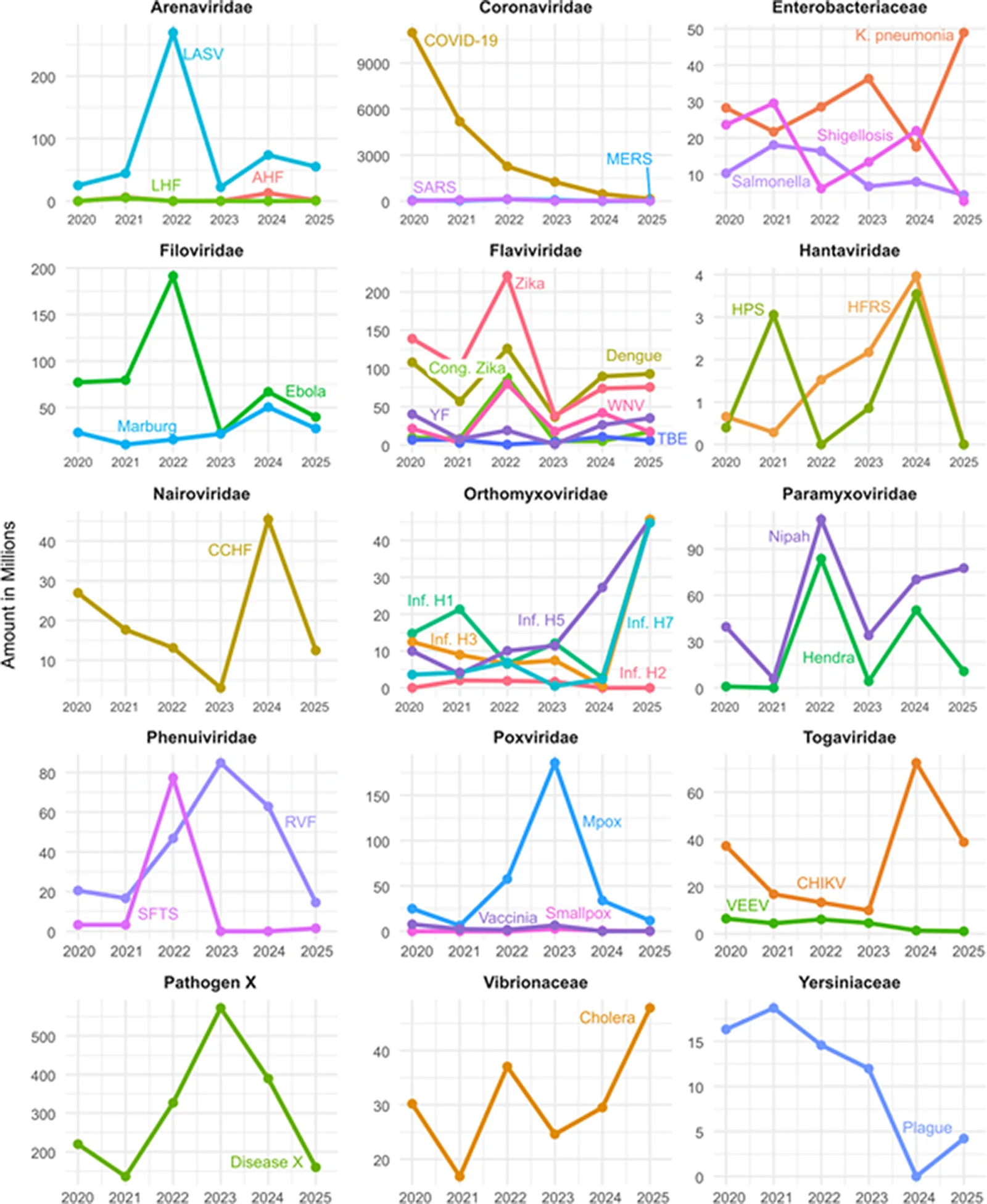
Total know financial commitments across diseases over time, grouped by pathogen families. The figure illustrates the total upper-bound estimated investment in each disease, grouped by pathogen families listed in Pandemic PACT. Although awards may be granted to research awards spanning more than one disease, this is uncommon, with only 9% of awards with known financial commitments involving multiple diseases awards (n = 1,928/21,416). In such cases, there is a possibility of double counting, as the same financial commitment may be recorded under multiple diseases. Due to the highly skewed distribution of funding amounts, where a small number of diseases (particularly COVID-19) received disproportionately large investments, total known financial commitments were log-transformed. Data cover the period from 1 January 2020 to 6 December 2025. Abbreviations: LASV (Lassa Fever Virus); LHF (Lujo Haemorrhagic Fever); AHF (Argentine Haemorrhagic Fever); MERS (Middle Eastern Respiratory Syndrome); SARS (Severe Acute Respiratory Syndrome); K. Pneumonia (Bacterial infection caused by Klebisella Pneumonia); Cong. Zika (Congenital Zika disease); YF (Yellow Fever); TBE (Tick-borne encephalitis); WNV (West Nile Virus); HPS (Haemorrhagic Pulmonary Syndrome); HFRS (Haemorrhagic Fever with Renal Syndrome); CCHF (Crimean-Congo Haemorrhagic Fever); Inf. (Influenza); SFTS (Severe Fever with Thrombocytopenia Syndrome); RVF (Rift Valley Fever); CHIKV (Chikungunya virus); VEEV (Venezuelan equine encephalitis virus).

For instance, funding for the concept of ‘Disease X’ exhibited clear fluctuations. After a potential known investment of $211.7 million in 2020, new commitments dropped to $133.5 million in 2021, then increased to $326.7 million in 2022 and $572.1 million in 2023, before dropping to nearly $389.5 million in 2024 and $159.9 million in 2025. Orthomyxoviridae followed a different pattern, with relatively low yearly initiated new funding, typically between $5 million and $20 million per year, for Influenza caused by Influenza A virus subtypes H1, H2, H3, H5, and H7 between 2020 and 2024. In 2025, funding increased sharply for all subtypes except H2, with each reaching a peak of approximately $46 million. Influenza A virus subtypes H6 and H10 received no funding commitments over the entire period. In contrast, funding for Mpox began at relatively low levels, with $24.9 million in 2020 and $6.3 million in 2021, but grew steadily over time, reaching $185.8 million in 2023 before declining substantially to $11.8 million in 2025. Meanwhile, LASV reached its peak funding in 2022 with $269.6 million in potential commitments, which represents the highest yearly initiated investment among awards for non-COVID-19 and non-Disease X research. However, funding for LASV declined sharply to $22.3 million in 2023, before partially recovering to $73.7 million in 2024 and $55 million in 2025.

These shifting funding trajectories reflect, at least in part, the evolving landscape of infectious disease outbreaks (which provide opportunities for types of research, such as assessment of medical countermeasures, that can only be conducted during an outbreak) and their associated public health burden, although the relationship between funding allocation and disease burden is not always direct. The rapid increase in Mpox funding from 2021 onwards coincides with the escalation of the global Mpox outbreak, which was declared a Public Health Emergency of International Concern (PHEIC) by WHO in 2022. ^22^ Similarly, the sustained relatively high investment in the concept of ‘Disease X’ throughout the period reflects the prominent position that broad pandemic preparedness has occupied on the global health agenda since COVID-19. The steady increase in funding for Rift Valley fever is also noteworthy. Following its classification by WHO in 2024 as a high-priority pathogen with PHEIC potential, ^23^ and the subsequent outbreak in Senegal in 2025, this pattern may suggest that funding allocations can, in some cases, align with emerging epidemiological risks prior to formal emergency declarations.

In contrast, other diseases highlight the limitations in rapidity of reactive funding models. For example, Marburg virus disease received its lowest level of funding in 2024, despite Rwanda declaring its first-ever outbreak in September of that year, an event assessed by WHO as posing very high national risk. ^24^ A similar pattern is observed for Ebola. Despite ongoing outbreaks in the Democratic Republic of the Congo and Uganda, funding levels did not show a corresponding increase in the dataset. For both diseases, research investments responding to these outbreaks are expected to be reflected in subsequent updates to this living review.

### Global funding by high-risk infectious diseases and funding organisations

A total of 47 funding organisations announced financial commitments to research on high-risk pathogens, extending their focus beyond COVID-19. During this period, the NIH reported the highest known financial commitments, amounting to $3.1 billion distributed across 3,804 awards. CEPI and the European Commission followed, with commitments of $1.2 billion across 58 awards and $975.8 million across 231 awards, respectively. Figure 11 provides a detailed breakdown of the total number of grants and known financial commitments for the funding organisations with the highest levels of known funding, according to the data, supporting research on high-risk pathogens during the entire period. Notably, all organisations with the largest funding commitments are based in HICs.

**Figure 11.**
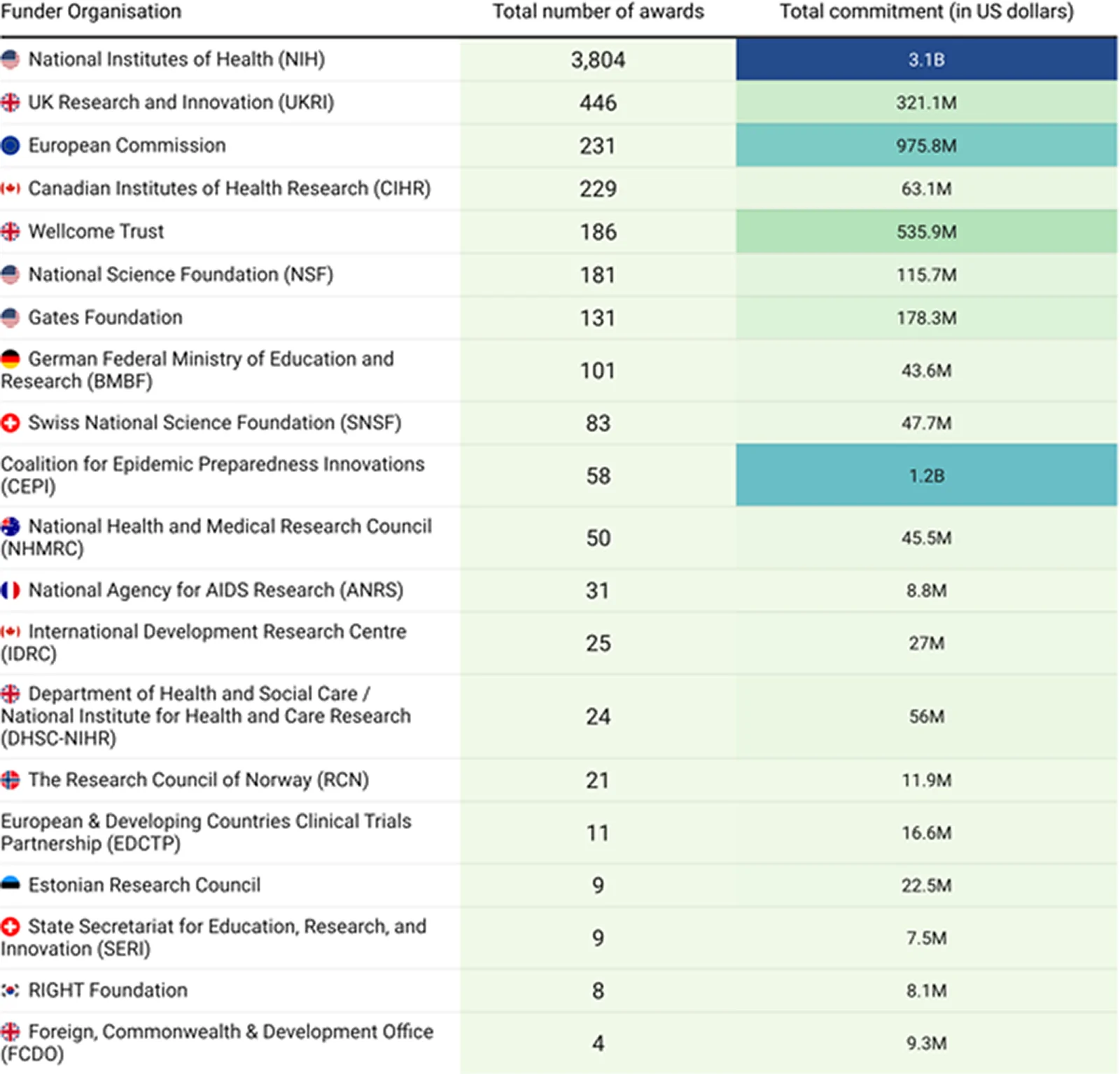
Total number of awards and total known financial commitments by funding organisations supporting research on Pandemic PACT high-risk pathogens (excluding COVID-19), sorted by total number of awards. The figure shows the twenty funding organisations with the highest funding amounts, according to the Pandemic PACT data. Data cover the period from 1 January 2020 to 6 December 2025. Flags next to funder names represent the country where the funder is based. No flags are used for multinational organisations. Many research funding organisations operate independently. However, funding mechanisms, sources, and flows vary across funders and can be interconnected. The analyses in this paper do not account for relationships between funding organisations unless they are explicitly listed as co-funders. As a result, contributions from some funders to other entities or funding schemes (when not explicitly stated) were not included in the total funding calculations, potentially leading to an underestimation of their contributions in some cases.

### Global funding by high-risk infectious diseases and research areas

Apart from awards solely focussed on COVID-19, the distribution of funding across research areas reveals that the largest portion was allocated to vaccine research, development, and implementation, with a potential total financial commitment of $8.3 billion distributed across 2,291 awards to this area. This was followed by funding for research on clinical characterisation and management, which accounted for a potential total financial commitment of $6 billion distributed across 5,641 awards. In contrast, the least-funded research area, according to the data, was on research to inform ethical issues, with $103.5 million in potential financial commitments distributed across 244 awards. It is important to note that differences in the average award amount across research areas, particularly the higher costs associated with vaccine research compared to ethics research, may contribute to these disparities. Figure 12 illustrates the total known financial commitments allocated to research areas across diseases, highlighting the variations in investment focus.

**Figure 12.**
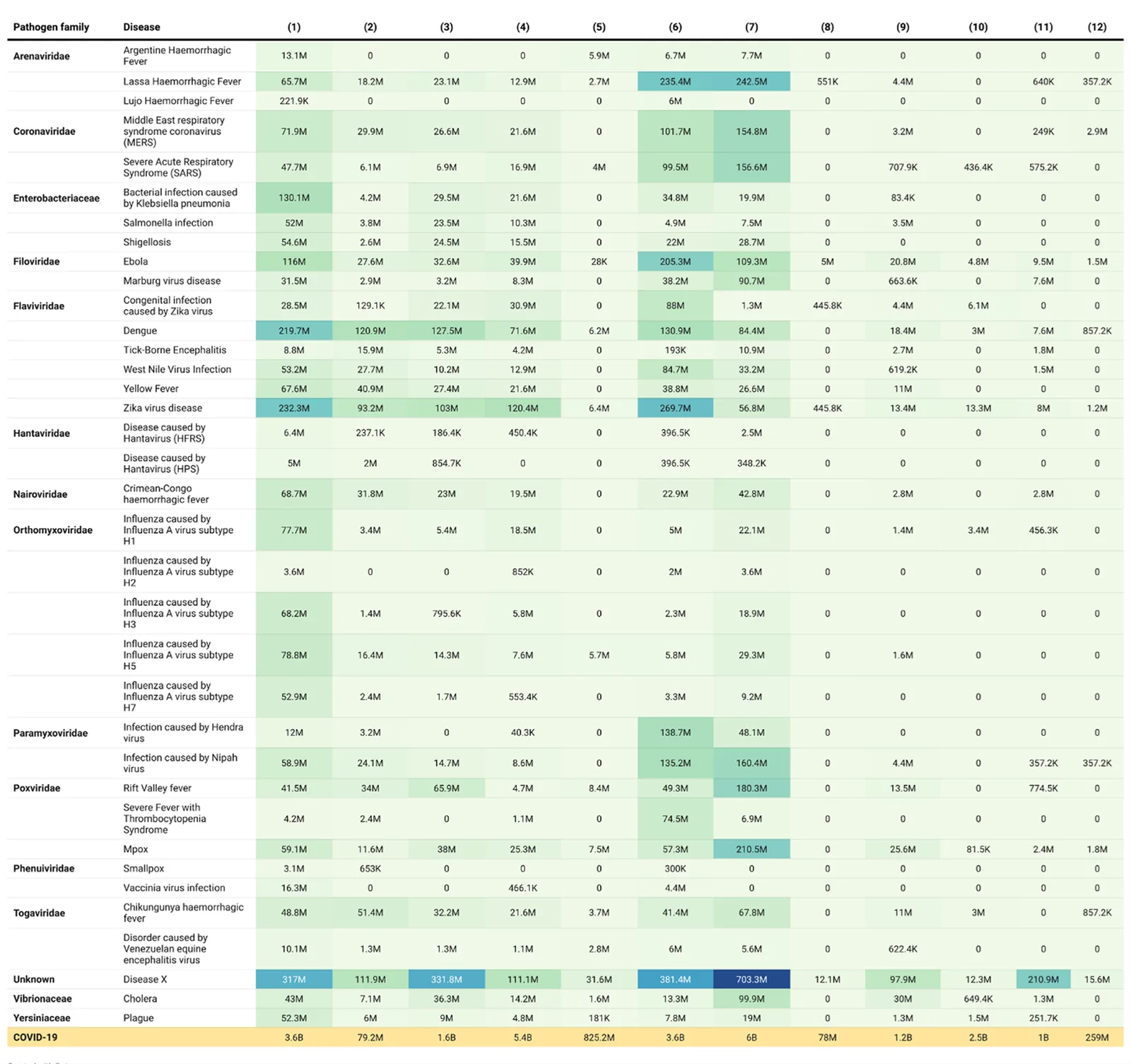
Total known funding distribution across Pandemic PACT list of diseases and research areas. (1) Pathogen: natural history, transmission and diagnostics; (2) Animal and environmental research and research on diseases vectors; (3) Epidemiological studies; (4) Clinical characterisation and management; (5) Infection prevention and control; (6) Therapeutics research, development and implementation; (7) Vaccines research, development and implementation; (8) Research to inform ethical issues; (9) Policies for public health, disease control and community resilience; (10) Secondary impacts of disease, response, and control measures; (11) Health systems research; (12) Research on capacity strengthening. Funding amounts are included only when they have been published by the funder. Financial commitment information is available for 76% of the awards in the Pandemic PACT database (n = 21,416/28,176). Although awards may be granted to awards covering multiple diseases, this is uncommon, with only 9% of awards with known financial commitments involving multiple diseases awards (n = 1,928/21,416). In such cases, there is a possibility of double counting, as the same financial commitment may be recorded under multiple diseases. In Extended Data 4, we illustrate the global distribution of total awards by disease available in the Pandemic PACT database. Data cover the period from 1 January 2020 to 6 December 2025.

Vaccine research stands out as a key investment area, with over $100 million allocated to awards for each of the following diseases since 2020: Ebola, Infection caused by Nipah virus, Lassa Haemorrhagic Fever, MERS, Mpox, Rift Valley fever, and SARS. The concept of ‘Disease X’ also received over $100 million in funding, largely directed towards vaccine preparedness infrastructure, including logistics, supply chains, distribution strategies, trial design, and enabling infrastructure, alongside some pre-clinical research.

Notably, we did not identify in the database award commitments allocated to infection prevention and control research for diseases such as Bacterial infection caused by Klebsiella pneumonia, Congenital infection caused by Zika virus, Crimean-Congo haemorrhagic fever, Disease caused by Hantavirus, Infection caused by Hendra virus, Infection caused by Nipah virus, Influenza caused by Influenza A virus subtypes H1, H2, H3, and H7, Lujo Haemorrhagic Fever, Marburg virus disease, MERS, Salmonella infection, Severe Fever with Thrombocytopenia Syndrome, Shigellosis, Smallpox, Tick-Borne Encephalitis, Vaccinia virus infection, and Yellow Fever.

In the following section, we provide a breakdown of how award commitments have been allocated across research sub-categories and diseases (apart from awards solely focussed on COVID-19) within each of the twelve Pandemic PACT broad research areas. This analysis aims to offer a clearer understanding of where investments have been concentrated, providing an overview of the funding landscape for research on high-risk pathogens.

### Global funding by high-risk infectious diseases and sub-priority research areas

By examining the highest levels of known financial commitments allocated to each research sub-category by disease, we can identify both the priorities that have guided funding decisions and potential gaps in the research landscape. Extended Data 3 provides a comprehensive list of the research areas along with their corresponding sub-categories.

As illustrated in Table 2, we identify, for each broad research area, the specific sub-category that received the greatest share of funding. For example, in the area of “Pathogen: natural history, transmission, and diagnostics,” investment for diseases such as Argentine Haemorrhagic Fever, Lassa Haemorrhagic Fever, Lujo Haemorrhagic Fever, MERS, SARS, Shigellosis, Congenital infection caused by Zika virus, Dengue, West Nile Virus Infection, Yellow Fever, Disease caused by Hantavirus (HPS), Crimean-Congo haemorrhagic fever, Influenza caused by Influenza A virus subtypes H1, H2, H3, H5, and H7, Infection caused by Hendra virus, Infection caused by Nipah virus, and Plague was primarily directed toward immunity research. In contrast, research on the concept of ‘Disease X’ was largely concentrated on diagnostics.

**Table 2.** Total known funding distribution across Pandemic PACT list of diseases and research sub-categories.

Pathogen family	Disease	Pathogen	Animal	Epidemiology	Clinical	IPC	Therapeutics	Vaccines	Ethics	Policy	Secondary Impacts	Health Systems	Capacity Strengthening
**Arenaviridae**	**Argentine Haemorrhagic Fever**	$7.0	$0.0	$0.0	$0.0	$5.9	$6.7	$0.7	$0.0	$0.0	$0.0	$0.0	$0.0
		(Immunity)	—	—	—	(Unspecified)	(Pre-clinical)	(Pre-clinical)	—	—	—	—	—
	**Lassa Haemorrhagic Fever**	$27.7	$17.6	$14.3	$12.9	$2.7	$226.7	$92.1	$0.6	$4.4	$0.0	$0.4	$0.4
		(Immunity)	(Animal source and routes)	(Disease transmission)	(Disease pathogenesis)	(Human-animal interface)	(Pre-clinical)	(Phase 2)	(Research)	(Policy)	—	(Leadership and governance)	(Cross-cutting)
	**Lujo Haemorrhagic Fever**	$0.2	$0.0	$0.0	$0.0	$0.0	$6.0	$0.0	$0.0	$0.0	$0.0	$0.0	$0.0
		(Pathogen morphology)	—	—	—	—	(Pre-clinical)	—	—	—	—	—	—
**Coronaviridae**	**Middle East respiratory syndrome coronavirus (MERS)**	$33.4	$29.9	$24.3	$16.9	$0.0	$100.8	$42.0	$0.0	$2.9	$0.0	$0.2	$2.9
		(Immunity)	(Animal source and routes)	(Disease transmission)	(Disease pathogenesis)	—	(Pre-clinical)	(Phase 1)	—	(Communication)	—	(Health workforce)	(Individual level)
	**Severe Acute Respiratory Syndrome (SARS)**	$26.5	$5.8	$3.8	$11.4	$4.0	$98.1	$73.0	$0.0	$0.7	$0.3	$0.3	$0.0
		(Immunity)	(Animal source and routes)	(Control measures)	(Prognostic factors)	(Restriction measures)	(Pre-clinical)	(Phase 1)	—	(Policy)	(Health)	(Leadership and governance)	—
**Enterobacteriaceae**	**Bacterial infection caused by Klebsiella pneumonia**	$73.4	$1.6	$24.5	$11.0	$0.0	$28.4	$17.1	$0.0	$0.1	$0.0	$0.0	$0.0
		(Pathogen genomics)	(Animal source and routes)	(Disease surveillance)	(Disease pathogenesis)	—	(Pre-clinical)	(Pre-clinical)	—	(Public health interventions)	—	—	—
	**Salmonella infection**	$27.6	$1.2	$20.3	$10.1	$0.0	$4.8	$3.7	$0.0	$3.2	$0.0	$0.0	$0.0
		(Pathogen morphology)	(Animal source and routes)	(Disease surveillance)	(Disease pathogenesis)	—	(Pre-clinical)	(Trial design and infrastructure)	—	(Policy)	—	—	—
	**Shigellosis**	$31.6	$2.6	$24.5	$15.1	$0.0	$19.3	$12.1	$0.0	$0.0	$0.0	$0.0	$0.0
		(Immunity)	(Unspecified)	(Disease surveillance)	(Disease pathogenesis)	—	(Pre-clinical)	(Pre-clinical)	—	—	—	—	—
**Filoviridae**	**Ebola**	$59.5	$26.9	$21.4	$28.1	$0.0	$196.3	$66.7	$4.5	$9.8	$2.0	$8.3	$0.6
		(Pathogen morphology)	(Animal source and routes)	(Disease transmission)	(Disease pathogenesis)	—	(Pre-clinical)	(Design and administration)	(Governance)	(Community engagement)	(Other)	(Leadership and governance)	(Cross-cutting)
	**Marburg virus disease**	$20.0	$2.4	$1.6	$7.6	$0.0	$34.0	$61.0	$0.0	$0.7	$0.0	$7.6	$0.0
		(Pathogen morphology)	(Animal source and routes)	(Disease transmission)	(Supportive care/Clinical management)	—	(Pre-clinical)	(Design and administration)	—	(Communication)	—	(Service delivery/Leadership and governance)	—
**Flaviviridae**	**Congenital infection caused by Zika virus**	$20.5	$0.1	$22.1	$29.6	$0.0	$87.0	$0.9	$0.4	$4.4	$6.1	$0.0	$0.0
		(Immunity)	(Vector biology)	(Disease surveillance)	(Disease pathogenesis)	—	(Pre-clinical)	(Design and administration)	(Social determinants)	(Policy)	(Health)	—	—
	**Dengue**	$111.2	$65.3	$100.8	$46.1	$5.0	$123.3	$39.3	$0.0	$10.1	$3.0	$7.6	$0.9
		(Immunity)	(Vector control)	(Disease surveillance)	(Disease pathogenesis)	(Control measures)	(Pre-clinical)	(Pre-clinical)	—	(Public health interventions)	(Health)	(Service delivery/Leadership and governance)	(Individual level/Institutional level)
	**Tick-Borne Encephalitis**	$7.0	$10.5	$4.5	$3.7	$0.0	$0.2	$8.8	$0.0	$2.7	$0.0	$1.8	$0.0
		(Pathogen morphology)	(Vector control)	(Disease transmission)	(Disease pathogenesis)	—	(Pre-clinical)	(Design and administration)	—	(Policy)	—	(Information systems)	—
	**West Nile Virus Infection**	$34.3	$23.2	$7.0	$10.8	$0.0	$81.1	$28.9	$0.0	$0.6	$0.0	$1.5	$0.0
		(Immunity)	(Vector biology)	(Disease transmission)	(Disease pathogenesis)	—	(Pre-clinical)	(Pre-clinical)	—	(Policy)	—	(Information systems)	—
	**Yellow Fever**	$37.5	$23.4	$16.3	$11.9	$0.0	$30.3	$22.3	$0.0	$3.9	$0.0	$0.0	$0.0
		(Immunity)	(Vector biology)	(Disease surveillance)	(Disease pathogenesis)	—	(Pre-clinical)	(Pre-clinical)	—	(Public health interventions)	—	—	—
	**Zika virus disease**	$136.1	$42.7	$76.6	$93.0	$6.4	$261.0	$43.4	$0.4	$7.9	$9.3	$8.0	$0.9
		(Pathogen morphology)	(Vector control)	(Disease surveillance)	(Disease pathogenesis)	(Control measures)	(Pre-clinical)	(Pre-clinical)	(Social determinants)	(Public health interventions)	(Health)	(Leadership and governance)	(Individual level/Institutional level)
**Hantaviridae**	**Disease caused by Hantavirus (HFRS)**	$5.7	$0.2	$0.2	$0.5	$0.0	$0.4	$2.5	$0.0	$0.0	$0.0	$0.0	$0.0
		(Pathogen morphology)	(Animal source and routes)	(Disease susceptibility)	(Disease pathogenesis)	—	(Pre-clinical)	(Pre-clinical)	—	—	—	—	—
	**Disease caused by Hantavirus (HPS)**	$4.6	$2.0	$0.9	$0.0	$0.0	$0.4	$0.3	$0.0	$0.0	$0.0	$0.0	$0.0
		(Pathogen morphology)	(Animal source and routes)	(Disease surveillance)	—	—	(Pre-clinical)	(Pre-clinical)	—	—	—	—	—
**Nairoviridae**	**Crimean-Congo haemorrhagic fever**	$47.5	$17.4	$16.2	$11.2	$0.0	$14.2	$36.9	$0.0	$2.7	$0.0	$2.0	$0.0
		(Immunity)	(Vector control)	(Disease surveillance)	(Prognostic factors)	—	(Pre-clinical)	(Pre-clinical)	—	(Policy)	—	(Technologies)	—
**Orthomyxoviridae**	**Influenza caused by Influenza A virus subtype H1**	$66.5	$3.4	$3.6	$17.5	$0.0	$5.0	$16.7	$0.0	$0.9	$1.7	$0.5	$0.0
		(Immunity)	(Animal source and routes)	(Disease surveillance)	(Disease pathogenesis)	—	(Pre-clinical)	(Pre-clinical)	—	(Policy)	(Economic)	(Service delivery)	—
	**Influenza caused by Influenza A virus subtype H2**	$3.6	$0.0	$0.0	$0.9	$0.0	$2.0	$3.6	$0.0	$0.0	$0.0	$0.0	$0.0
		(Immunity)	—	—	(Disease pathogenesis)	—	(Pre-clinical)	(Pre-clinical)	—	—	—	—	—
	**Influenza caused by Influenza A virus subtype H3**	$57.7	$1.4	$0.8	$5.5	$0.0	$2.3	$8.0	$0.0	$0.0	$0.0	$0.0	$0.0
		(Immunity)	(Animal source and routes)	(Disease transmission)	(Disease pathogenesis)	—	(Pre-clinical)	(Phase 1)	—	—	—	—	—
	**Influenza caused by Influenza A virus subtype H5**	$59.2	$16.2	$11.7	$4.0	$4.4	$5.5	$15.1	$0.0	$0.9	$0.0	$0.0	$0.0
		(Immunity)	(Animal source and routes)	(Disease surveillance)	(Disease pathogenesis)	(Human-animal interface)	(Pre-clinical)	(Pre-clinical)	—	(Public health interventions)	—	—	—
	**Influenza caused by Influenza A virus subtype H7**	$49.1	$2.4	$1.7	$0.4	$0.0	$3.0	$6.6	$0.0	$0.0	$0.0	$0.0	$0.0
		(Immunity)	(Animal source and routes)	(Disease transmission/Disease surveillance)	(Disease pathogenesis)	—	(Pre-clinical)	(Pre-clinical)	—	—	—	—	—
**Paramyxoviridae**	**Infection caused by Hendra virus**	$12.0	$3.2	$0.0	$0.0	$0.0	$138.7	$48.1	$0.0	$0.0	$0.0	$0.0	$0.0
		(Immunity)	(Animal source and routes/Vector biology)	—	—	—	(Pre-clinical)	(Pre-clinical)	—	—	—	—	—
	**Infection caused by Nipah virus**	$35.7	$24.0	$9.2	$8.6	$0.0	$132.4	$85.8	$0.0	$4.0	$0.0	$0.4	$0.4
		(Immunity)	(Animal source and routes)	(Control measures)	(Disease pathogenesis)	—	(Pre-clinical)	(Phase 1)	—	(Treatment hesitancy)	—	(Service delivery)	(Cross-cutting)
**Poxviridae**	**Rift Valley fever**	$29.3	$31.7	$63.7	$4.6	$0.6	$45.4	$117.8	$0.0	$7.0	$0.0	$0.8	$0.0
		(Pathogen morphology)	(Animal source and routes)	(Disease surveillance)	(Disease pathogenesis)	(Restriction measures)	(Pre-clinical)	(Phase 2)	—	(Public health interventions)	—	(Information systems)	—
	**Severe Fever with Thrombocytopenia Syndrome**	$3.8	$2.4	$0.0	$1.1	$0.0	$74.5	$6.9	$0.0	$0.0	$0.0	$0.0	$0.0
		(Pathogen morphology)	(Vector biology)	—	(Disease pathogenesis)	—	(Pre-clinical)	(Pre-clinical)	—	—	—	—	—
	**Mpox**	$38.0	$9.1	$24.0	$20.9	$3.4	$42.6	$180.6	$0.0	$18.3	$0.1	$1.9	$1.6
		(Pathogen morphology)	(Animal source and routes)	(Disease surveillance)	(Disease pathogenesis)	(Human-animal interface)	(Clinical trial [unspecified phase])	(Phase 2)	—	(Communication)	(Health/Social/Economic)	(Leadership and governance)	(Individual level)
**Phenuiviridae**	**Smallpox**	$3.1	$0.7	$0.0	$0.0	$0.0	$0.3	$0.0	$0.0	$0.0	$0.0	$0.0	$0.0
		(Pathogen morphology)	(Vector biology)	—	—	—	(Pre-clinical)	—	—	—	—	—	—
	**Vaccinia virus infection**	$14.9	$0.0	$0.0	$0.5	$0.0	$4.4	$0.0	$0.0	$0.0	$0.0	$0.0	$0.0
		(Pathogen morphology)	—	—	(Disease pathogenesis)	—	(Pre-clinical)	—	—	—	—	—	—
**Togaviridae**	**Chikungunya haemorrhagic fever**	$29.8	$31.0	$19.5	$11.1	$3.7	$39.6	$65.9	$0.0	$6.9	$3.0	$0.0	$0.9
		(Pathogen genomics)	(Vector control)	(Disease surveillance)	(Disease pathogenesis)	(Control measures)	(Pre-clinical)	(Clinical trial [unspecified phase])	—	(Public health interventions/Community engagement)	(Health/Social)	—	(Individual level/Institutional level)
	**Disorder caused by Venezuelan equine encephalitis virus**	$8.1	$1.3	$1.3	$0.7	$0.0	$6.0	$4.3	$0.0	$0.6	$0.0	$0.0	$0.0
		(Pathogen morphology)	(Animal source and routes)	(Disease surveillance)	(Disease pathogenesis)	—	(Pre-clinical)	(Induced immunity)	—	(Community engagement)	—	—	—
**Unknown**	**Disease X**	$112.5	$79.4	$205.6	$44.4	$15.4	$166.2	$95.1	$10.7	$57.2	$9.7	$162.0	$11.4
		(Diagnostics)	(Animal source and routes)	(Disease surveillance)	(Disease pathogenesis)	(Control measures)	(Pre-clinical)	(Logistics)	(Clinical and Health System Decision-Making)	(Policy)	(Other)	(Leadership and governance)	(Institutional level)
**Vibrionaceae**	**Cholera**	$21.0	$4.3	$31.9	$5.2	$0.5	$8.2	$25.3	$0.0	$14.8	$0.0	$1.3	$0.0
		(Pathogen morphology)	(Vector biology)	(Disease surveillance)	(Clinical management)	(Restriction measures)	(Pre-clinical)	(Induced immunity)	—	(Treatment hesitancy)	—	(Leadership and governance)	—
**Yersiniaceae**	**Plague**	$21.4	$6.0	$5.5	$4.7	$0.2	$1.1	$5.6	$0.0	$0.4	$1.5	$0.3	$0.0
		(Immunity)	(Vector biology)	(Disease surveillance)	(Disease pathogenesis)	(Restriction measures)	(Pre-clinical)	(Pre-clinical)	—	(Policy)	(Unspecified)	(Technologies)	—

Funding amounts are included only when they have been published by the funder. Data cover the period from 1 January 2020 to 6 December 2025. Financial commitment information is available for 76% of the awards in the Pandemic PACT database (n = 21,416/28,176). Although awards may be granted to awards covering multiple diseases, this is uncommon, with only 9% of awards with known financial commitments involving funding for awards covering more than one disease (n = 1,928/21,416). In such cases, there is a possibility of double counting, as the same financial commitment may be recorded under multiple diseases.

For research on therapeutics, pre-clinical studies emerged as the sub-category that received the most funding for nearly all diseases, except for Pandemic-prone influenza and Mpox, where the dominant investment was in clinical trials with unspecified phase. A similar pattern can be observed in vaccine research, where pre-clinical studies generally received the largest share of funding, though this was not consistent across all diseases. For instance, research on Lassa Haemorrhagic Fever, Mpox, and Rift Valley Fever focussed more on Phase 2 clinical trials, while funding for MERS, SARS, Influenza caused by Influenza A virus subtype H3, and Infection caused by Nipah virus was largely concentrated on both Phase 1 trials.

Overall, across the twelve broad research areas tracked by Pandemic PACT, vaccine research received the highest level of potential financial commitment ($8.3 billion), research on clinical characterisation and management ($6 billion), pathogen-related research ($5.3 billion), and therapeutics ($4.6 billion). The Pandemic PACT data indicate that financial commitments were primarily concentrated in early-phase and pre-clinical studies. Notably, Disease X attracted substantial investment across nearly all research domains, with especially high allocations in health systems research relative to the other diseases included in the database. In contrast, funding for several diseases was concentrated within a relatively small number of research areas. Argentine Haemorrhagic Fever, Lujo Haemorrhagic Fever, Shigellosis, HFRS, HPS, Influenza caused by Influenza A virus subtypes H2, H3, and H7, Infection caused by Hendra virus, Severe Fever with Thrombocytopenia Syndrome, Smallpox, and Vaccinia virus infection had no funding allocated to at least half of the twelve broad research areas, indicating comparatively narrow research funding portfolios. Other research areas, such as epidemiology ($2.4 billion) and public health policy and community resilience ($1.4 billion), displayed relative variability in funding by disease and sub-category, with several diseases receiving no known commitments in specific areas. Overall, the data reflect a concentration of resources in certain diseases and clinical trial phases, revealing important gaps, particularly in later-stage trials, health systems, ethics, and capacity-building efforts. Extended Data 5 provides a detailed breakdown of known funding distributions across the Pandemic PACT list of diseases by research sub-category.

### Study populations in high-risk infectious diseases research

The Pandemic PACT database provides relevant information on the study populations included in the research funded by the awards. The database categorises study subjects into seven groups: animals, bacteria, humans, disease vectors, viruses, environment, and other specified populations not listed. Some awards include multiple study populations, such as examining both human populations and viruses simultaneously.

Apart from awards exclusively focused on COVID-19, across all diseases in the Pandemic PACT database, less than 30% of awards involve human populations as study subjects, with the majority focussing on other populations, including animals, viruses, disease vectors, or have an unspecified study population. The only exceptions are Tick-Borne Encephalitis, Mpox, Yellow Fever, Crimean-Congo haemorrhagic fever, Congenital infection caused by Zika virus, Dengue, and Chikungunya, with more than 30% of awards for these disease involving human populations as one of the study subjects. Figure 13 provides a detailed view of the total number of awards in the database allocated across different study populations by disease, alongside the proportional breakdown of the total number of awards. Among the diseases, Argentine Haemorrhagic Fever, Severe Fever with Thrombocytopenia Syndrome, HPS, Influenza caused by Influenza A virus subtype H2, Lujo Haemorrhagic Fever, Smallpox, and Vaccinia virus infection have no awards covering human populations as study subjects. Additionally, the concept of ‘Disease X’ has the highest proportion of awards with unspecified study populations, accounting for 17% of the total awards allocated to this disease.

**Figure 13.**
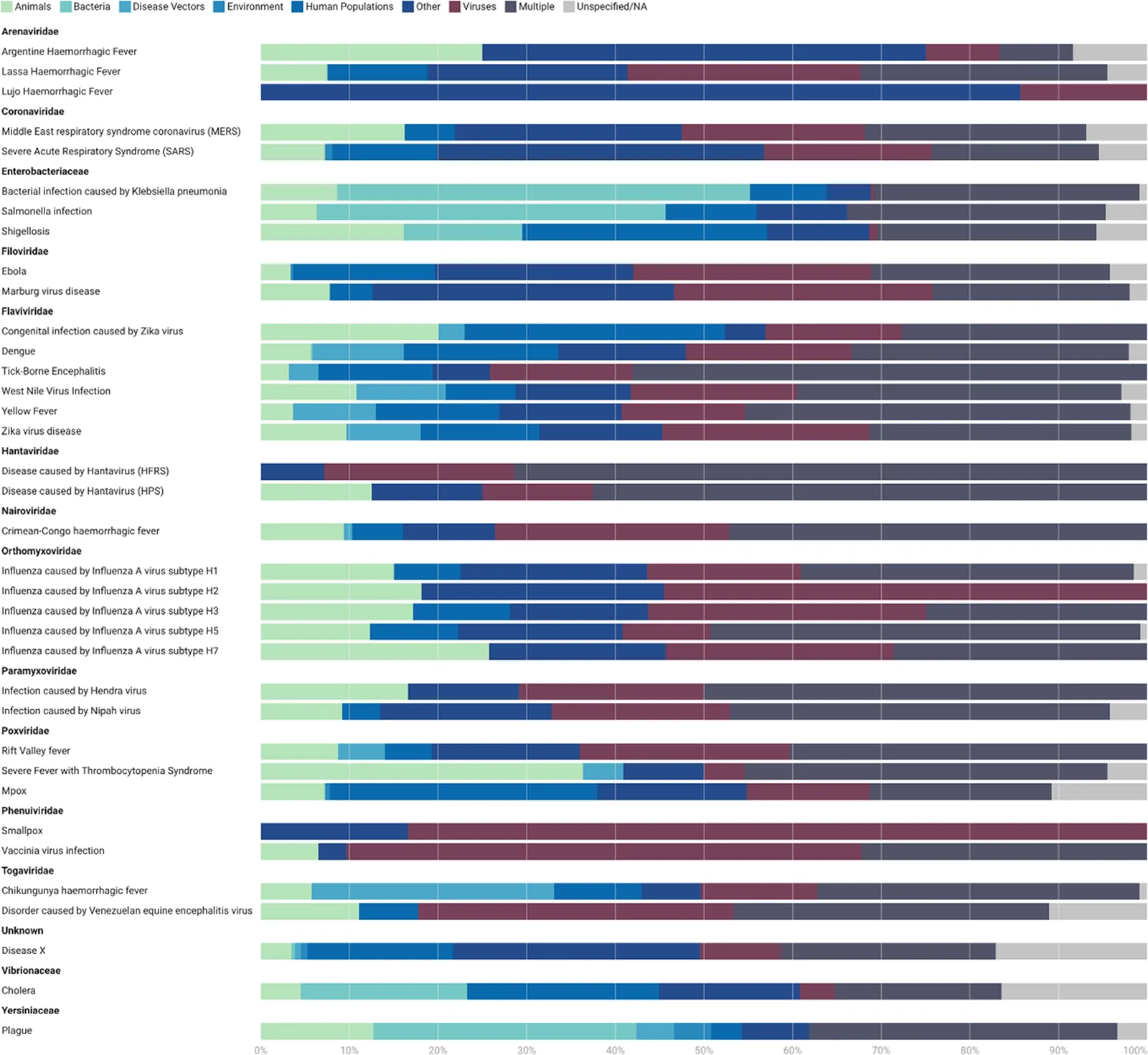
Percentage and total number of awards for research involving human population subjects across Pandemic PACT list of diseases. Distribution of the total number of awards allocated across different study populations by disease. Data cover the period from 1 January 2020 to 6 December 2025.

### Specific human populational groups in high-risk infectious diseases research

When human populations are included in the research, the Pandemic PACT database provides insights into several sociodemographic characteristics of these populations. This information is crucial for improving the coordination and efficient allocation of resources, especially during health emergencies, as specific populations may face heightened risks. Pandemic PACT categorises human populations according to ethnicity, age group, geographical scope (urban or rural), vulnerable group, and occupational group. Detailed descriptions of each characteristic and its corresponding categories can be found in the Pandemic PACT published protocol. ^18^

Figure 14 presents the distribution of awards for research involving human populations across the diseases in the Pandemic PACT database, alongside the proportional breakdown of awards targeting specific population groups. For instance, of the 21 awards related to Congenital infection caused by Zika virus involving human populations, 76.2% focused on children, while 33.3% included women. This is consistent with the nature of Congenital Zika, which primarily involves birth defects and complications arising from Zika virus infection during pregnancy. Similarly, in research on Mpox, 23.2% of the 69 awards that involved human populations targeted vulnerable groups, particularly men who have sex with men, who have been disproportionately affected by recent outbreaks.

**Figure 14.**
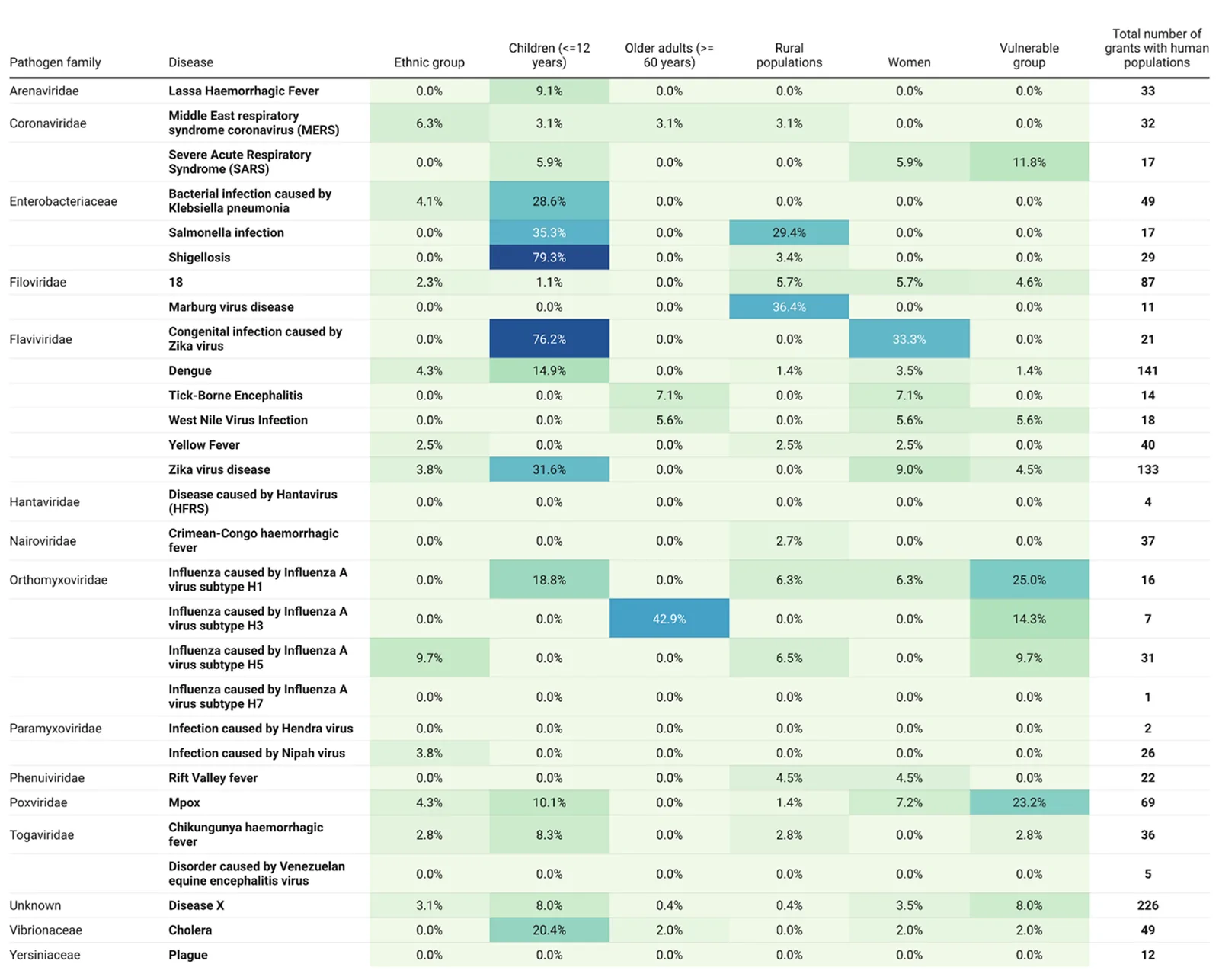
Percentage of awards involving specific populational groups across Pandemic PACT list of diseases. Distribution of the total number of awards allocated across different populational groups by disease. Data cover the period from 1 January 2020 to 6 December 2025.

The only disease where older adults were specifically included as study subjects in more than one award is Influenza caused by Influenza A virus subtype H3. This focus may be explained by the fact that older adults are at a higher risk of severe complications from influenza and related health issues. Additionally, research on Marburg virus disease included 36.4% of awards involving human populations that focused on rural populations, reflecting the tendency for outbreaks of this virus to disproportionately affect such areas. These findings illustrate the existing distribution of research investments across different sociodemographic groups. While this information does not provide insight into whether the investments are sufficient, they offer a mapping of current research priorities, which can inform ongoing and future efforts to address the needs of specific populations during health emergencies.

### Study type in high-risk infectious diseases research

The Pandemic PACT database also provides information on the study type of funded research when specified. Pandemic PACT classifies research as either clinical or non-clinical, with clinical studies broadly defined as those involving primary data collection on humans to diagnose, characterise, describe, or conduct disease surveillance.

Across all diseases in the Pandemic PACT database, the majority of grants support non-clinical research. Some diseases, such as Argentine Haemorrhagic Fever, HFRS, HPS, Influenza caused by Influenza A virus subtypes H2 and H7, Lujo Haemorrhagic Fever, Severe Fever with Thrombocytopenia Syndrome, Smallpox, and Vaccinia virus infection, received no funding for purely clinical research. Shigellosis has the highest proportion of awards dedicated exclusively to clinical research, accounting for 20% (n = 21/105 of its total funded awards. Figure 15 provides a detailed overview of the total number of awards supporting clinical research across diseases in the Pandemic PACT database, alongside the proportional distribution of awards for non-clinical and mixed-type research. The illustration attempts only to describe study type and should not be interpreted as endorsement of value of one type over another, given both non-clinical and clinical research are integral to pandemic preparedness and response.

**Figure 15.**
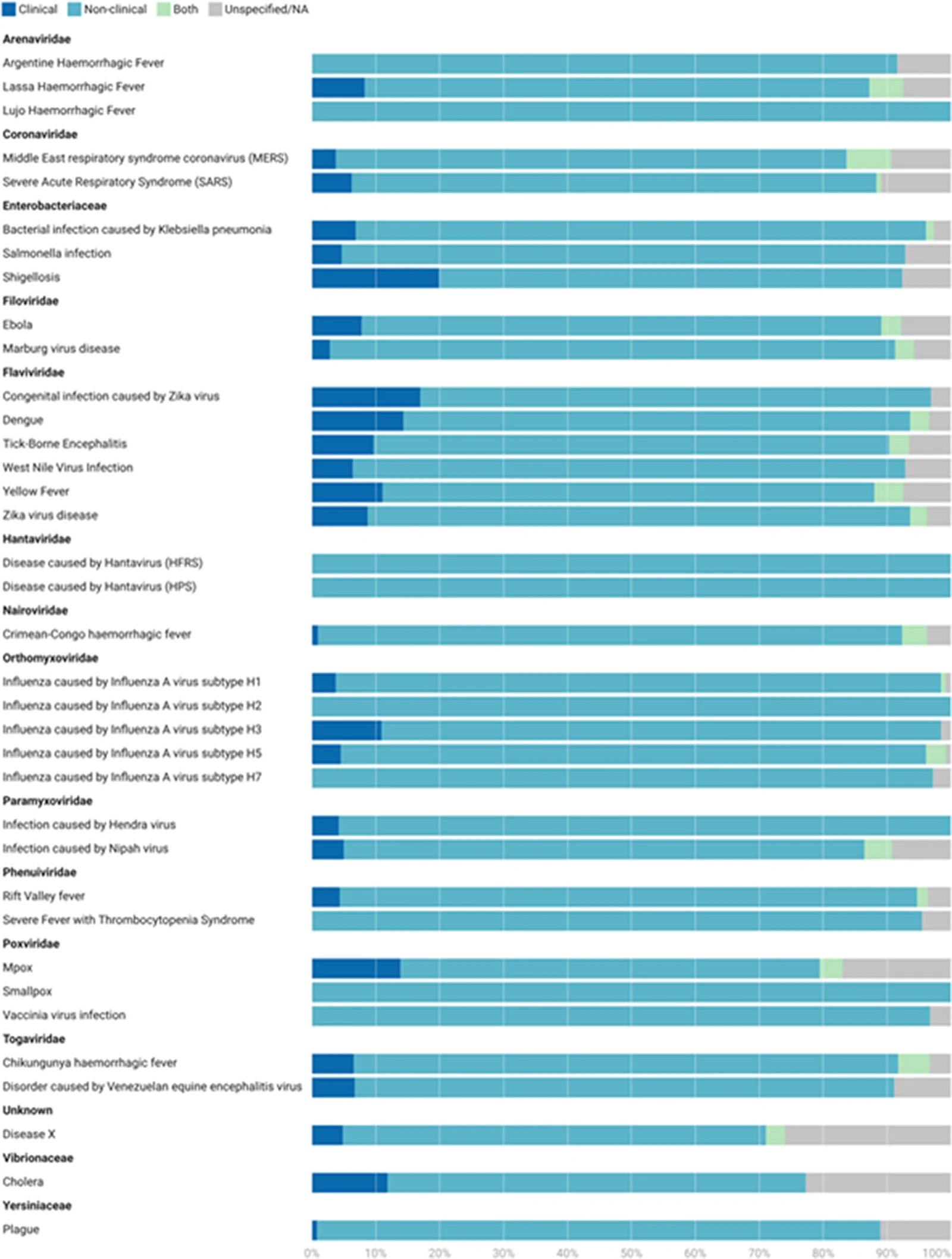
Percentage and total number of awards by study type across Pandemic PACT list of diseases. Distribution of the total number of awards allocated across different study types by disease.

While the number of awards allocated to clinical versus non-clinical research provides useful comparative information, it does not fully capture the financial commitments associated with each study type. As shown in Figure 16, although non-clinical research receives a higher proportion of total awards across diseases, financial commitments do not always follow the same pattern. For instance, despite clinical research accounting for only 14% of Mpox grants, it received $146.9 million in known financial commitments—more than the $130.2 million allocated to non-clinical Mpox research. Notably, a substantial portion of financial commitments for the concept of ‘Disease X’ were allocated to awards that did not specify the study type, amounting to $893.8 million. Figure 16 provides a detailed breakdown of financial commitments across study types and diseases, illustrating investment priorities for clinical and non-clinical research.

**Figure 16.**
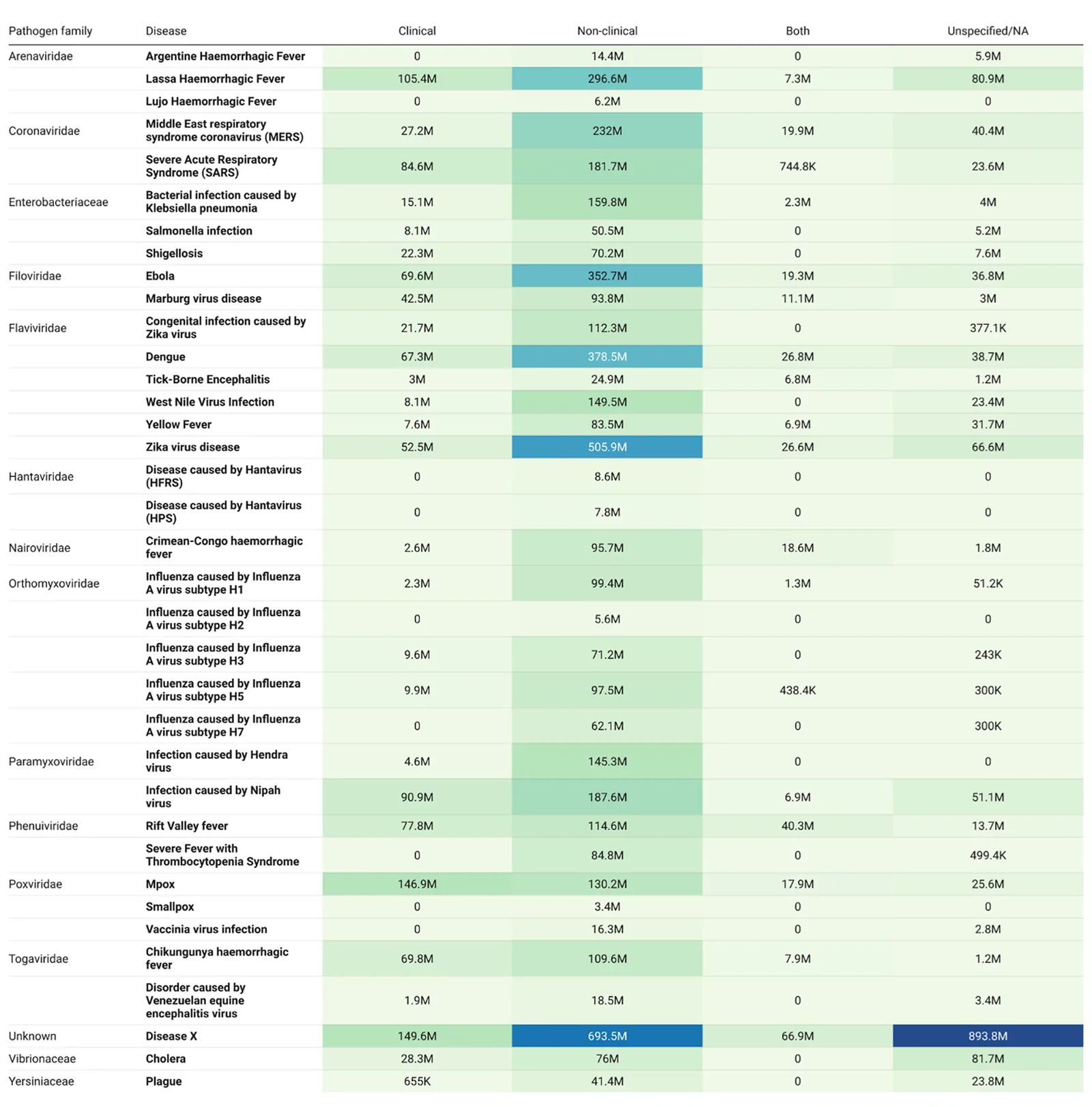
Total known funding distribution across Pandemic PACT list of diseases and study type. Funding amounts are included only when they have been published by the funder. Data cover the period from 1 January 2020 to 6 December 2025. Financial commitment information is available for 76% of the awards in the Pandemic PACT database (n = 21,416/28,176). Although awards may be granted to awards covering multiple diseases, this is uncommon, with only 9% of awards with known financial commitments involving funding for awards covering more than one disease (n = 1,928/21,416). In such cases, there is a possibility of double counting, as the same financial commitment may be recorded under multiple diseases.

## Discussion

The COVID-19 pandemic prompted unprecedented levels of research funding. Importantly, one of its lasting impacts has been a growing recognition of the need to strengthen research preparedness for a broader range of high-risk infectious diseases. ^21^ Pandemic PACT is designed to facilitate that greater preparedness, through providing a broader understanding of the existing research investments and identifying critical investment gaps across high-risk pathogens, research areas, and geographic scope as well as the nature of funders who support this high-risk pathogen research, particularly in LMICs. This paper presents the first analysis update of the Pandemic PACT dataset. Compared to the baseline analysis, this update reflects an expanded pathogen scope and a growing volume of tracked awards, allowing for a more comprehensive assessment of trends over time. By extending the scope of analysis beyond COVID-19 to the full range of high-risk pathogens tracked by Pandemic PACT, this study advances prior work, including the COVID CIRCLE living mapping review, ^3^ which, while comprehensive in its coverage of COVID-19 research funding, did not provide a systematic analysis of the broader infectious disease research funding landscape.

Our analysis shows that funding patterns have generally aligned well with the Pandemic PACT list of priority research areas, suggesting that the framework provides broad coverage of the research landscape. Overall, 95.35% of awards were classified within at least one research area, with only 4.65% (n = 1,310/28,176) not assigned to a research area, either because the available information was insufficient for classification or because the award did not fit any of the predefined categories. Nevertheless, the breadth of funding across these research areas varied substantially by disease, with several diseases receiving funding across only a limited subset of research areas. Notable gaps persisted in diagnostics, later-phase clinical trials, research into ethical issues, health systems research, research on infection prevention and control, and capacity strengthening, consistent with the patterns identified in the baseline analysis. One persistent investment gap in infectious disease and pandemic preparedness research is the continued lack of funding for research to ethical issues. This pattern was already present in the COVID CIRCLE portfolio ^3^ and has carried over into the broader Pandemic PACT dataset across broader diseases. Despite widespread recognition of the importance of ethical considerations, ^12^ ^,^ ^25^ ^,^ ^26^ this area remains to receive limited attention within global research funding priorities.

The Pandemic PACT dataset also reveals that several areas of research have received comparatively less investment across the broader landscape of high-risk pathogens. Though it was pivotal to the COVID-19 pandemic response, financial commitments towards research on infection prevention and control rank among the lowest of the twelve broad research categories in the Pandemic PACT dataset. Similarly, funding for research on policies for public health, disease control, and community resilience has declined in prominence compared to the COVID-19 research portfolio. Funding for diagnostics, which in the Pandemic PACT research categorisation framework is a sub-category within the broader pathogen-related research area, has also declined relative to COVID-19. Compared to the baseline, this update suggests these trends have persisted, with no notable relative increase in funding directed toward these areas. This discrepancy may reflect the exceptional nature of the global response to COVID-19, where the urgent need to contain a rapidly spreading virus catalysed an unprecedented mobilisation of political will and research investments. In contrast, other high-risk infectious diseases often lead to more localised or slower-moving outbreaks that do not trigger the same level of sustained attention or funding urgency. As a result, critical areas of research that are important to effective preparedness and long-term resilience have received comparatively lower levels of investment.

In contrast, one shift compared to the COVID-19 research investments observed in the COVID CIRCLE analyses is the greater financial commitments allocated to research on animal and environmental research and research on disease vectors among awards in the Pandemic PACT database. During the COVID-19 pandemic, this area received relatively lower levels of funding. ^3^ However, in the Pandemic PACT dataset, research investments in this area aligned more frequently across diseases. While the inclusion of vector-borne diseases in the list of high-risk diseases tracked by Pandemic PACT, such as Crimean-Congo haemorrhagic fever, Dengue, Rift Valley fever, Chikungunya and Zika virus diseases, among others, helps account for increased funding in areas like vector biology and vector control strategies, it does not fully explain the relative rise in investment in this broader research area. This trend might also reflect a growing alignment with the One Health approach, ^27^ which emphasises the interconnectedness of human, animal, and environmental health in the emergence and transmission of infectious diseases, suggesting a greater recognition of the complex ecological dynamics that drive pandemics. Indeed, this pattern, already present in the baseline, have been maintained in the current update, suggesting a consolidating trend toward One Health-aligned research priorities.

A relatively greater share of research funding in the Pandemic PACT dataset continued to focus on vaccine and therapeutic development and implementation, as well as on pathogen morphology, transmission dynamics, and diagnostics. However, even within these high-priority areas, notable gaps remain. Pathogen-focused research was largely concentrated on Bacterial infection caused by Klebsiella pneumonia, Dengue, Ebola, Zika virus disease, and the concept of ‘Disease X’. While studies on pathogen morphology, viral shedding, and natural history received comparatively higher levels of investment, areas such as diagnostics, disease modelling, and the environmental stability of pathogens attracted less attention. In therapeutic research, the majority of funding was directed toward pre-clinical studies, which encompass early-stage drug development activities such as laboratory-based experiments and testing in animal or cell models. A notable share also supported clinical trials without a specified phase, including randomised controlled trials. Yet, key gaps persist: no funding was identified for Phase 4 therapeutic trials, while research into adverse events associated with therapeutic use was limited to Mpox-related studies. Vaccine research showed a somewhat broader spread, with most funding directed toward Phase 1 and Phase 2 trials, particularly for Lassa haemorrhagic fever, MERS, Mpox, Infection caused by Nipah virus, Rift Valley fever, and SARS. However, similar to therapeutics, no funding was allocated to Phase 4 vaccine trials, except for financial commitments directed to research on Mpox. While funding was allocated to all pathogen families on the Pandemic PACT list for this broad research area, some diseases lacked funding for any vaccine clinical studies. This was the case for Argentine Haemorrhagic Fever, Bacterial infection caused by Klebsiella pneumonia, Salmonella infection, Shigellosis, Congenital infection caused by Zika virus, Tick-Borne Encephalitis, West Nile Virus Infection, Yellow Fever, HFRS, HPS, Crimean-Congo haemorrhagic fever, Influenza caused by Influenza A virus subtypes H1, H2, and H7, Infection caused by Hendra virus, Severe Fever with Thrombocytopenia Syndrome, and Cholera. Notably, pre-clinical research received commitments across the entire spectrum of high-risk pathogens included in Pandemic PACT. Beyond the clear gaps at the priority level, it is inherently difficult to conclude when a particular priority area has received sufficient research funding or research projects at the grant award stage, as this is only apparent when the outcomes are achieved and it is clear that the research question has been sufficiently answered.

The disaggregation of research awards by population groups in the Pandemic PACT dataset offers useful insights into how sociodemographic considerations are being integrated into research on high-risk diseases and serves as an important tool for informing policy and ensuring that research activities engage the full range of populations affected by high-risk diseases. Our analysis is limited to research awards that contain statements of including populations that have been identified as vulnerable. In some instances, study populations appear to reflect disease-specific risk profiles. For example, research on Congenital Zika included children and women and Mpox-related awards showed some attention to vulnerable groups. However, the overall inclusion of specific population groups remains limited across the dataset. Ethnic groups were included in a small number of awards related to Dengue, the concept of ‘Disease X’, Influenza caused by Influenza A virus subtype H5, Mpox, and Zika virus disease, while representation in research on other diseases was either minimal or entirely absent from the dataset. Similarly, vulnerable groups were not identified in awards related to Lassa Haemorrhagic Fever, Bacterial infection caused by Klebsiella pneumonia, Shigellosis, Salmonella infection, Marburg virus disease, Plague, Congenital infection caused by Zika virus, Tick-Borne Encephalitis, Infection caused by Nipah virus, HFRS, Crimean-Congo haemorrhagic fever, Influenza caused by Influenza A virus subtype H7, MERS, Infection caused by Hendra virus, Rift Valley fever, Yellow Fever, and Disorder caused by Venezuelan equine encephalitis virus. These gaps, compared to the baseline, have persisted across the portfolio, highlighting the need for clearer indication and more systematic inclusion of diverse population groups in research design and funding strategies, to ensure that future preparedness efforts are both equitable and responsive to the needs of those most at risk.

Another clear gap identified in our dataset is the relative lack of research conducted in LMICs, which highlights inherent structural imbalances within the global health research ecosystem. This pattern is consistent with the baseline and shows no substantive change in the current update, with only 8.8% of total number of awards involving at least one research institution based in an LMIC, compared with 11.5% in the baseline. These findings indicate that the imbalances identified previously remain in place. Despite growing recognition of the need for more equitable research investment, particularly in regions that bear a disproportionate burden of infectious disease outbreaks, ^5^ ^,^ ^28^ research continues to be concentrated in HICs (based on the latest version of the PACT database). However, the magnitude of this imbalance should be interpreted in light of limitations in data availability. Financial data completeness is lower for awards involving LMIC institutions than for the dataset overall (49.3% vs. 76%, respectively), which may result in the financial share attributed to LMIC institutions being underestimated. This limitation is compounded by the incomplete capture of intramural research funding in publicly available sources. While intramural funding occurs in both HICs and LMICs, the limitation is likely more pronounced in LMICs, where a greater proportion of domestic research activity may be funded through such mechanisms and therefore remain less visible to analyses relying on publicly available data.

Since the COVID-19 pandemic, the global distribution of funding has shifted slightly, with more funding calls and announcements related to LMICs and some domestic funding captured, such as in Brazil and India. Although some funders based in HICs, especially in the UK, US, and Canada, increasingly have made efforts to direct research investments toward LMICs, along with the growing recognition that context-specific research is needed in LMICs, our first updated analysis shows only a modest expansion of funding investments toward LMICs from ($1.3 to $1.5 billion), while its share of total funding declined from 9.0% to 5.9%. This persistent concentration reflects structural barriers within international funding mechanisms that systematically disadvantage LMIC-based applicants, limited institutional capacity in many LMIC settings, and geopolitical factors that may shape where research funding flows. Moreover, expected declines in official development assistance (ODA) in 2025, ^29^ combined with planned cuts in US spending on medical and scientific research, ^30^ ^,^ ^31^ public health, and disease-prevention programmes, are likely to further affect LMICs and exacerbate existing vulnerabilities. These imbalances limit the production of context-specific evidence that is critical for effective and locally relevant responses.

To ensure that research investments translate into meaningful impact, there is an urgent need for stronger coordination and continuous alignment with local research priorities. The trends observed between the baseline and this update show that, while the volume of tracked research has grown, the underlying distribution of investment has changed relatively little. This is particularly critical given the diverse nature of disease outbreaks, which vary in their modes of transmission, affected populations, and contextual needs. Real-time tracking of research funding is crucial in such fast-moving environments, where timely, informed decisions can shape the trajectory of an effective response. Beyond pandemic contexts, this capacity for timely and transparent monitoring holds significant value for other global research efforts, where coordinated action across borders and disciplines is increasingly essential. It should be noted that this analysis captures financial commitments rather than research outputs or outcomes. Funding allocation does not in itself guarantee successful implementation, knowledge generation, or policy uptake. Linking investment data to measurable downstream outcomes, such as published findings, product development milestones, or strengthened health systems, remains an important direction for future work. Nonetheless, the persistence of fragmented, underpowered, and often duplicative nationally funded studies highlights the need for both funders and researchers to adopt more strategic, collaborative approaches to maximise the efficiency and utility of research during health emergencies. Tools such as the Pandemic PACT tracker play a crucial role in enabling this shift, facilitating transparency, fostering global collaboration, and strengthening the collective capacity to direct research funding where it is most needed and where it can have the greatest impact.

## Conclusion

We have provided a comprehensive review and descriptive analysis of funded research across high-risk pathogen families, as captured in the Pandemic PACT dataset, representing an important step forward in our ability to track, map, and evaluate global research funding for pandemic disease threats. Key research gaps in funding remain in diagnostics, health systems, and ethics research, while most vaccine and therapeutic studies are still at early stages. Vulnerable populations are rarely stated to be included or identifiable in research award information, and context-specific research in LMICs receives limited financial support. From a health policy perspective, these findings point to the need for greater equity considerations in how research investments are designed and allocated, particularly in light of anticipated declines in official development assistance and the increasing need for transparent, real-time tracking of funding flows. We encourage the research community to make use of these findings and the Pandemic PACT tracker tool to inform evidence-based decisions on future research prioritisation, guided by an understanding of existing research efforts. We also urge research funders to continue contributing their data to the tracker, thereby enhancing its utility as a resource for improving pandemic preparedness through greater transparency and early visibility of research funding and activity during emerging outbreaks.

## Data Availability

The continuously updated Pandemic PACT data and the specific dataset used for this study are openly available on Figshare platform at https://doi.org/10.25446/oxford.26937448 and https://doi.org/10.25446/oxford.34069401, respectively. The data can also be explored and exported on the online public dashboard at https://www.pandemicpact.org, which includes high-quality figures and charts, also available for downloading. Figshare: Extended data for: ‘Living mapping review of global research funding for infectious diseases with a pandemic potential – Pandemic PACT Grant Tracker’. https://doi.org/10.25446/oxford.34069401 This project contains the following extended data:
•
Figures 1–16. png • Extended Data 1. Total number of grants, known financial commitments, and percentage of grants with funding amount information available by funding organisation, sorted by total known financial commitments.docx • Extended Data 2. Funding data sources.docx • Extended Data 3. Pandemic PACT research categories and subcategories.docx • Extended Data 4. Global distribution of total number of awards by funder location. Global distribution of total number of awards by research institution location. • Extended Data 5. Total known funding distribution across Pandemic PACT list of diseases and pathogen research sub-categories.docx Figshare: PRISMA checklist for ‘Living mapping review of global research funding for infectious diseases with a pandemic potential – Pandemic PACT Grant Tracker’. https://doi.org/10.25446/oxford.30296383 Data are available under the CC BY 4.0 license.

## Footnotes

^1^
Country income classifications were determined based on the Official Development Assistance (ODA) recipient list, as defined in the OECD’s 2024 and 2025 flows report. A detailed list of countries classified as low- and middle-income can be found at https://www.oecd.org/en/topics/sub-issues/oda-eligibility-and-conditions/dac-list-of-oda-recipients.html
