# Supplementary material for: Living mapping review of global research funding for high-risk pathogens-Pandemic PACT Grant Tracker
Source: Wellcome Open Res. 2026 Nov 2;10:582. Originally published 2025 Oct 17. [Version 3] doi: 10.12688/wellcomeopenres.24958.3 (PMC13760147; doi:10.12688/wellcomeopenres.24958.3)
Supplement: Peer review history

# Reviewer report: Reviewer response for version 3

**DOI:** 10.21956/wellcomeopenres.30875.r177634

**Published:** 2026-11-12

**Authors:** Chizaram Onyeaghala

**Affiliations:** 1 University of Port Harcourt Teaching Hospital, Port Harcourt, Nigeria

The authors have addressed the comments I raised. Well done to the authors for this important article.

Are the rationale for, and objectives of, the Systematic Review clearly stated?

Yes

Is the statistical analysis and its interpretation appropriate?

Partly

If this is a Living Systematic Review, is the ‘living’ method appropriate and is the search schedule clearly defined and justified? (‘Living Systematic Review’ or a variation of this term should be included in the title.)

Partly

Are sufficient details of the methods and analysis provided to allow replication by others?

Partly

Are the conclusions drawn adequately supported by the results presented in the review?

Partly

Reviewer Expertise:

emerging infectious diseases, global health

I confirm that I have read this submission and believe that I have an appropriate level of expertise to confirm that it is of an acceptable scientific standard.

# Reviewer report: Reviewer response for version 2

**DOI:** 10.21956/wellcomeopenres.29857.r160092

**Published:** 2026-08-11

**Authors:** Anna Górska

**Affiliations:** 1 Faculty of Fundamental Problems of Technology, Wrocław University of Science and Technology (Ringgold ID: 49567), Wrocław, Lower Silesian Voivodeship, Poland

Dear Authors,

 thank you very much for the extensive reply to the previous comments!

Are the rationale for, and objectives of, the Systematic Review clearly stated?

Yes

Is the statistical analysis and its interpretation appropriate?

Yes

If this is a Living Systematic Review, is the ‘living’ method appropriate and is the search schedule clearly defined and justified? (‘Living Systematic Review’ or a variation of this term should be included in the title.)

No

Are sufficient details of the methods and analysis provided to allow replication by others?

Partly

Are the conclusions drawn adequately supported by the results presented in the review?

Yes

Reviewer Expertise:

Bioinformatics and Medical informatics specialised in infectious diseases and pandemic preparedness.

I confirm that I have read this submission and believe that I have an appropriate level of expertise to confirm that it is of an acceptable scientific standard.

# Reviewer report: Reviewer response for version 2

**DOI:** 10.21956/wellcomeopenres.29857.r160458

**Published:** 2026-08-10

**Authors:** Chizaram Onyeaghala

**Affiliations:** 1 University of Port Harcourt Teaching Hospital, Port Harcourt, Nigeria

Reviewer comments

 This manuscript presents an ambitious living mapping review of global research funding for pandemic-prone infectious diseases using the Pandemic PACT database. The analysis of 20,362 grants from 326 funding organisations across 58 countries provides potentially valuable evidence for understanding the distribution of research investment across diseases, research areas, geographies and study populations. The manuscript is particularly relevant to pandemic preparedness because it extends previous COVID-19-specific funding analyses to a broader range of epidemic- and pandemic-prone diseases and establishes a baseline for future living updates.

 The principal strengths are the large dataset, broad disease and geographic scope, longitudinal design, transparent description of important limitations, and clear potential policy relevance. The manuscript also identifies several important patterns, including concentration of funding in high-income countries, dominance of vaccine and therapeutic research, relatively limited investment in ethics and health systems research, and limited representation of LMIC-led research and vulnerable populations.

 However, I have several substantive concerns that should be addressed before indexing. My main concern is that the manuscript sometimes appears to interpret the Pandemic PACT database as representing the global funding landscape as a whole, whereas the authors themselves demonstrate that the database is affected by incomplete financial information, differential representation of funders, limited capture of intramural funding, language restrictions, and underrepresentation of some LMIC funders. Consequently, the findings should consistently be framed as describing the funding captured in the Pandemic PACT database rather than as an unbiased estimate of all global research funding.

 Major comments

1. **Clarify the methodological identity of the study.** The manuscript alternates between "systematic review," "living systematic review," and "living mapping review." Because the unit of analysis is research grants rather than individual research studies and no study-level risk-of-bias assessment is undertaken, the authors should clearly define the methodology and explain the reporting framework used.
2. **Clarify the comprehensiveness of the database.** The manuscript should distinguish between the global funding landscape and the subset of funding identifiable through Pandemic PACT. This distinction is particularly important given the acknowledged overrepresentation of GloPID-R/UKCDR-linked funders, incomplete funder participation, limited publicly available information, and potential undercapture of LMIC domestic funding.
3. **Strengthen the treatment of LMIC funding.** Financial information is available for 68.7% of grants overall but only 46.5% of LMIC-led grants in the relevant analysis. Consequently, the reported $1.3 billion and 9% share of known funding for LMIC-led grants should not be interpreted as an unbiased estimate of the proportion of global investment received by LMIC institutions. Please consider sensitivity analyses or substantially strengthen the qualification of these comparisons.
4. **Clarify funder geography versus research-recipient geography.** Funding-source location and research-institution location represent distinct dimensions. These should be explicitly defined and consistently differentiated throughout the manuscript and figures.
5. **Address intermediary funding and potential double counting.** The treatment of organisations such as CEPI, which itself receives resources from other funders represented in the database, needs greater methodological clarification. Readers should be told whether the same financial contribution can appear at multiple points in the funding chain and how this affects interpretation of funder rankings.
6. **Clarify the meaning of minimum and potential funding.** The upper-bound/potential funding estimates can substantially overstate the amount actually attributable to an individual disease or research area when grants cover multiple diseases or categories. Please provide a clearer conceptual explanation and consider terminology such as "upper-bound allocation estimate." It should also be made explicit that these estimates cannot be summed across overlapping categories.
7. **Distinguish low funding from underfunding or research gaps.** The database demonstrates differences in observed funding, but it cannot establish whether a particular disease or research area is adequately or inadequately funded without an external benchmark of need. Terms such as "underfunded," "critical research gap," and "research gap" should therefore be used cautiously and preferably replaced by "comparatively limited funding" or "funding gap identified in the dataset" where appropriate.
8. **Provide greater detail on grant classification.** Please report the number and background of coders, training procedures, whether all grants were independently double-coded, how disagreements were handled, and whether inter-rater reliability was assessed. This is particularly important because many subsequent findings depend on human classification of diseases, research areas, study populations, and study types.
9. **Clarify the search and data acquisition strategy.** The manuscript should provide sufficient information to reproduce the baseline search, including the databases and websites searched, exact search terms or a direct reference to the relevant supplementary material, search dates, automated versus manual retrieval, and procedures for non-English records.
10. **Provide a clearer breakdown of exclusions in the PRISMA flow diagram.** The numerical totals appear internally consistent, but the reasons for exclusion should be reported separately for automated pre-screening and manual review. This would improve transparency and reproducibility.
11. **Clarify the disease/pathogen scope.** The manuscript should provide an explicit list of all diseases/categories included in the baseline analysis and the rationale for each. In particular, the treatment of Congenital Zika separately from Zika, SARS separately from MERS-CoV, Hendra virus infection, and pandemic-prone influenza requires clarification. The relationship between the baseline classification and the 2024 WHO pathogen-family framework should also be explained.
12. **Address longitudinal comparability following the planned WHO framework expansion.** Because the living review will incorporate the 2024 WHO pathogen-family framework, the authors should explain how future updates will remain comparable with the current baseline. Maintaining both historical and updated classifications may be important.
13. **Qualify annual funding analyses.** Because the analysis uses grant start dates rather than actual expenditure by year, the results should be described as financial commitments associated with grants initiated in each year rather than annual expenditure or annual funding. A large multi-year grant initiated in one year should not be interpreted as funding spent entirely in that year.
14. **Strengthen the evidence supporting comparative claims.** Statements that diagnostics "declined" relative to COVID-19, that funding is "aligned well" with priority areas, or that particular trends reflect One Health or outbreak-related changes should either be supported by quantitative comparative analyses or presented more cautiously as possible interpretations.
15. **Reconsider conclusions regarding vulnerable populations.** The finding that vulnerable populations are rarely included may partly reflect incomplete information in grant titles and abstracts and the limitations of human annotation. The manuscript should distinguish between vulnerable populations not being included and vulnerable populations not being identifiable from the available grant information.
16. **Clarify study-type classification.** The distinction between clinical and non-clinical research is useful, but non-clinical research includes important human-relevant activities such as modelling, epidemiology, health systems and policy research. The manuscript should avoid implying that non-clinical research is less relevant to preparedness.
17. **Audit all denominators and numerical inconsistencies.** I noted apparent differences in the number of LMIC-related grants and in denominators used in some figure legends, including the 2,336 versus 2,366 LMIC-related grants and the different denominators accompanying financial completeness in Figure 4. These may have legitimate explanations, but they should be reconciled explicitly.

Minor comments

- Standardise terminology for "pandemic-prone infectious diseases," "infectious diseases with pandemic potential," and "epidemic-prone infectious diseases."
- Define "Disease X" consistently as a preparedness concept rather than a specific disease.
- Consider shortening the very long figure legends and moving methodological qualifications to the Methods or supplementary material where possible.
- Consistently distinguish grant counts from financial commitments, as these measure different dimensions of research investment.
- Review causal or explanatory statements in the Discussion and distinguish observed findings from hypotheses about why funding patterns occurred.
- Ensure that all claims concerning ODA, changes in US research funding, structural barriers, One Health, and global research inequities are appropriately referenced.
- Consider adding a supplementary table showing the frequency of "unspecified" and "N/A" classifications across major variables.

Overall recommendation

 The manuscript has considerable potential and addresses an important policy-relevant gap. However, substantial methodological clarification and more cautious interpretation are required, particularly regarding database completeness, LMIC funding estimates, multi-disease grants, funder relationships, grant classification, and the distinction between observed funding disparities and actual unmet research needs.

**Recommendation: Major revision.**

Are the rationale for, and objectives of, the Systematic Review clearly stated?

Yes

Is the statistical analysis and its interpretation appropriate?

Partly

If this is a Living Systematic Review, is the ‘living’ method appropriate and is the search schedule clearly defined and justified? (‘Living Systematic Review’ or a variation of this term should be included in the title.)

Partly

Are sufficient details of the methods and analysis provided to allow replication by others?

Partly

Are the conclusions drawn adequately supported by the results presented in the review?

Partly

Reviewer Expertise:

emerging infectious diseases, global health

I confirm that I have read this submission and believe that I have an appropriate level of expertise to state that I do not consider it to be of an acceptable scientific standard, for reasons outlined above.

# Response

**Published:** 2026-10-07

**Authors:** Alice Norton

**Affiliations:** University of Oxford, UK

Response to Reviewer Comments  

 We thank the review for the helpful and constructive comments. We have addressed these below, using the same numbering system as in the review. We have now updated the manuscript both in response to this review but also to present an updated analysis of the data set six months on from the baseline (given this is a living review).  

**Response** **in relation to major comment 1: **We have amended statements throughout the manuscript to consistently indicate that we are conducting a “living mapping review”. As the unit of analysis are research awards, it is inappropriate to perform a risk-of bias assessment as there are no existing tools, guides, or guidelines for reporting risk-of-bias assessments. We used the PRISMA guideline as it is the closes applicable reporting guideline. We have included additional information on our methodology where appropriate.  

**Response** **in relation to major comment 2: **We have amended statements throughout the manuscript, where applicable, to ensure a clear distinction between the global funding landscape and the funding identified through the Pandemic PACT grant tracker.  

**Response** **in relation to major comment 3: **We have added a caveat directly alongside the reported $1.3 billion and 9% share ($1.5 billion and 5.9% in this first LMR update) in the Results (Research institutions section), highlighting the substantially lower completeness of financial data for LMIC-led awards (46.5%; 49.3% in the LMR update) compared with the dataset overall (68.7%; 76% in the first LMR update). We now also report the corresponding award-count-based share (11.5%; 8.8% in this first LMR update), which is not subject to the same differential availability of financial data. Although the award-count-based estimate does not address the underlying bias arising from incomplete financial data, it provides further support for the finding that, within the Pandemic PACT dataset, LMIC institutions receive a relatively small share of global research funding allocations. In addition, we have added a sentence to the Discussion linking this financial-data gap to our existing discussion of intramural funding and noting that both factors are likely to result in our reported estimates of LMIC-led investment understating the true scale of LMIC research funding commitments. **Response** **in relation to major comment 4: **Funder geography and research-recipient geography are differentiated through subheadings. We have also amended Figure 6 by adding x and y-axis labels to better differentiate between these groups.  

**Response** **in relation to major comment 5:** We have further addressed limitations in relation to lack of transparency in source data on intermediary funding in the results section (where relevant) and strengthened it in the limitations (where we had already included wording to this effect).  

**Response in relation to major comment 6: **We have replaced the term “potential” with “upper-bound allocation estimate” throughout the manuscript to more clearly reflect the meaning of this measure. We have also expanded the Limitations section to clarify the conceptual rationale for this approach, explicitly noting that upper-bound estimates cannot be summed across overlapping categories (e.g., diseases, research areas, or locations). We further clarify that the minimum and upper-bound estimates should be interpreted as a plausible range for each individual category, rather than as components of a decomposable total. Together, these estimates provide a representation of the plausible funding distribution for each category while acknowledging the limitations of the available data on how funding is allocated within multi-category awards.  

**Response in relation to major comment 7: **We have amended the terms relating to funding level accordingly in various sections of the manuscript.  

**Response in relation to major comment 8: **We have added further detail on grant classification in the ‘Data Processes;’ section of the manuscript including information on grant coding, handling disagreements, and inter-rater reliability, this was already in the protocol publication. We will include further details on training procedures in the protocol manuscript. **Response in relation to major comment 9: **We reference detailed information about our methods in our protocol to allow full reproducibility. We chose to highlight key updates for this manuscript.  

**Response in relation to major comment 10: **We have updated the PRISMA figure and amended statements related to automated pre-screening and manual review.  

**Response in relation to major comment 11: **The first update represents a new alignment to the 2024 WHO pathogen-family framework and greater definition of disease/ pathogen scope alongside this.  

**Response in relation to major comment 12:** We thank the reviewer for raising this. We clarify that the first living mapping review update following this baseline has already transitioned to the WHO's 2024 pathogen-family framework, moving the analysis to the pathogen level with expanded disease/pathogen coverage. We have added a paragraph to the Methods explaining this transition. Importantly, broad funding trends (overall financial commitment, geographic scope, and research-area distribution) are expected to remain comparable across the transition, while disease-specific figures in this baseline may not map one-to-one onto pathogen-family-level figures in later updates, given that some pathogen families aggregate multiple diseases tracked separately here.  

**Response in relation to major comment 13: **We have revised statements that include ‘annual’ in relation to funding and amended them to ‘grants initiated yearly’.  

**Response in relation to major comment 14: **We have revised statements on comparative claims to changes between the baseline analysis and first update  

**Response in relation to major comment 15: **We have amended statements on the identification of grants that include ‘vulnerable populations’ to reflect that these are limited to research awards clearly stating inclusion of specific populations to be vulnerable.  

**Response in relation to major comment 16: **We have updated the wording on the analysis of study type to include greater description of non-clinical research.  

**Response in relation to major comment 17:** We have conducted a full audit of all reported denominators, numerators, and percentages throughout the manuscript and figures. **Responses in relation to minor comments **

- We have standardised our terminology to “high-risk pathogens”

- ** **We have included a definition of disease X in the methodology.

- Figures have all been updated due to the updated data. We prefer to keep some of the details in the legends that affect the interpretation of the figures.

- This version is consistent in distinguishing grant counts from financial commitments.

- ** **We have reviewed and improved causal and explanatory statements in the discussion and distinguished observed findings from hypotheses.

- We have ensured that all claims in the discussion section are appropriately referenced.

- We will explore the inclusion of a supplementary table on the frequency of “unspecified” and “N/A” classifications in the next update.

# Reviewer report: Reviewer response for version 1

**DOI:** 10.21956/wellcomeopenres.27493.r138906

**Published:** 2025-12-29

**Authors:** Anna Górska

**Affiliations:** 1 Faculty of Fundamental Problems of Technology, Wrocław University of Science and Technology (Ringgold ID: 49567), Wrocław, Lower Silesian Voivodeship, Poland

Living mapping review of global research funding for infectious diseases with a pandemic potential – Pandemic PACT Grant Tracker

 I would like to thank the authors for the opportunity to review this manuscript. It presents an impressive body of work that has resulted in a substantial dataset of great importance, significantly enhancing the transparency of national and international research-funding processes.  Please find below my comments and questions:

1. The authors rightly cite the evolving landscape of infectious diseases and funding efforts as the motivation for establishing this review as an ongoing, living effort. To strengthen this claim, they might consider adding a paragraph to the results section that provides metrics demonstrating the rate and scale of changes in the funding landscape over time. Ideally, such a metric could be compared with global statistics on the burden of major infectious diseases with pandemic potential, helping to clarify whether the funding is responding to actual needs.
2. Living mehtodology — The authors refer to other publications and protocols; however, I would still like to understand how the living analysis is actually implemented. Is the data monitored continuously or incorporated in batches? How frequently is it updated? These basic characteristics should be clarified."
3. As shown in Figure 1 of the PRISMA diagram, 222,854 records were removed by deduplication and ‘automation tools.’ However, it is unclear what specific automation tools the authors are referring to. I could not find a description of these tools in the cited protocol (PMID: 39429630). Please clarify, as the lack of transparency represents a potential source of bias
4. Are post-infectious sequelae included within the disease categories? I would appreciate a brief discussion of this. Long COVID has shed new light on PAIS overall, yet these conditions do not appear to be represented—unless I am overlooking them—in either the disease/pathogen categories or the research areas.
5. How do the authors approach the standardisation of the data collected in this effort, specifically, which features are selected and how they are defined? I realise that mapping this particular domain may not be directly representable in existing ontologies, but making the project’s data model accessible could help other stakeholders align with it.
6. The authors acknowledge limitations stemming from the level of detail reported by the funders. Would it be possible to provide a distribution of the transparency metric? Such an analysis could support efforts to encourage funders to publish more complete information, or perhaps even prompt direct engagement with the PANDEMIC pact.
7. Figures:
  Figures 3, 5, 6, 7, 10, 11, and 12 appear to have poor resolution (including in the downloaded PPTX). I am not sure whether this issue comes from the original file or is a limitation of the reviewing system.
  I suggest adding the time period to the figures in the results section to make it clear that they present data from the 2020–2025 period.
  I recommend using the same colour scale range for Figures 2 and 4 so they can be compared side by side. Although the figures appear to use comparable hues, the value ranges differ slightly, making interpretation more difficult.
  In analysis of the figures 2 and 4 authors write: “The distribution of funded research institutions closely reflects the patterns observed in funding sources.” – the choropleth maps (regadless of the colorscale) are difficult to compare; however the scatter chart or a bubble chart could show the correlation as well as point ot the outliers – just a suggestion.
  In Figure 6, the labels of the right axis interrupt the flow of the data; the authors could consider moving them to the right of this axis, and a larger font could add to readability.
  I do not fully understand why figures 8/9 have this very minimalistic form – would it be possible to plot box or violin plots to show more data about the distribution?Living mapping review of global research funding for infectious diseases with a pandemic potential – Pandemic PACT Grant Tracker
    I would like to thank the authors for the opportunity to review this manuscript. It presents an impressive body of work that has resulted in a substantial dataset of great importance, significantly enhancing the transparency of national and international research-funding processes.  Please find below my comments and questions:
  The authors rightly cite the evolving landscape of infectious diseases and funding efforts as the motivation for establishing this review as an ongoing, living effort. To strengthen this claim, they might consider adding a paragraph to the results section that provides metrics demonstrating the rate and scale of changes in the funding landscape over time. Ideally, such a metric could be compared with global statistics on the burden of major infectious diseases with pandemic potential, helping to clarify whether the funding is responding to actual needs.
  Living methodology — The authors refer to other publications and protocols; however, I would still like to understand how the living analysis is actually implemented. Is the data monitored continuously or incorporated in batches? How frequently is it updated? These basic characteristics should be clarified."
  As shown in Figure 1 of the PRISMA diagram, 222,854 records were removed by deduplication and ‘automation tools.’ However, it is unclear what specific automation tools the authors are referring to. I was unable to find a description of these tools in the cited protocol (PMID: 39429630). Please clarify, as the lack of transparency represents a potential source of bias
  4. Are post-infectious sequelae included within the disease categories? I would appreciate a brief discussion of this. Long COVID has shed new light on PAIS overall, yet these conditions do not appear to be represented—unless I am overlooking them—in either the disease/pathogen categories or the research areas.
**How do the authors approach the standardisation of the data collected in this effort** **,** **specifically, which features are selected and how they are defined? I realise that mapping this particular domain may not be directly representable in existing ontologies, but making the project’s data model accessible could help other stakeholders align with it.**
  The authors acknowledge limitations stemming from the level of detail reported by the funders. Would it be possible to provide a distribution of the transparency metric? Such an analysis could support efforts to encourage funders to publish more complete information, or perhaps even prompt direct engagement with the PANDEMIC pact.
  Figures:
    1. Figures 3, 5, 6, 7, 10, 11, and 12 appear to have poor resolution (including in the downloaded PPTX). I am not sure whether this issue comes from the original file or is a limitation of the reviewing system.
    2. I suggest adding the time period to the figures in the results section to make it clear that they present data from the 2020–2025 period.
    3. I recommend using the same colour scale range for Figures 2 and 4 so they can be compared side by side. Although the figures appear to use comparable hues, the value ranges differ slightly, making interpretation more difficult.
    4. In analysis of the figures 2 and 4 authors write: “The distribution of funded research institutions closely reflects the patterns observed in funding sources.” – the choropleth maps (regadless of the colorscale) are difficult to compare; however the scatter chart or a bubble chart could show the correlation as well as point ot the outliers – just a suggestion.
    5. In Figure 6, the labels of the right axis interrupt the flow of the data; the authors could consider moving them to the right of this axis, and a larger font could add to readability.
    6. I do not fully understand why figures 8/9 have this very minimalistic form – would it be possible to plot box or violin plots to show more data about the distribution?
    7. In the Figure 13, it is difficult to distinguish between other and viruses om the colorscale
  In the Figure 13, it is difficult to distinguish between other and viruses om the colorscale

Are the rationale for, and objectives of, the Systematic Review clearly stated?

Yes

Is the statistical analysis and its interpretation appropriate?

Yes

If this is a Living Systematic Review, is the ‘living’ method appropriate and is the search schedule clearly defined and justified? (‘Living Systematic Review’ or a variation of this term should be included in the title.)

No

Are sufficient details of the methods and analysis provided to allow replication by others?

Partly

Are the conclusions drawn adequately supported by the results presented in the review?

Yes

Reviewer Expertise:

Bioinformatics and Medical informatics specialised in infectious diseases and pandemic preparedness.

I confirm that I have read this submission and believe that I have an appropriate level of expertise to confirm that it is of an acceptable scientific standard, however I have significant reservations, as outlined above.

# Response

**Published:** 2026-06-25

**Authors:** Alice Norton

**Affiliations:** University of Oxford, UK

Regarding the changes in funding landscape over time, we would like to draw attention to the subsection on Annual Trends in Global Funding (2020–2024). This section presents temporal trends in financial commitments across diseases, illustrated in Figure 10, and discusses how funding volumes and priorities have shifted over time. In response to the reviewer’s suggestion, we have further strengthened this section by incorporating a discussion that contextualises these funding trends against the global burden and outbreak dynamics of the relevant diseases. We thank the reviewer for this question regarding the data protocol and are happy to clarify. Newly funded research grants are added to the Pandemic PACT database in batches as they become available from funders, and the living mapping review is updated at six-month intervals under normal circumstances. In the event of a major outbreak, analyses for the specific disease are prioritised within the established review cycle, and during a pandemic the full living mapping review transitions to three-monthly updates. We have further elaborated the Rationale for Living Method subsection to consolidate these key information, including the batch data incorporation, the standard six-month update cycle, and the accelerated pandemic schedule. Further details are available in our publicly available protocol (Seminog et al., Wellcome Open Research, 2024; https://wellcomeopenresearch.org/articles/9-156). hank you for highlighting this point regarding the PRISMA diagram. The “automation tools” refer to internally developed Python-based pipelines used for deduplication, pre-screening, and data standardisation. Deduplication was performed using combinations of key fields, including grant identifiers, titles, abstracts, and funder information, with text-based similarity matching applied where unique identifiers were unavailable. Automated pre-screening was used to exclude clearly out-of-scope records based on predefined eligibility criteria. All retained records subsequently underwent manual review and validation within our primary REDCap database by the coding team. We have revised the manuscript (Results, Grant selection) to clarify these processes. Post-infectious sequelae, including Long COVID, are indeed captured within the framework. Broad research category ‘Clinical Characterisation and Management’ includes a dedicated subcategory ‘Post-Acute and Long-Term Health Consequences’, which covers research assessing physical health sequelae of infections, including the incidence and prevalence of post-acute conditions and follow-up for long-term health risks. The full categorisation framework, including all broad categories and subcategories with their definitions, is available in Extended Data 3. We hope this clarifies that these conditions are represented within the analytical framework. Regarding standardisation of data, most variables are extracted directly from information available across grants, including grant amount, research institution location, start and end dates, etc. Each variable is defined in an internal codebook, and where established ontologies exist, we align with them. For example, disease, pathogen, and pathogen family classifications follow SNOMED CT. The research categorisation framework, which required a bespoke approach given the absence of a directly applicable ontology for this domain, is described in full in Extended Data 3. The full data model, variable definitions, and ontology alignments are documented in our publicly available protocol (Seminog et al., Wellcome Open Research, 2024; https://wellcomeopenresearch.org/articles/9-156), which we would encourage stakeholders wishing to align with or build upon this work to consult. We agree with the reviewer that making the data model accessible to other stakeholders is important, and we believe the combination of the published protocol, the codebook, and the openly available dataset provides a solid foundation for this. Regarding funding data limitatoins, following our protocol, all grant records included in the analysis met a minimum level of essential information: grant award, start date, or publication date; funder name; and a grant ID, other identifier, or grant title. We would like to draw attention to Table 1, which presents information on data completeness for each key Pandemic PACT variable, showing the percentage of grants with complete information for each field. This table allows readers to assess the level of reporting transparency across variables and to identify where information gaps are most pronounced. Additionally, Extended Data 1 provides a breakdown of the percentage of grants with funding amount information available by funding organisation, offering further granularity on funder-level reporting practices. We thank the reviewer for raising this comment on figure resolution. The figures were prepared and submitted in accordance with Wellcome Open Research’s format and resolution guidelines. We note that the reviewing system may compress image files, and that the online version of the manuscript is likely to display the figures at higher quality. We have added the time period to the legends of all relevant figures in the Results section to make the temporal scope immediately clear to readers, thank you for this suggestion.  We have standardised the colour scale range across Figures 2 and 4 to facilitate direct comparison, thank you for this suggestion.  We have standardised the colour scale range across Figures 2 and 4 to facilitate direct comparison. For Figure 6, we have repositioned the right axis labels to sit to the right of the axis and increased the font size. We thank the reviewer for this suggestion regarding figures 8/9. However, we would like to clarify that the dumbbell Figures 8 and 9 are not intended to show the distribution or density of individual grant values, but rather to communicate the minimum and maximum potential funding ranges at the disease level, representing, respectively, the minimum known amount allocated exclusively to a given disease and the maximum potential amount if all multi-disease grants were attributed to it. A box or violin plot would not be appropriate for this purpose, as these representations require distributional data across multiple observations rather than two summary estimates per disease.

 We have revised the colour scale for Figure 13 to ensure that ‘other’ and ‘viruses’ are clearly distinguishable, using more contrasting hues to facilitate interpretation.

 We thank the reviewer for their constructive comments.

# Reviewer report: Reviewer response for version 1

**DOI:** 10.21956/wellcomeopenres.27493.r138904

**Published:** 2025-11-18

**Authors:** Mario Coccia

**Affiliations:** 1 National Research Council of Italy, Turin, Italy

Summary.

 The COVID-19 pandemic triggered an unprecedented surge in global research activity, underscoring the need for better coordination and resource allocation to address emerging infectious disease threats. In response, the Pandemic PACT Programme developed a live, publicly accessible database that tracks funded research on pandemic-prone diseases and broader preparedness efforts. As of March 1, 2025, the database includes 20,362 grants from 326 funding organizations across 58 countries, representing at least $14.4 billion in commitments. This study conducted a mapping review of the Pandemic PACT dataset to analyze global research funding trends from 2020 to 2024. The review examined investments across priority diseases and research areas, identifying key patterns and gaps. Findings reveal that while funding generally aligns with priority research areas, significant gaps persist—particularly in diagnostics, health systems research, and ethics. Vaccine and therapeutic research dominate the portfolio, but most projects remain focused on pre-clinical and early-phase trials, with limited support for later-stage studies. Notably, there is growing attention to vector and environmental research compared to earlier COVID-19-focused analyses, reflecting an increasing emphasis on One Health approaches. Despite these advances, funding remains heavily concentrated in high-income countries, with limited financial support for context-specific research led by institutions in low- and middle-income countries (LMICs). Additionally, the inclusion of vulnerable populations in funded research is minimal, raising concerns about equity and responsiveness to those most at risk. Overall, the study highlights critical gaps and imbalances in global research investments for pandemic preparedness. These findings underscore the need for improved coordination, equitable funding distribution, and stronger integration of ethical and health systems research to ensure effective and inclusive responses to future health emergencies.

  ===========================================================================================

 The topics of this paper are interesting, to face next pandemics, though well known, considering previous works. The structure and content must be revised, and results have to be better explained by authors before to be reconsidered.

 The topics is complex, and lessons from recent COVID-19 pandemic show that countries in high ranking of preparedness have had serious problems, such as UK. Moreover, proposed implications are well known, but not easy to implement in poor countries to cope with current and next infectious diseases.

  The title has to be shorter.

 Abstract has to clarify the goal, methods and especially specific health and social policy implications, now they are vague and general.

 Introduction has to better clarify the research questions of this study, indicating the gap presents in literature that this study endeavors to cover, and provide more theoretical background. After that authors can focus on the topics of this study to provide a correct analysis for fruitful discussion. (Please refer to the suggested readings).

  The methods of this study are not clear. The section of Materials and methods must be re-structured. I suggest to clarify soon  Sample and data. Many  subsections can be merged. Limitations are better to put in conclusion and not in section 2.

 A table with inclusion and exclusion criteria of records can support Figure 1 to be clear for readers. Authors have to avoid subheadings that create fragmentation and confusion.

  Section 3. results.

 The completeness and representativeness of the Pandemic PACT dataset remain constrained. The database relies on publicly available information and voluntary reporting by funding organizations, which may lead to gaps in coverage. Smaller funders, private sector contributions, and non-traditional sources of support are likely underrepresented, creating a bias toward large, well-documented grants from high-income countries. This limitation affects the ability to capture the full spectrum of global research activity. Moreover, the analysis reflects a geographic and institutional imbalance. Funding remains heavily concentrated in high-income countries, while low- and middle-income countries (LMICs)—often the most affected by infectious disease outbreaks—receive limited financial support and rarely lead funded projects. This disparity restricts the assessment of context-specific research needs and capacity-building efforts, which are critical for effective pandemic preparedness and equitable global health strategies. The categorization framework used to classify research areas introduces constraints. While grouping projects into broad categories such as diagnostics, therapeutics, and health systems research provides a useful overview, it oversimplifies complex, interdisciplinary projects. Some grants span multiple domains, and assigning them to a single category risks misrepresenting their scope. Furthermore, the lack of granularity within categories—such as distinguishing between early-phase and late-phase clinical trials—limits nuanced interpretation of investment priorities. The temporal scope of the review (2020–2024) coincides with the exceptional context of the COVID-19 pandemic. The unprecedented mobilization of resources during this period may distort long-term trends, as funding patterns driven by COVID-19 urgency do not necessarily reflect typical investment dynamics for other pandemic-prone diseases. This makes extrapolation to future preparedness strategies challenging. In addition, the study focuses on financial commitments rather than research outputs or impacts. Funding allocation does not guarantee successful implementation, knowledge generation, or policy uptake. Without linking investments to measurable outcomes—such as published findings, product development, or strengthened health systems—the effectiveness of funding strategies cannot be fully evaluated. Additionally, ethical and social dimensions of research remain underexplored. Although gaps in ethics-related research and inclusion of vulnerable populations are noted, the study does not analyze underlying causes or propose actionable solutions. These omissions limit the ability to address systemic disparities in research priorities. The dynamic nature of the Pandemic PACT database means that the analysis represents a snapshot in time. Ongoing updates could alter trends and conclusions, particularly as new diseases emerge or funding priorities shift. Continuous monitoring and iterative analysis are therefore essential to maintain relevance and accuracy.

  Figure 2-3-4, of course, show high financial commitments in rich countries as all scientific research. Data in figure should be normalized  with some input to provide realistic results, such as in figure 6, of course US has a high share compared to Canada….Figure 5 shows a total number of grants and total commitments , very high in Brazil, India, that are larger and bigger countries compared to Ethiopia , Lebanon….data presented in this manner are misleading. So revise all figure normalizing them with a standard input indicators.

 The paper has a lot of figures (16!!), no ways, that are difficult to digest, many of them can be put in appendix and inserting in the text the most important ones to improve the readability…

  To reiterate, avoiding sub-headings that create fragmentation of the paper.

  Discussion.

 Although the disaggregation of research grants by population groups in the Pandemic PACT dataset provides valuable insights, several limitations constrain the interpretation of these findings. First, the inclusion of specific sociodemographic groups across funded research remains minimal and inconsistent. While some grants reflect disease-specific risk profiles—such as women and children in Congenital Zika studies or vulnerable groups in Mpox-related research—representation of ethnic minorities and other at-risk populations is largely absent for most diseases. This lack of systematic inclusion limits the ability to assess whether research investments adequately address equity and the needs of those most affected by epidemic-prone diseases. The analysis of LMIC-related research funding is hindered by structural and data limitations. Although the dataset captures some domestic funding in countries like Brazil and India, intramural investments and smaller-scale initiatives often remain undocumented in publicly available sources. Consequently, the true extent of LMIC-led research is likely underestimated. Furthermore, despite increased recognition of the importance of context-specific research, funding continues to be concentrated in high-income countries, with only marginal expansion toward LMIC priorities. This imbalance restricts the generation of locally relevant evidence critical for effective preparedness and response. The  analysis also does not fully explore the underlying causes of these gaps, such as systemic barriers in funding mechanisms, limited institutional capacity, or geopolitical factors influencing resource allocation. In fact, expected declines in official development assistance and planned cuts in major donor countries may exacerbate these disparities, yet the dataset cannot predict or model these future impacts. In general, the reliance on grant-level data without linking to research outputs or outcomes limits the ability to evaluate whether funded projects translate into meaningful improvements for vulnerable populations or LMIC contexts. These constraints underscore the need for more comprehensive, equity-focused tracking and stronger coordination among global funders.

 These topics have to be considered considering the following comments to create consistency ion the paper.  First, authors have to synthesize the main results in a simple table to be clear for readers and then show what this study adds compared to other studies.  Although the Results section provides a detailed description of the data collected and analyzed, there needs to be a more critical synthesis and comparison of the findings with the literature. The discussion section has to interpret and describe the significance of your findings in relation to what was already known about the research problem being investigated and explain any new understanding or insights that emerged from your research. The discussion has better to connect to the introduction through the research questions, hypotheses, and the literature you reviewed. The discussion clearly has to explain how this study advances these research topics. Comment better on whether the results were expected for each set of findings; go into greater depth to explain unexpected findings.

 Moreover, either compare your results with the findings from other studies or use the studies to support your results.

  Conclusion has to be a separate section. Conclusion has not to be a summary, but authors have to focus on manifold limitations of this study. The Conclusion does not adequately discuss the theoretical and managerial implications of the study. Discuss how the gap in the literature has been addressed about the research problem. Describe lessons learned, proposing recommendations that can help improve the preparedness and prevention of infectious disease leading to pandemic crises. Highlighting specific best practices of health and research policy to face next pandemics, also with funding to overall health and healthcare sectors. Create 3 subsections in the Conclusion: 1) Theoretical Implications, 2) Health Policy Implications, and 3) Future Research.

  Overall, then, the paper is interesting, but the structure is too fragmentated, long and confusing. Theoretical framework is weak, and some results create confusion… structure of the paper has to be improved; study design, discussion and presentation of results have to be clarified using suggested comments.

  I kindly recommend revising the paper by thoroughly addressing all comments provided, including engaging with the suggested readings. These references should be carefully reviewed and appropriately incorporated into both the main text and the bibliography.

 Please note that failure to implement the suggested improvements may result in a vague and inconsistent  paper.

Are the rationale for, and objectives of, the Systematic Review clearly stated?

Partly

Is the statistical analysis and its interpretation appropriate?

Partly

If this is a Living Systematic Review, is the ‘living’ method appropriate and is the search schedule clearly defined and justified? (‘Living Systematic Review’ or a variation of this term should be included in the title.)

Not applicable

Are sufficient details of the methods and analysis provided to allow replication by others?

Partly

Are the conclusions drawn adequately supported by the results presented in the review?

Partly

Reviewer Expertise:

Pandemics, public health, pandemic preparedness, vaccination, health crisis

I confirm that I have read this submission and believe that I have an appropriate level of expertise to confirm that it is of an acceptable scientific standard, however I have significant reservations, as outlined above.

## References

[1] Holmer, H., Reid-Henry, S., Agyepong, I.A., Songane, F.F., Heymann, D.L. Pandemic financing should learn from revolving funds,Lancet, 405(10473), pp. 122–123.10.1016/S0140-6736(24)02633-339709980

[2] Kaawa-Mafigiri, D., Muyomba, D., Kisakye, I.S., Semakula, D., Sewankambo, N.K. 2025. Research and community engagement during the COVID- 19 pandemic in a resource-limited setting: a mixed methods study with epidemic preparedness implications.BMC Health Services Research, 25(1), 600.10.1186/s12913-025-12691-zPMC1202349840281635

[3] : Sources, diffusion and prediction in COVID-19 pandemic: lessons learned to face next health emergency. *AIMS Public Health* .2023;10(1) : 10.3934/publichealth.2023012145-168 10.3934/publichealth.2023012 PMC1009113537063362

[4] Hanney, S.R., Yazdizadeh, B. 2025. Health research versus the virus: strengthening systems, saving lives.Health Research Policy and Systems, 23(1), 95.10.1186/s12961-025-01354-4PMC1229135040713550

[5] : Effective health systems facing pandemic crisis: lessons from COVID-19 in Europe for next emergencies. *International Journal of Health Governance* .2024;29(2) : 10.1108/IJHG-02-2024-001389-111 10.1108/IJHG-02-2024-0013

[6] Bell, D., von Agris, J., Tacheva, B., Brown, G.W. 2025. Natural Spillover Risk and Disease Outbreaks: Is Over-Simplification Putting Public Health at Risk?Journal of Epidemiology and Global Health, 15(1), 65.10.1007/s44197-025-00412-yPMC1202177340272646

[7] Krammer, F., Barclay, W.S., Beer, M., ... Terregino, C., Thålin, C. 2025. Europe needs a sustainably funded influenza research and response network.Lancet Infectious Diseases, 25(4), pp. 369–372.10.1016/S1473-3099(25)00068-439978373

[8] : Pandemic Prevention: Lessons from COVID-19. *Encyclopedia* .2021;1(2) : 10.3390/encyclopedia1020036433-444 10.3390/encyclopedia1020036

[9] Elhakim, M., Elkholy, A., Stephen, M., Brennan, R.J., Zouiten, A. 2025. Two rounds of the Pandemic Fund at the WHO Eastern Mediterranean Region: progress, lessons learned, challenges, and way forward.Global Health Action, 18(1), 2475596.10.1080/16549716.2025.2475596PMC1206833140340513

# Response

**Published:** 2026-06-25

**Authors:** Alice Norton

**Affiliations:** University of Oxford, UK

We thank the reviewer for acknowledging the relevance of the topics covered. We agree that the structure and presentation required improvement, and we have undertaken a comprehensive revision of the manuscript in response to the specific comments that follow.

*For the title, w*e have replaced ‘infectious diseases with a pandemic potential’ with the more concise ‘pandemic-prone infectious diseases’, preserving all substantive elements while improving readability. For the abstract, we are limited by word count. We have revised the abstract to clearly highlight the key gaps and disparities identified by our analysis, including the concentration of investment in high-income countries, limited support for LMIC-led research, insufficient representation of vulnerable populations, and the relative underfunding of diagnostics, health systems research and ethics.

 We have also added a closing sentence to the Background to explicitly state the study’s aims. We have revisited the Introduction to further strengthen the articulation of our research questions and more explicitly identify the gap in the literature that this paper addresses. We have incorporated further relevant citations, including some from the suggested readings.

 Regarding the structure of the Methods section, the current organisation reflects the logical flow of the methodology and the sub-sections help guide readers through the different components of the methodology rather than create fragmentation. We have nonetheless reviewed the section and made minor adjustments to improve overall readability. On the placement of limitations, we consider their current position appropriate and intentional. As these limitations pertain specifically to the dataset, they provide important context for interpreting the results that follow. Relocating them to the conclusion would, in our view, deprive readers of this framing at the point where it is most needed. We also note that this structure is consistent with established practice in the COVID CIRCLE living review, from which Pandemic PACT directly builds. 

 We hope these clarifications and revisions address the reviewer’s concerns.

 We agree with the observation regarding the completeness and representativeness of the data set, and have highlighted them in the Limitations and Risk-of-bias subsections accordingly. We acknowledge that the Pandemic PACT database relies on publicly available information and voluntary reporting, which may underrepresent smaller funders, private sector contributions, and non-traditional sources of funding. At the same time, we have contextualised this limitation by noting that Pandemic PACT represents the most comprehensive resource of its kind currently available, and by describing the ongoing efforts to expand coverage and refine the dataset over time.

 We thank the reviewer for highlighting this important issue regarding geographic and institutional imbalance. We fully acknowledge this imbalance, as it is one of the key findings of our study. The concentration of funding in high-income countries and the limited participation of LMICs as recipients of funded research are reported and discussed in detail in the discussion section, where we also reflect on the implications for pandemic preparedness and equitable global health funding strategies. Regarding the categorisation framework, **a**s described in the methodology, grants can be assigned to multiple broad categories simultaneously, which allows interdisciplinary projects to be captured across relevant domains rather than constrained to a single classification. Further granularity is provided through subcategories.

 For instance, where information is available, clinical trials are classified according to their phase. This level of detail is presented in Table 2 and Extended Data 3, which provides a full breakdown of Pandemic PACT categories and corresponding subcategories. We hope this helpfully clarifies the categorisation approach. We fully agree that the 2020–2024 period represents an exceptional context, and we have been attentive to this throughout the manuscript. The paper acknowledges the extraordinary nature of the COVID-19-period, and our hope is that the living mapping review format, with regular updates, will help capture the evolving reality of research funding over time. In the Results section, COVID-19 was intentionally excluded from the disease-specific analysis precisely to avoid its outsized influence distorting patterns across other pandemic-prone diseases. This decision is acknowledged in the manuscript. Regarding the focus on financial commitments rather than research outputs, we agree this is an important consideration.

 We have added to the Discussion that our analysis does not assess implementation success, knowledge generation, or policy uptake, and we highlight the linkage of funding data to research outputs as a valuable direction for future work. We consider this, however, to be beyond the scope of the current review, which focuses on characterising the funding landscape itself.

 We appreciate the reviewer drawing attention to the ethical and social dimensions of research. We would like to clarify that the underrepresentation of ethics-related research and the limited inclusion of vulnerable populations are indeed among the key findings of our study and are discussed throughout the manuscript. As a descriptive mapping review, our aim is to characterise the funding landscape as it exists, and highlighting these gaps is very much part of our contribution. Exploring the underlying causes of these patterns or proposing specific interventions is beyond the scope of this work, but we agree these would be valuable directions for future research. On the dynamic nature of the database, we fully agree with the reviewer that continuous monitoring is essential. This is indeed central to the design of a living mapping review.

 The current analysis constitutes a baseline snapshot, and the living review format is specifically intended to allow trends to be tracked and conclusions updated as new data, emerging diseases, and shifting funding priorities evolve over time.

 We thank the reviewer for this comment and appreciate the methodological point raised regarding the high financial commitments in rich countries. However, the objective of this mapping review is to characterise the absolute landscape of pandemic preparedness research funding, documenting where financial resources are actually flowing, who is receiving them, and in what amounts. In this context, we believe that presenting absolute values is appropriate and consistent with established practice in research funding landscape analyses, which typically report absolute funding flows to characterise the distribution of investments. For example, a funder or policymaker seeking to understand where funding is being directed needs to know the actual amounts committed, rather than figures adjusted by population size, GDP, research expenditure, or other indicators. Normalisation by country-level indicators would change the nature of the analysis, shifting it from a funding landscape characterisation to a measure of relative research investment. While such an analysis is valuable in its own, it addresses a different research question from the one this study poses.

 We also note that our findings are presented not only in terms of financial commitments, but also the number of grants awarded (see Extended Data Figures). This metric is less sensitive to the influence of country size and therefore provides an alternative perspective on the distribution of research activity across countries. The broadly consistent patterns observed across both funding amounts and grant counts strengthen confidence in the interpretation of the findings. Finally, any normalisation approach would require selecting a specific denominator (e.g., population size, GDP, national R&D expenditure), each of which introduces different assumptions and may produce different interpretations. Given the descriptive objectives of this study, we believe that presenting absolute funding commitments provides the most transparent and policy-relevant representation of the funding landscape. To clarify this point for readers, we have added a brief note to the relevant figure legends indicating that figures present absolute funding commitments, so that comparisons across countries of different sizes should be interpreted accordingly.

 We have decided to leave all the figures within the main text due to their central relevance to the paper. We agree that the limited representation of ethnic minorities and other at-risk populations is central to the study. This is a key observation discussed in the manuscript. We would also like to clarify the methodological basis for the population categorisation used in the Pandemic PACT dataset. The population groups captured in the database were defined through a structured process involving a multidisciplinary working group with expertise in global health, infectious disease, and research funding, ensuring that the categorisation framework is both systematic and grounded in established practice. The breadth of this ontology reflects the range of sociodemographic groups considered relevant to pandemic-prone disease research across the diseases in scope. Against this backdrop, the finding that ethnic groups and vulnerable populations are minimally or entirely absent for most diseases is not a limitation of the categorisation framework itself, but rather a key finding about the state of the funded research portfolio. We further emphasise that these gaps highlight the need for more systematic inclusion of diverse population groups in research designs and funding strategies to support equitable pandemic preparedness.

 We agree that structural and data limitations may lead to an underestimation of LMIC-led research investments. As noted in the Discussion, the Pandemic PACT dataset relies primarily on publicly available funding information, and therefore some forms of funding, particularly intramural investments and smaller-scale initiatives that are not publicly reported, may not be captured. We explicitly acknowledge that this likely results in an underestimation of research investments originating from LMICs. These structural limitations are discussed alongside the broader finding that funding remains concentrated in high-income countries, and we highlight the need for stronger coordination and alignment with local research priorities.

 We agree that a range of structural and systemic factors may influence the distribution of research investments across countries. However, the objective of this study is to provide a descriptive mapping of the global landscape of research funding for infectious diseases with pandemic potential, based on publicly available data captured in the Pandemic PACT database. As such, the analysis focuses on identifying patterns and gaps in funding allocations rather than examining the underlying causes of these disparities.

 We agree that understanding the structural drivers of funding imbalances, as well as modelling the potential effects of future changes in official development assistance or donor priorities, represents an important area for future research. However, addressing these questions would require additional methodological approaches and data sources beyond the scope of the present mapping review. We have now clarified this point in the Discussion and note that structural factors, such as funding mechanisms, institutional capacity, and geopolitical considerations, may contribute to these observed patterns. We have revised the Discussion to more actively interpret our findings in the context of existing evidence, rather than only describing the data, and to clearly articulate the novel contributions of this analysis across the key dimensions covered (e.g., research areas, geography, disease focus, population covered).

 We have also ensured that the Discussion explicitly identifies what this study adds compared to prior work, including the COVID CIRCLE living review and other analyses of pandemic preparedness research funding. We have revised the Discussion to more explicitly connect back to the research questions and framing established in the introduction, and to more clearly articulate how the findings advance the research topics outlined at the introduction of the paper.

 The Conclusion has been revised as a separate, standalone section. We thank the reviewer for this overall assessment and for the detailed and constructive comments provided throughout the review. We have undertaken a comprehensive revision of the manuscript in response to all specific points raised, and we believe these changes collectively address the concerns about structure, clarity, and presentation. Specifically, we have revised the Introduction to better position this work within the existing literature.

 The Discussion has been rewritten to more actively interpret our findings in the context of prior evidence, to connect back to the research questions established in the introduction, and to more explicitly articulate the novel contributions of this analysis. The Conclusion has been revised as a standalone section that clearly summarises the key findings and their implications.
